# Synthesis and Stereochemical Determination of the Peptide Antibiotic Novo29

**DOI:** 10.1021/acs.joc.2c02648

**Published:** 2023-01-19

**Authors:** Maj Krumberger, Xingyue Li, Adam G. Kreutzer, Aaron J. Peoples, Anthony G. Nitti, Andrew M. Cunningham, Chelsea R. Jones, Catherine Achorn, Losee L. Ling, Dallas E. Hughes, James S. Nowick

**Affiliations:** † Department of Chemistry, University of California, Irvine, Irvine, California92697, United States; ‡ Department of Pharmaceutical Sciences, University of California, Irvine, Irvine, California92697, United States; § NovoBiotic Pharmaceuticals LLC, 767C Concord Avenue, Cambridge, Massachusetts02138, United States

## Abstract

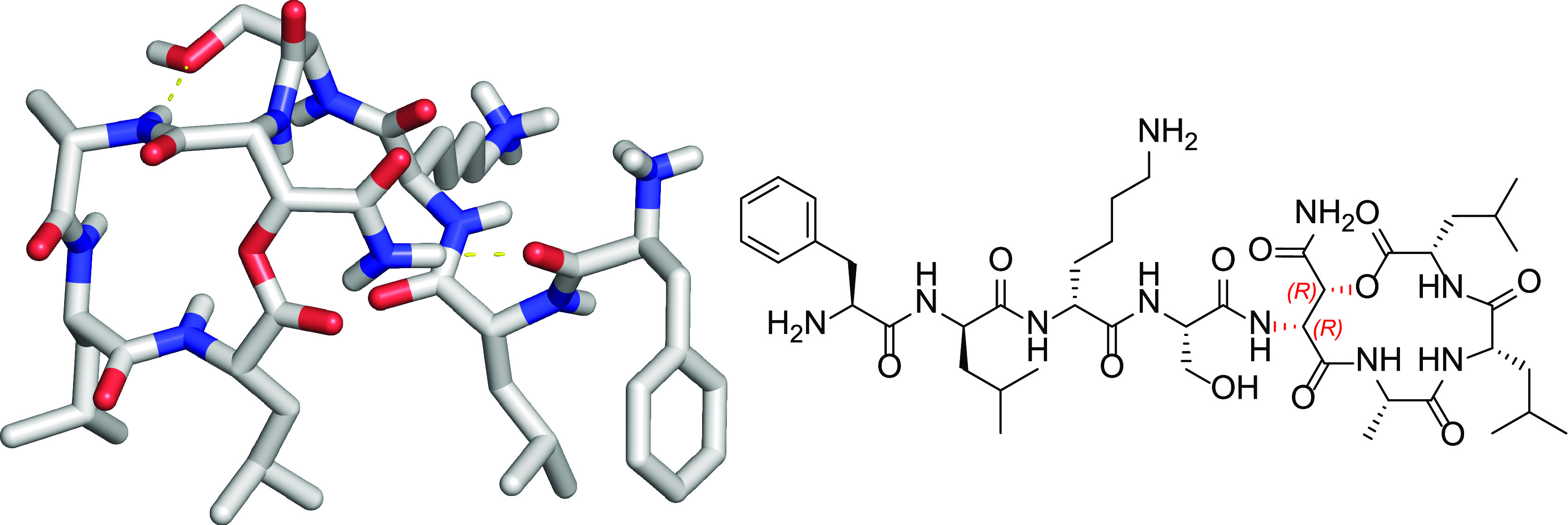

This paper describes the synthesis and stereochemical determination of Novo29 (clovibactin), a new peptide antibiotic that is related to teixobactin and is active against Gram-positive bacteria. Novo29 is an eight-residue depsipeptide that contains the noncanonical amino acid hydroxyasparagine of hitherto undetermined stereochemistry in a macrolactone ring. The amino acid building blocks Fmoc-(2*R*,3*R*)-hydroxyasparagine-OH and Fmoc-(2*R*,3*S*)-hydroxyasparagine-OH were synthesized from (*R*,*R*)- and (*S*,*S*)-diethyl tartrate. Novo29 and *epi*-Novo29 were then prepared by solid-phase peptide synthesis using these building blocks. Correlation with an authentic sample of Novo29 through ^1^H NMR spectroscopy, LC-MS, and *in vitro* antibiotic activity established that Novo29 contains (2*R*,3*R*)-hydroxyasparagine. X-ray crystallography reveals that *epi*-Novo29 adopts an amphiphilic conformation, with a hydrophobic surface and a hydrophilic surface. Four sets of *epi*-Novo29 molecules pack in the crystal lattice to form a hydrophobic core. The macrolactone ring adopts a conformation in which the main-chain amide NH groups converge to create a cavity, which binds ordered water and acetate anion. The amphiphilic conformation of *epi*-Novo29 is reminiscent of the amphiphilic conformation adopted by the related antibiotic teixobactin and its derivatives, which contains a hydrophobic surface that interacts with the lipids of the bacterial cell membrane and a hydrophilic surface that interacts with the aqueous environment. Molecular modeling suggests that Novo29 can adopt an amphiphilic conformation similar to teixobactin, suggesting that Novo29 may interact with bacteria in a similar fashion to teixobactin.

## Introduction

Novo29, a new antibiotic from a soil bacterium closely related to *Eleftheria terrae*, was recently reported.^1^ Novo29 is an eight-residue depsipeptide comprising a macrolactone ring and a linear tail. It is active against Gram-positive bacteria, including drug-resistant human pathogens, such as MRSA and VRE. Novo29 kills bacteria by inhibiting bacterial cell-wall synthesis, with no detectable resistance occurring upon serial passaging.^2,3^ Although the amino acid sequence of Novo29 was determined, the stereochemistry of the rare noncanonical amino acid hydroxyasparagine at position 5 was not able to be determined. Neither NMR spectroscopic analysis nor correlation with authentic hydroxyasparagine of known stereochemistry has thus far been feasible, leaving open the question of which hydroxyasparagine stereoisomer constituted the natural product.

**Figure 1:**
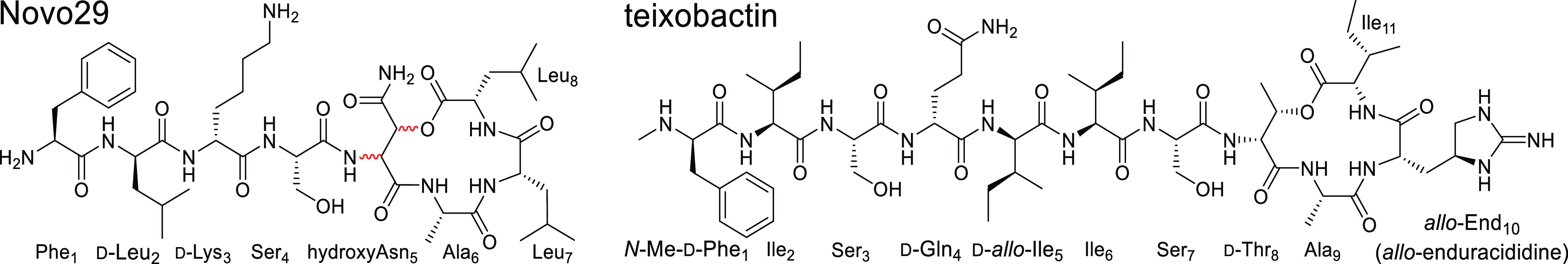
Structures of Novo29 and teixobactin. The unassigned stereochemistry of hydroxyAsn at position 5 of Novo29 is highlighted in red.

Novo29 is related in structure to teixobactin, which is produced by *E. terrae*, but it is smaller, containing eight residues instead of eleven (Figure 1).^2,3^ Like teixobactin, Novo29 exhibits good activity against Gram-positive bacteria and targets cell-wall precursors. Novo29 is a promising antibiotic drug candidate, because it kills drug-resistant pathogens without detectable resistance and exhibits reduced propensity to form gels upon intravenous dosing.^4^ In the current study, we establish the stereochemistry of the hydroxyasparagine (hydroxyAsn) residue at position 5 and confirm the structure of Novo29 through chemical synthesis and spectroscopic and functional correlation. We also report the X-ray crystallographic structure of a hydroxyAsn epimer of Novo29 (*epi*-Novo29), which may provide insights into how Novo29 binds bacterial cell-wall precursors.

## Results and Discussion

### Synthesis of Novo29 and *epi*-Novo29

**Figure 2:**
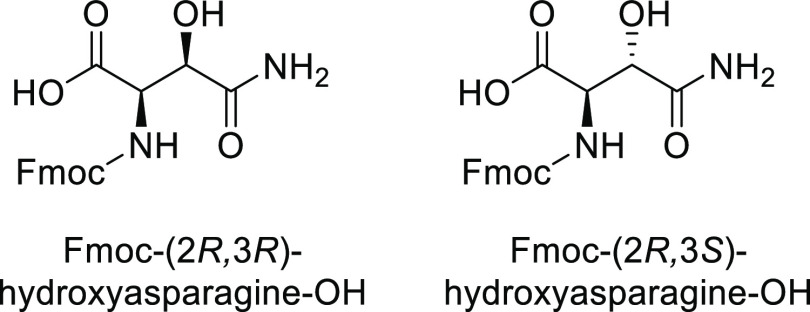
Structures of Fmoc-(2*R*,3*R*)-hydroxyasparagine-OH and Fmoc-(2*R*,3*S*)-hydroxyasparagine-OH.

We hypothesized the stereochemistry at position 5 to be (2*R,*3*R*)-hydroxyAsn, based on the similarity in structure and connectivity of d-Thr_8_ of teixobactin, as well as the related depsipeptide antibiotic hypeptin.^5^ We developed and carried out the synthesis of a suitably protected (2*R,*3*R*)-hydroxyAsn as a building block that could be readily incorporated into solid-phase peptide synthesis (SPPS). This building block is Fmoc-protected at the α-amino position; the hydroxyl and primary amide groups can tolerate SPPS without protection.^6,7^ For comparison, we also synthesized the Fmoc-protected (2*R*,3*S*)-hydroxyAsn diastereomer (Figure 2).

**Figure 3:**
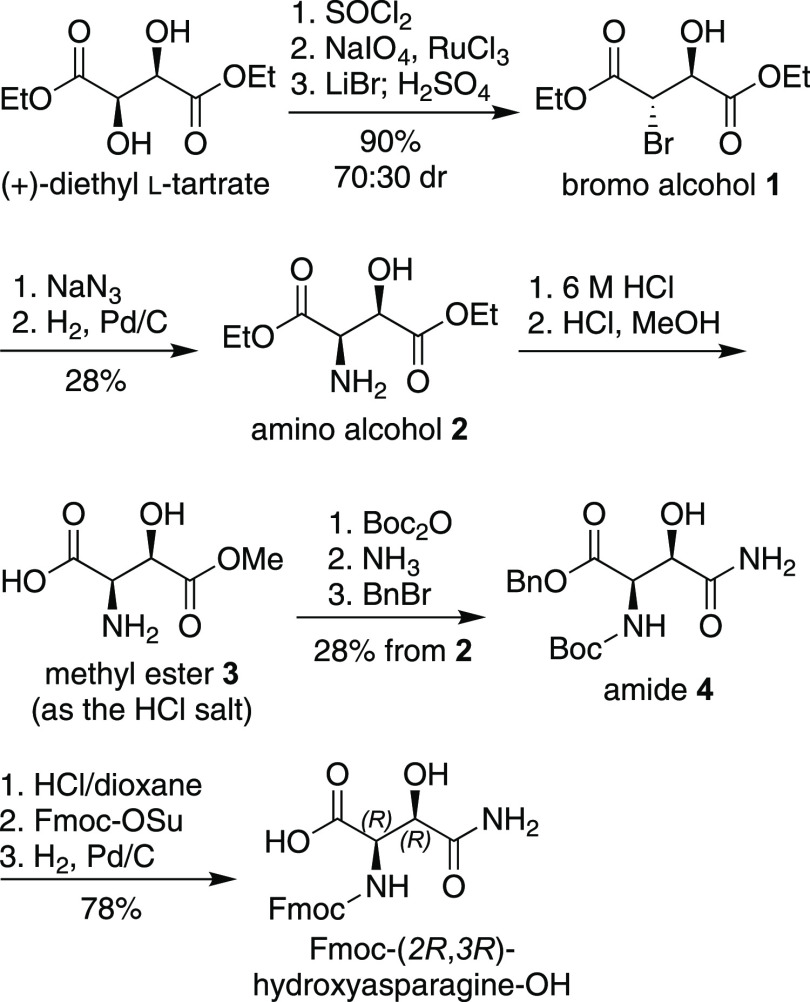
Synthesis of Fmoc-(2*R*,3*R*)-hydroxyasparagine-OH.

We synthesized Fmoc-(2*R*,3*R*)-hydroxyasparagine-OH from (+)-diethyl l-tartrate as outlined in Figure 3. (2*R*,3*R*)-(+)-Diethyl l-tartrate was converted to bromo alcohol **1** by conversion to the cyclic sulfite and oxidation to the cyclic sulfate followed by ring opening with LiBr.^8^ This sequence was previously established for diisopropyl and dimethyl tartrates and resulted in the formation of 80:20 and 85:15 mixtures of diastereomers.^9,10^ In our hands the LiBr reaction proceeded with the formation of the two diastereomers of bromo alcohol **1** in a 70:30 ratio favoring the desired diastereomer (Figure S1). Treatment of the mixture of diastereomers with sodium azide, to give the corresponding azido alcohols with inversion of stereochemistry, followed by reduction with H_2_ and Pd/C and chromatographic separation of diastereomers afforded amino alcohol **2**.

Amino alcohol **2** was converted to methyl ester **3** by hydrolysis of the ethyl ester groups in 6 M HCl followed by regioselective differentiation of the two carboxylic acid groups by Fischer esterification with HCl in CH_3_OH.^11^ The methyl esterification proceeds regioselectively, possibly because the ammonium group at the 2-position deactivates the carboxylic acid group at the 1-position.^12^ Methyl ester **3** was then converted to amide **4** by Boc protection of amino group at the 2-position, ammonolysis to the amide at the 4-position, and benzyl ester protection of the carboxylic acid at the 1-position. Amide **4** was subsequently converted to Fmoc-(2*R*,3*R*)-hydroxyasparagine-OH by removal of the Boc group with 4 M HCl in dioxane, Fmoc protection of the amino group with Fmoc-OSu, and hydrogenolysis of the benzyl ester group with H_2_ and Pd/C.

**Figure 4:**
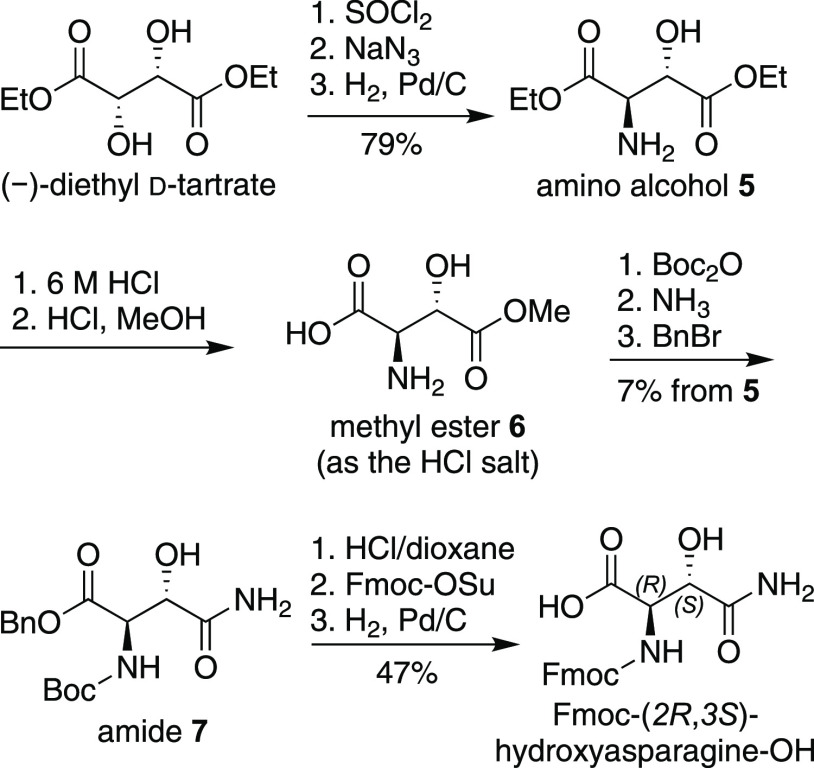
Synthesis of Fmoc-(2*R*,3*S*)-hydroxyasparagine-OH.

We synthesized the Fmoc-(2*R*,3*S*)-hydroxyasparagine-OH diastereomer from (-)-diethyl d-tartrate in a related fashion, as outlined in Figure 4. (2*S*,3*S*)-(-)-Diethyl d-tartrate was converted to amino alcohol **5** by conversion to the cyclic sulfite with thionyl chloride, followed by ring opening with sodium azide and reduction of the azido group with H_2_ and Pd/C.^13^ Amino alcohol **5** was then converted to Fmoc-(2*R*,3*S*)-hydroxyasparagine-OH in a similar fashion to that described above for amino alcohol **2**.

**Figure 5:**
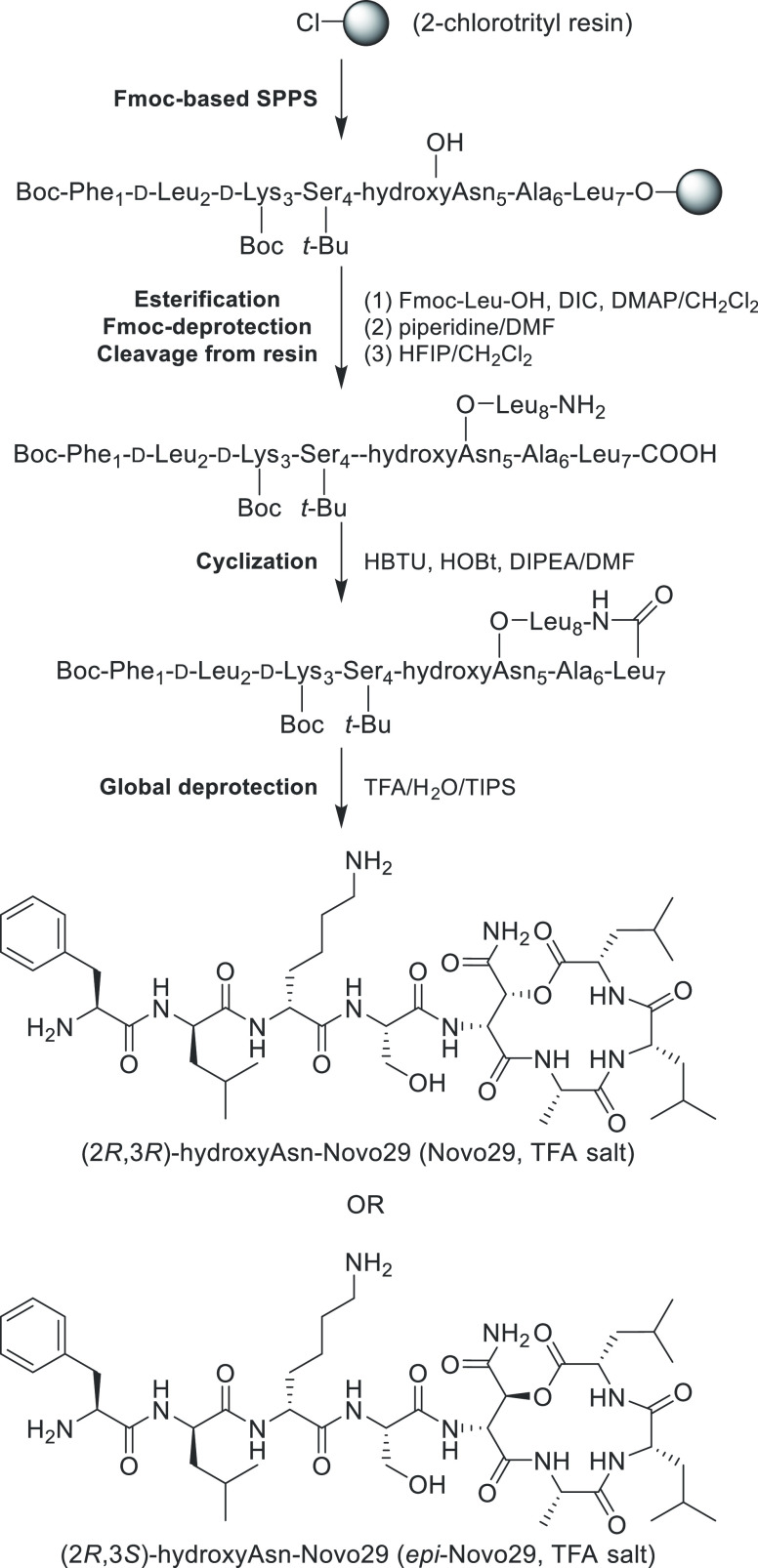
Synthesis of Novo29 and *epi*-Novo29.

We determined the stereochemistry of Novo29 by synthesizing (2*R*,3*R*)-hydroxyAsn-Novo29 and (2*R*,3*S*)-hydroxyAsn-Novo29 and then correlating these synthetic peptides with natural Novo29 by ^1^H NMR spectroscopy, LC-MS analysis, and MIC assays. (2*R*,3*R*)-hydroxyAsn-Novo29 and (2*R*,3*S*)-hydroxyAsn-Novo29 were synthesized by solid-phase peptide synthesis of a protected acyclic precursor followed by solution-phase cyclization, in a fashion similar to that which we have developed for the synthesis of teixobactin analogues (Figure 5).^6,14−18^ The synthesis begins with loading Fmoc-Leu-OH (position 7) onto 2-chlorotrityl resin, followed by incorporation of residues 6 through 1. Leu_8_ was esterified onto the β-hydroxy group of the (2*R*,3*R*)-hydroxyAsn or (2*R*,3*S*)-hydroxyAsn residue at position 5 by treatment with Fmoc-Leu-OH, DIC, and DMAP.^19^ Fmoc deprotection of Leu_8_ and cleavage of the peptide from the resin with 20% hexafluoroisopropanol (HFIP) in CH_2_Cl_2_ afforded the protected peptide with a free carboxylic acid group on Leu_7_ and a free amino group on Leu_8_. Cyclization between Leu_8_ and Leu_7_ was achieved with HBTU and HOBt.^20^ Global deprotection with trifluoroacetic acid (TFA) and reverse-phase HPLC purification yielded (2*R*,3*R*)-hydroxyAsn-Novo29 and (2*R*,3*S*)-hydroxyAsn-Novo29 as the trifluoroacetate salts.

### Stereochemical determination of Novo29

**Figure 6:**
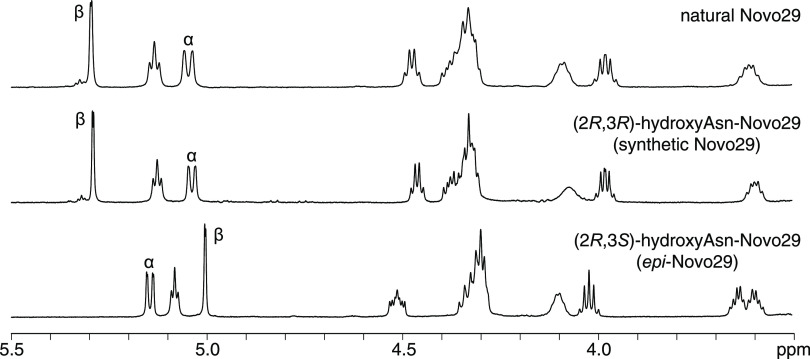
^1^H NMR spectra of natural Novo29, synthetic Novo29, and *epi*-Novo29 (3.5–5.5 ppm expansion, 500 MHz, 2 mM in DMSO-*d*_6_). The α- and β-protons of hydroxyAsn are labeled.

The ^1^H NMR spectrum of natural Novo29 in DMSO-*d*_6_ matches that of (2*R*,3*R*)-hydroxyAsn-Novo29 and differs significantly from that of synthetic (2*R*,3*S*)-hydroxyAsn-Novo29 (Figure 6). Notably, the α- and β-proton resonances of the hydroxyAsn residue in natural and (2*R*,3*R*)-hydroxyAsn-Novo29 both appear at 5.04 and 5.29 ppm, respectively, while those of (2*R*,3*S*)-hydroxyAsn-Novo29 appear at 5.14 and 5.00 ppm. From here on (2*R*,3*R*)-hydroxyAsn-Novo29 will be referred to as Novo29, and (2*R*,3*S*)-hydroxyAsn-Novo29 will be referred to as *epi*-Novo29. The amide NH region of both natural and synthetic Novo29 also match reasonably well and differ substantially from that of *epi*-Novo29. Surprisingly, the chemical shifts of the NH groups of Novo29 proved sensitive to concentration, varying by as much as 0.1 ppm over concentrations from 1 to 3 mM. This observation suggests that even in DMSO, Novo29 undergoes relatively strong self-association (Figure S2 and Table S1).

To corroborate the stereochemical identity of Novo29 we compared natural Novo29 to synthetic Novo29 and *epi*-Novo29 by LC-MS. With a gradient of 3–27% CH_3_CN over 25 min on a C4 column, natural and synthetic Novo29 elute comparably (15.81 and 15.88 min, respectively), whereas *epi*-Novo29 elutes substantially later (16.57 min). The retention time and peak shape of Novo29 proved highly concentration dependent, with broad peaks and shorter elution times resulting at higher concentrations, again suggesting strong self-association (Figure S3).

To further corroborate the stereochemical identity of Novo29, and to evaluate the importance of the stereochemistry of the hydroxyAsn residue, we tested the antibiotic activity of natural Novo29, synthetic Novo29, and *epi*-Novo29 using minimum inhibitory concentration (MIC) assays against two Gram-positive bacteria, *Bacillus subtilis*, and *Staphylococcus epidermidis*. We used the Gram-negative bacterium *Escherichia coli* as a negative control. Natural Novo29 and synthetic Novo29 exhibit comparable antibiotic activity against the Gram-positive bacteria, with MIC values of 0.125 μg/mL for *B. subtilis* and 0.25 μg/mL for *S. epidermidis* (Table 1). To our surprise, both synthetic Novo29 and natural Novo29 also exhibited modest activity against *E. coli*, with MIC values of 8 μg/mL.^21^ In contrast, *epi*-Novo29 exhibited no MIC activity (>32 μg/mL) against any of the bacteria, thus indicating that the 2*R*,3*R* stereochemistry of the hydroxyAsn residue is critical to the antibiotic activity of Novo29.

**Table 1:** MIC values of natural Novo29, synthetic Novo29, and *epi*-Novo29 in μg/mL

	*Bacillus subtilis*ATCC 6051	*Staphylococcus epidermidis*ATCC 14990	*Escherichia coli*ATCC 10798
natural Novo29	0.125	0.25	8
synthetic Novo29	0.125	0.25	8
*epi*-Novo29	>32	>32	>32

### Crystallographic Studies of *epi*-Novo29 and a Molecular Model of Novo29

X-ray crystallography permitted the structural elucidation of *epi*-Novo29 and may provide insights into the conformation and mechanism of action of Novo29. Both *epi*-Novo29 and Novo29 were screened for crystallization in a 96-well plate format using crystallization kits from Hampton Research (PEG/Ion, Index, and Crystal Screen). Novo29 did not form crystals from any of the conditions tested. *epi*-Novo29 formed rod-shaped crystals from 2.8 M sodium acetate at pH 7.0. Further optimization in a 24-well plate format afforded long rectangular crystals suitable for X-ray diffraction from 2.8 M sodium acetate at pH 6.6. Diffraction data were initially collected using an X-ray diffractometer on an *epi*-Novo29 crystal that was soaked in KI to incorporate iodide ions into the lattice (PDB 8CUF). The crystallographic phases were determined using single-wavelength anomalous diffraction (SAD) phasing from the incorporated iodide ions. Higher-resolution diffraction data were subsequently collected out to 1.13 Å resolution on an unsoaked *epi*-Novo29 crystal using a synchrotron (PDB 8CUG). The crystallographic phases of the higher-resolution data were determined by molecular replacement using the KI-soaked structure as a search model. The asymmetric unit contains two molecules of *epi*-Novo29, which exhibit only minor differences in conformation.

**Figure 7:**
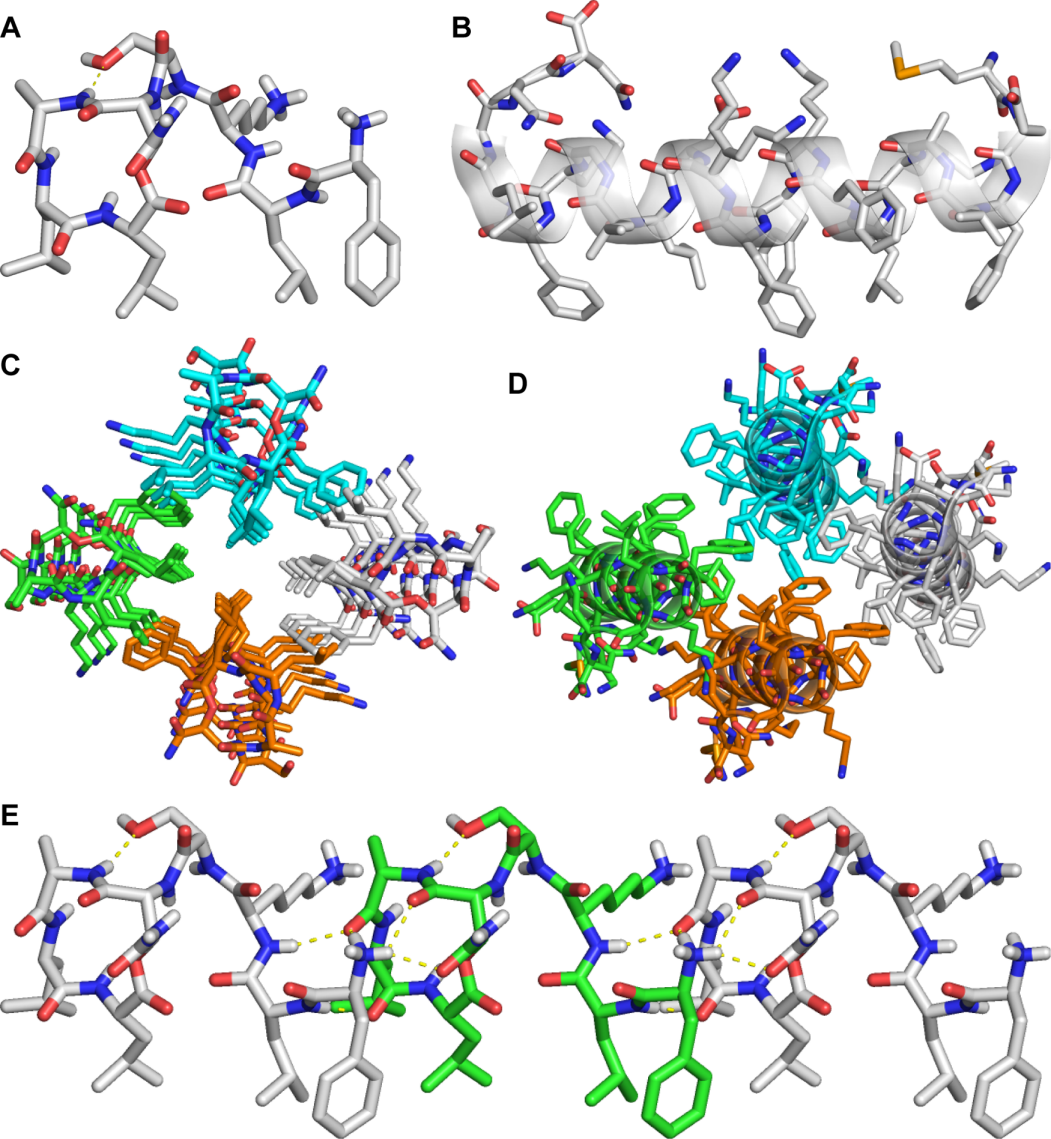
(A) X-ray crystallographic structure of *epi*-Novo29 (PDB 8CUG). (B) X-ray crystallographic structure of PSMα3 (PDB 5I55), illustrating the relationship of this amphiphilic 22-residue α-helical peptide to *epi*-Novo29. (C) Crystal packing of *epi*-Novo29. Molecules assemble in columns in the crystal lattice, with four columns of molecules arranged in a hydrophobic cluster through packing of Phe_1_, d-Leu_2_, Leu_7_, and Leu_8_. (D) Crystal packing of PSMα3, illustrating the relationship to the crystal packing of *epi*-Novo29. (E) Assembly of *epi*-Novo29 in the crystal lattice, illustrating the intermolecular hydrogen-bonding between molecules comprising the columns. Three molecules are shown.

In the X-ray crystallographic structure, *epi*-Novo29 adopts an amphiphilic conformation, with the side chains of Phe_1_, d-Leu_2_, Leu_7_, and Leu_8_ creating a hydrophobic surface and the side chains of d-Lys_3_, Ser_4_, and (2*R*,3*S*)-hydroxyAsn_5_, as well as the *N*-terminal ammonium group, creating a hydrophilic surface (Figure 7A). An intramolecular hydrogen bond between the main-chain NH group of Ala_6_ and the side chain OH group of Ser_4_ helps enforce this conformation. In the lattice, *epi*-Novo29 packs so that the hydrophobic surfaces come together, with four sets of *epi*-Novo29 molecules forming a hydrophobic core (Figure 7C). The macrolactone ring of *epi*-Novo29 adopts a conformation in which the main-chain NH groups of (2*R*,3*S*)-hydroxyAsn_5_, Ala_6_, Leu_7_, and Leu_8_ converge to create a cavity, which binds ordered water and acetate anion.

The amphiphilic structure of *epi*-Novo29 is reminiscent of the amphiphilic structure our laboratory has previously observed for teixobactin derivatives, in which the side chains of *N*-methyl-d-Phe_1_, Ile_2_, d-*allo*-Ile_5_, and Ile_6_ create a hydrophobic surface and the side chains of Ser_3_, d-Gln_4_, and Ser_7_ create a hydrophilic surface.^6,18^ In teixobactin, this conformation is biologically significant, providing a hydrophobic surface that interacts with the lipids of the bacterial cell membrane and a hydrophilic surface to interact with the aqueous environment.^18,22−24^ In Novo29, the amphiphilic structure likely provides similar opportunity for interaction with the bacterial cell membrane. In teixobactin, the cavity created by the macrolactone ring binds the pyrophosphate groups of lipid II and related bacterial cell-wall precursors. The macrolactone ring of Novo29 has the potential to bind the pyrophosphate groups of lipid II in a similar fashion.

The amphiphilic structure of *epi*-Novo29 is similar to that of the α-helices of phenol-soluble modulin α3 (PSMα3), a cytotoxic 22-residue peptide secreted by *S. aureus* which aggregates to form novel “cross-α” amyloid fibrils.^25^ The α-helices formed by PSMα3 present phenylalanine and leucine residues on one surface, and polar residues on the other surface (Figure 7B). PSMα3 packs so that the hydrophobic surfaces come together in extended layers, in which the packing of four PSMα3 molecules resembles that of four *epi*-Novo29 molecules (Figure 7D). Although *epi*-Novo29 is much smaller than PSMα3 (8 residues instead of 22 residues) the *epi*-Novo29 molecules daisy-chain through intermolecular hydrogen bonding to form extended structures that resemble the larger α-helices of PSMα3 (Figure 7E).

**Figure 8:**
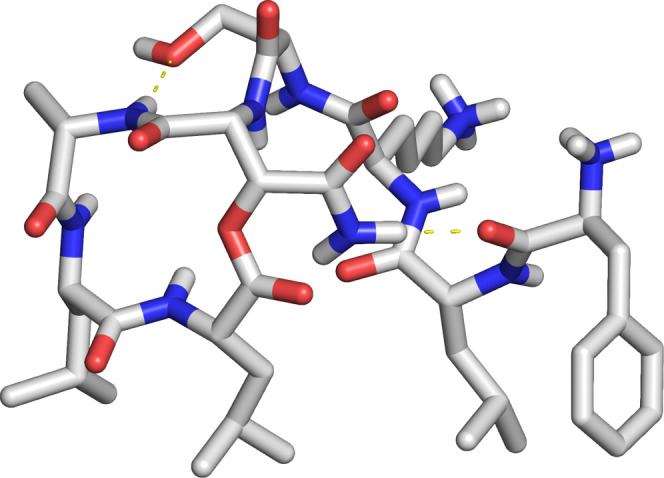
Molecular model of Novo29, based on the X-ray crystallographic structure of *epi*-Novo29. Intramolecular hydrogen bonds (yellow dashed lines) help enforce a preorganized conformation, in which the side chains of Phe_1_, d-Leu_2_, Leu_7_, and Leu_8_ align and can interact with the cell membrane of Gram-positive bacteria, and the macrolactone ring adopts a conformation that can bind the pyrophosphate groups of lipid II and related cell-wall precursors.

To gain additional insights into the mechanism of action of Novo29, we used the X-ray crystallographic structure of *epi*-Novo29 to create a molecular model of Novo29. Inversion of the *3S*-stereocenter of the (2*R*,3*S*)-hydroxyAsn residue of the crystal structure, followed by geometry optimization of only the side chain of the resulting (2*R*,3*R*)-hydroxyAsn residue, afforded a crystallographically based molecular model of Novo29 (Figure 8). In this model, the molecule still adopts an amphiphilic conformation. The only difference in structure is that the side-chain amide NH group of the (2*R*,3*R*)-hydroxyAsn residue hydrogen bonds to the carbonyl group of Phe_1_. This intramolecular hydrogen bond, in addition to the intramolecular hydrogen bond between the main-chain NH group of Ala_6_ and the side chain OH group of Ser_4_ helps enforce the amphiphilic conformation.

The X-ray crystallographic structure of *epi*-Novo29 and the crystallographically based molecular model of Novo29 suggest that Novo29 might be able to act upon Gram-positive bacteria in a fashion similar to teixobactin. Teixobactin adopts an amphiphilic conformation on bacteria, in which the side chains of *N*-methyl-d-Phe_1_, Ile_2_, d-*allo*-Ile_5_, and Ile_6_ insert into the cell membrane and the macrolactone ring binds the pyrophosphate groups of lipid II and related cell-wall precursors.^22^ The teixobactin molecules form hydrogen-bonded dimers that further assemble on the cell membrane through β-sheet formation, causing lipid II and related cell-wall precursors to cluster and ultimately lyse the bacteria. We envision that Novo29 also adopts an amphiphilic conformation on Gram-positive bacteria, in which the side chains of Phe_1_, d-Leu_2_, Leu_7_, and Leu_8_ insert into the cell membrane and the macrolactone ring binds the pyrophosphate groups of lipid II and related cell-wall precursors. The Novo29 molecules may further assemble through side-to-side hydrophobic interactions and end-to-end hydrogen bonding, and thus cause lipid II and related cell-wall precursors to cluster. We anticipate that the studies described in this paper will help lay the groundwork for testing this hypothesis.

## Conclusion

The antibiotic Novo29 has 2*R*,3*R* stereochemistry in the hydroxyAsn residue at position 5. Novo29 and diastereomer *epi*-Novo29 are prepared by Fmoc-based solid-phase peptide synthesis followed by solution-phase cyclization. The corresponding amino acid building blocks Fmoc-(2*R*,3*R*)-hydroxyasparagine-OH and Fmoc-(2*R*,3*S*)-hydroxyasparagine-OH are prepared from (*R*,*R*)- and (*S*,*S*)-diethyl tartrate. Correlation of synthetic Novo29 and *epi*-Novo29 with natural Novo29 through NMR spectroscopy, LC-MS, and MIC assays establishes the 2*R*,3*R* stereochemistry of the hydroxyAsn residue and confirms that Novo29 is active against Gram-positive bacteria. X-ray crystallography of *epi*-Novo29 reveals an amphiphilic conformation and packing of the molecules through hydrophobic and hydrogen-bonding interactions. Molecular modeling suggests that Novo29 should be able to adopt a similar amphiphilic conformation that is further stabilized through an additional hydrogen bond between the primary amide group of the (2*R*,3*R*)-hydroxyAsn residue and the carbonyl group of the phenylalanine residue.

## Data Availability

The data supporting this article are available in the published article and its Supporting Information.

## Supporting Information Available

Supporting text, figures, and tables: assignment of stereochemistry for amino alcohol **2**, NMR spectra of NH regions of natural Novo29 at differing concentrations, LC-MS data of natural Novo29, synthetic Novo29, and *epi*-Novo29, X-ray crystallographic statistic for *epi*-Novo29, synthetic procedures, HPLC, MS, and NMR characterization data (PDF)
