# Supplementary material for: Synthesis and Stereochemical Determination of the Peptide Antibiotic Novo29
Source: J Org Chem. 2023 Jan 19;88(4):2214–20. doi: 10.1021/acs.joc.2c02648 (PMC9942206; doi:10.1021/acs.joc.2c02648)

Supporting information for:

Synthesis and Stereochemical Determination of the Peptide Antibiotic Novo29

Maj Krumberger, Xingyue Li, Adam G. Kreutzer, Aaron J. Peoples, Anthony G. Nitti, Andrew M. Cunningham, Chelsea R. Jones, Catherine Achorn, Losee L. Ling, Dallas E. Hughes, James S. Nowick*
Corresponding author e-mail address: jsnowick@uci.edu

Table of Contents

Figure S1 .... **S3**
Figure S2 .... **S4**
Table S1 .... **S5**
Figure S3 .... **S6**
Table S2 – crystallographic properties of PDB 8CUG **S7**
Table S3 – crystallographic properties of PDB 8CUF **S8**
Materials and Methods **S9**
Chemical synthesis of Fmoc-(2*R*,3*R*)-hydroxyAsn-OH **S10**
Bromo alcohol **1** S10
Amino alcohol **2** S12
Methyl ester **3** S13
Amide **4** S15
Fmoc-(2*R*,3*R*)-hydroxyAsn-OH S17
Chemical synthesis of Fmoc-(2*R*,3*S*)-hydroxyAsn-OH **S19**
Amino alcohol **5** S19
Methyl ester **6** S20
Amide **7** S22
Fmoc-(2*R*,3*S*)-hydroxyAsn-OH S24
Synthesis of Novo29 and *epi*-Novo29 .... **S25**
NMR spectroscopic studies of Novo29 and *epi*-Novo29 .... **S28**
MIC assays **S29**
X-ray crystallography of *epi*-Novo29 .... **S30**
References **S32**
Characterization Data **S34**
Characterization of natural Novo29 .... **S34**
Analytical HPLC trace of natural Novo29 .... S34
Mass spectrum of natural Novo29 .... S35
Characterization of synthetic Novo29 .... **S36**

Analytical HPLC trace of synthetic Novo29 .... S36
Mass spectrum of synthetic Novo29 .... S37
Characterization of *epi*-Novo29 .... S38
Analytical HPLC trace of *epi*-Novo29 .... S38
Mass spectrum of *epi*-Novo29 .... S39
NMR spectra of intermediates S40
^{1}H NMR spectrum of bromo alcohol **1** S40
^{13}C NMR spectrum of bromo alcohol **1** S41
^{1}H NMR spectrum of amino alcohol **2** S42
^{13}C NMR spectrum of amino alcohol **2** S43
^{1}H NMR spectrum of methyl ester **3** S44
^{13}C NMR spectrum of methyl ester **3** S45
^{1}H NMR spectrum of amide **4** S46
^{13}C NMR spectrum of amide **4** S47
^{1}H NMR spectrum of Fmoc-(2*R*,3*R*)-hydroxyAsn-OH S48
^{13}C NMR spectrum of Fmoc-(2*R*,3*R*)-hydroxyAsn-OH S49
EXSY spectrum of Fmoc-(2*R*,3*R*)-hydroxyAsn-OH S50
^{1}H NMR spectrum of amino alcohol **5** S51
^{13}C NMR spectrum of amino alcohol **5** S52
^{1}H NMR spectrum of methyl ester **6** S53
^{13}C NMR spectrum of methyl ester **6** S54
^{1}H NMR spectrum of amide **7** S55
^{13}C NMR spectrum of amide **7** S56
^{1}H NMR spectrum of Fmoc-(2*R*,3*S*)-hydroxyAsn-OH S57
^{13}C NMR spectrum of Fmoc-(2*R*,3*S*)-hydroxyAsn-OH S58
EXSY spectrum of Fmoc-(2*R*,3*S*)-hydroxyAsn-OH S59
NMR spectra and chemical shift assignments of natural Novo29 .... S60
^{1}H NMR spectrum of natural Novo29 .... S60
TOCSY NMR spectrum of natural Novo29 .... S61
NOESY NMR spectrum of natural Novo29 .... S62
Table S4 – chemical shift assignments of natural Novo29 .... S63
^{1}H NMR spectrum of synthetic Novo29 .... S64
Table S5 – chemical shift comparison between natural and synthetic Novo29 .... S65
^{1}H NMR spectrum of *epi*-Novo29 .... S66

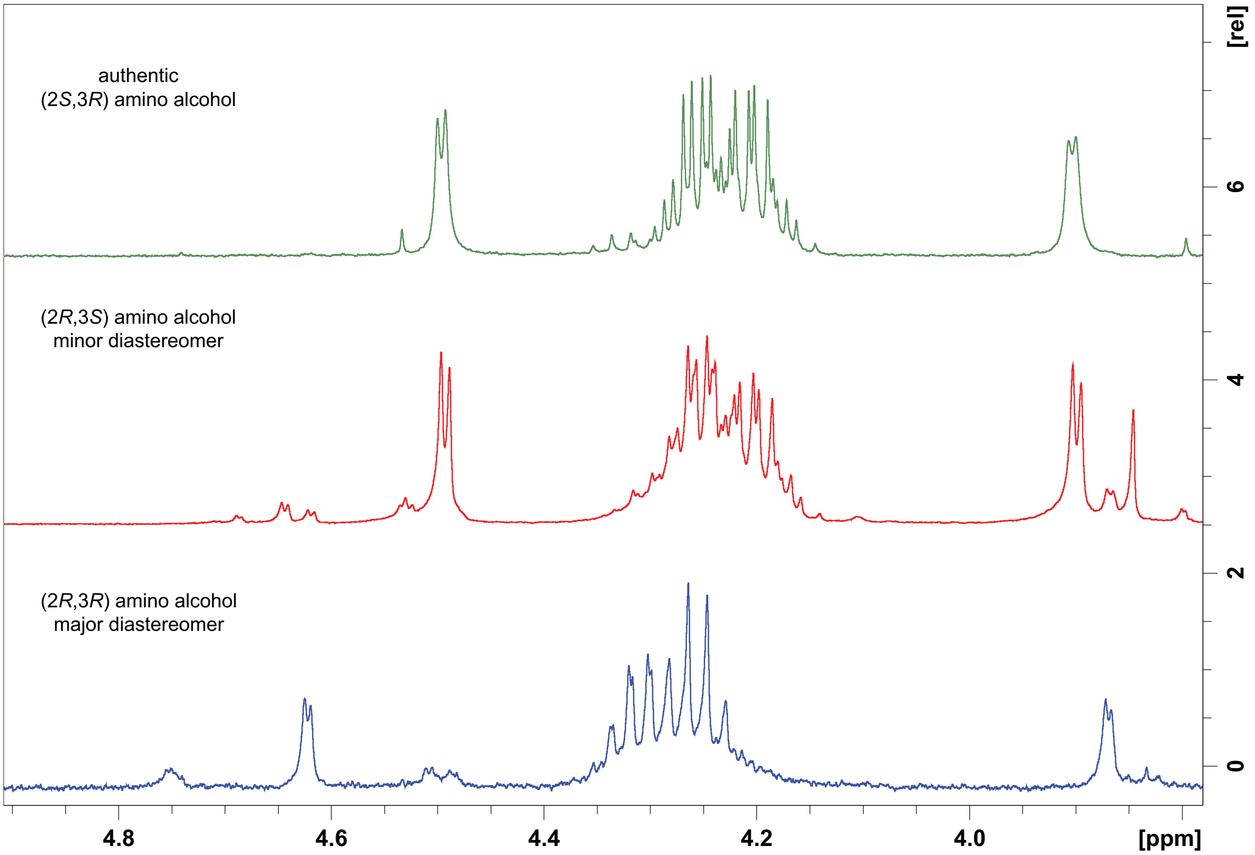

Figure S1. Establishment of (2*R*,3*R*) stereochemistry of amino alcohol **2** and the (2*S*,3*R*) stereoisomer by ^{1}H NMR spectroscopic correlation with an authentic sample of the (2*S*,3*R*) stereoisomer (the enantiomer of amino alcohol **5**) that was synthesized from (+)-diethyl L-tartrate by the route shown in Figure 4 of the main manuscript. Amino alcohol **2** was synthesized as shown in Figure 3 as a 70:30 mixture of diastereomers, which was then separated by column chromatography. Expansions of the ^{1}H NMR spectra of the major diastereomer (2*R*,3*R*, blue) and minor diastereomer (2*S*,3*R*, red) are shown. Correlation with an authentic sample of the (2*S*,3*R*) diastereomer (green) establishes that the minor diastereomer of amino alcohol **2** is (2*S*,3*R*), and thus that the major diastereomer is (2*R*,3*R*).

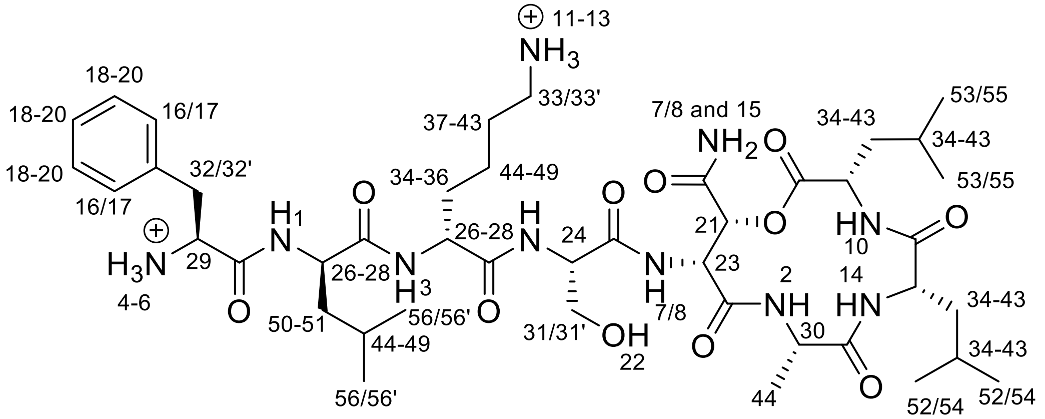

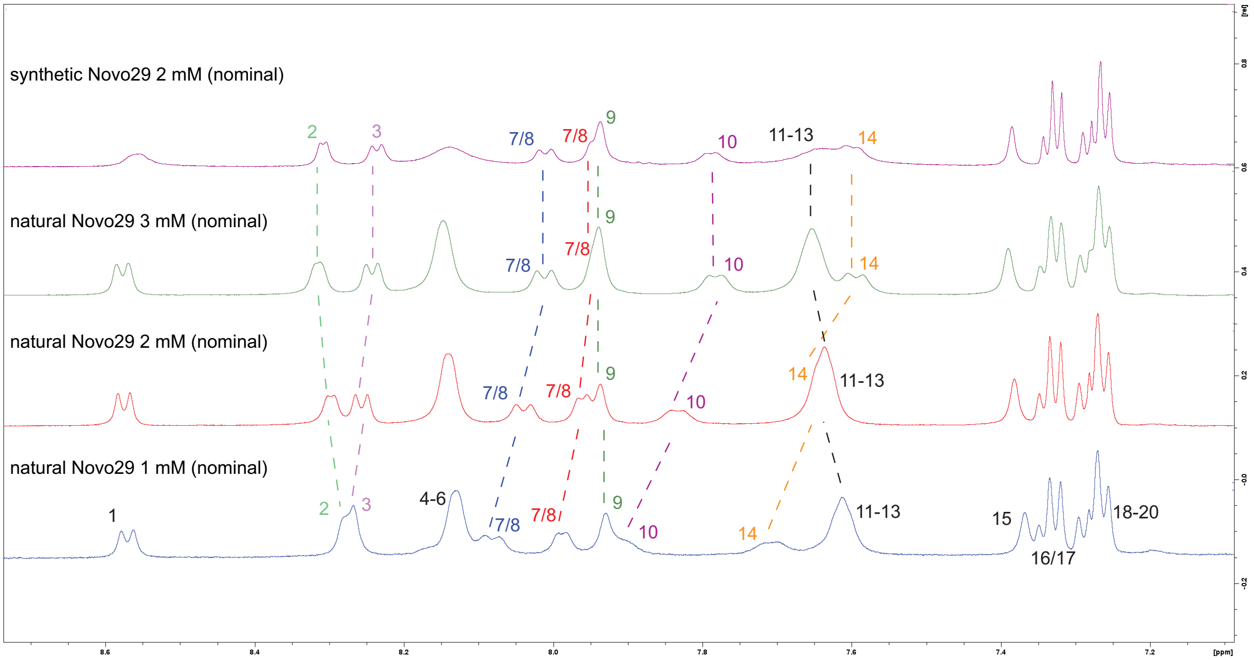

Figure S2. Comparison of the amide NH regions of the 500 MHz ^{1}H NMR spectra of samples of Novo29 in DMSO-d_6. Samples of natural Novo29 were prepared gravimetrically at nominal concentrations of 1, 2, and 3 mM. The chemical shifts of the NH resonances vary strongly with concentration. The ^{1}H NMR spectrum of a sample of synthetic Novo29 prepared gravimetrically at a nominal concentration of 2 mM matches that of the 3 mM nominal concentration sample of natural Novo29. All samples were prepared by dissolving 1–2 mg of peptide in DMSO-d_6. Discrepancies in concentration reflect errors associated with weighing out small quantities of peptide.

Table S1. Chemical shifts of the amide NH resonances of samples of Novo29.

#	natural 1 mM (nominal)	natural 2 mM (nominal)	natural 3 mM (nominal)	synthetic 2 mM (nominal)
1	8.57	8.57	8.57	8.56
2	8.28	8.29	8.31	8.30
3	8.27	8.26	8.24	8.24
4-6	8.13	8.14	8.15	8.14
7/8	8.08 & 7.98	8.04 & 7.96	8.01 & 7.95	8.01 & 7.94
9	7.99	7.96	7.94	7.94
10	7.90	7.83	7.78	7.78
11-13	7.61	7.64	7.65	7.65
14	7.71	7.65	7.60	7.60
15	7.37	7.38	7.39	7.39
16/17	7.33	7.33	7.33	7.33
18-20	7.27	7.27	7.27	7.27

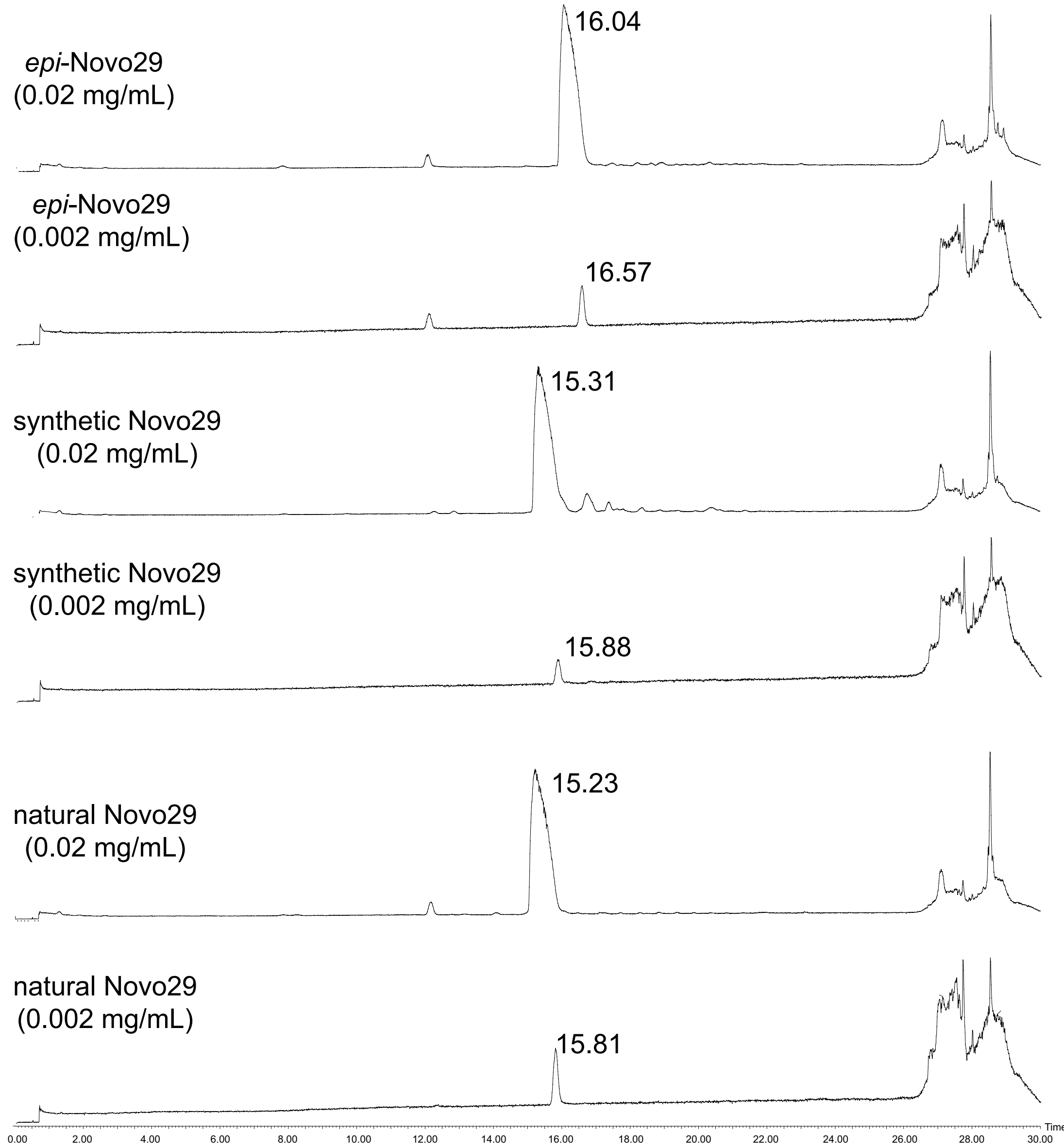

Figure S3. LC-MS of natural Novo29, synthetic Novo29, and epi-Novo29. Samples were run at (0.02 mg/mL) and (0.002 mg/mL). Traces are the total ion current (TIC) chromatograms of the samples run on a C4 column with a 3–27% CH_3CN over 25 minutes, followed by a 90% CH_3CN wash over two minutes, and a 3% CH_3CN 97% H_2O wash over one minute. The retention time and peak shape of Novo29 proved highly concentration dependent, with broad peaks and shorter elution times resulting at higher concentrations, again suggesting strong self-association.

LC/MS analysis was performed of the intact peptide sample (ACQUITY UPLC H-class system, Xevo G2-XS QTof, Waters corporation). The C4 column that was used was ACQUITY UPLC Protein BEH C4, 300 Å, 1.7 µm, 2.1 mm x 50 mm, Waters corp. The Xevo Z-spray source was operated in positive MS resolution mode, 400-4000da, with a capillary voltage of 3000V and a cone voltage of 40V (NaCsI calibration, Leu-enkephalin lock-mass). Nitrogen was used as the desolvation gas at 350C and a total flow of 800 L h–1. Retention times are given in minutes (min).

Table S2. Crystallographic properties, crystallization conditions, and data collection and model refinement statistics for *epi*-Novo29 (PDB ID 8CUG).

peptide	*epi*-Novo29 (synchrotron structure)
PDB ID	8CUG
space group	$P2_12_12_1$
a, *b*, *c* (Å)	12.105, 31.898, 37.101
α, β, λ (°)	90, 90, 90
peptides per asymmetric unit	2
crystallization conditions	2.8 M sodium acetate trihydrate pH 6.6
wavelength (Å)	1.00
resolution (Å)	24.19–1.13 (24.19–1.131)
total reflections	58890 (1401)
unique reflections	5590 (398)
multiplicity	10.5 (3.5)
completeness (%)	96.44 (69.54)
mean I/σ	22.28 (4.20)
Wilson B factor	8.32
R_{merge}	0.07324 (0.3204)
$R_{measure}$	0.07694 (0.3701)
$CC_{1/2}$	0.998 (0.938)
CC^*	0.999 (0.984)
R_{work}	0.1308 (0.2378)
R_{free}	0.1450 (0.2242)
number of non-hydrogen atoms	174
RMS_{bonds}	0.012
RMS_{angles}	1.63
Ramachandran favored (%)	100
outliers (%)	0
clashscore	3.77
average B-factor	13.49
ligands/ions	1
water molecules	42

Table S3. Crystallographic properties, crystallization conditions, and data collection and model refinement statistics for *epi*-Novo29 (PDB ID 8CUF).

peptide	*epi*-Novo29 (X-ray diffractometer structure with KI)
PDB ID	8CUF
space group	$P2_12_12_1$
a, *b*, *c* (Å)	12.1027, 31.5547, 36.9613
α, β, λ (°)	90, 90, 90
peptides per asymmetric unit	2
crystallization conditions	2.8 M sodium acetate trihydrate pH 6.6
wavelength (Å)	1.54
resolution (Å)	15.95–1.68 (1.74–1.68)
total reflections	22879 (199)
unique reflections	1741 (111)
multiplicity	13.1 (1.8)
completeness (%)	95.61 (63.07)
mean I/σ	36.10 (5.16)
Wilson B factor	8.93
R_{merge}	0.2386 (0.2593)
$R_{measure}$	0.2465 (0.3302)
$CC_{1/2}$	0.869 (0.822)
CC^*	0.964 (0.95)
R_{work}	0.1509 (0.2198)
R_{free}	0.1672 (0.0923)
number of non-hydrogen atoms	152
RMS_{bonds}	0.008
RMS_{angles}	1.08
Ramachandran favored (%)	100
outliers (%)	0
clashscore	3.75
average B-factor	9.85
ligands/ions	3
water molecules	18

Materials and Methods

General information

Chemicals and Supplies. All chemicals were used as received unless otherwise noted. Dry methylene chloride (CH_2Cl_2), tetrahydrofuran (THF), methanol (MeOH), and *N*,*N*-dimethylformamide (DMF) were obtained by passing through alumina under argon prior to use. 1,4-Dioxane (dioxane) was used without added stabilizers. Anhydrous, amine-free *N*,*N*-dimethylformamide (DMF), DIPEA, 2,4,6-collidine, and piperidine were purchased Alfa Aesar. HPLC grade acetonitrile and deionized water (18 MΩ), each containing 0.1% trifluoroacetic acid (TFA), were used for analytical and preparative reverse-phase HPLC, as well as reverse-phase chromatography using a Biotage® Isolera One flash column chromatography instrument. Commercial agents were used without purification, unless otherwise stated. (-)-Diethyl-D-tartrate was purchased from Chem-Impex. (+)-Diethyl L-tartrate, thionyl chloride ($SOCl_2$), and palladium activated on carbon (Pd/C, 10% Pd, 50 % wet with water) were purchased from Sigma Aldrich. Sodium azide (NaN_3) and benzyl bromide (BnBr) were purchased from Alfa Aesar. Hydrogen (H_2) and ammonia (NH_3) were purchased from Praxair. Sodium periodate ($NaIO_4$) and hydrochloric acid (HCl) were purchased from Fisher Chemicals. Di-*tert*-butyl dicarbonate (Boc_2O) and Fmoc *N*-hydroxysuccinimide ester (Fmoc-OSu) were purchased from GL Biochem. Ruthenium (III) chloride trihydrate ($RuCl_3 \cdot 3H_2O$) was purchased from Chem Scene. Lithium bromide (LiBr) was purchased from TCI. Amino acids, coupling agents, 2-chlorotrityl chloride resin, DIC, and triisopropylsilane were purchased from Chem-Impex.

Instrumentation. Bacteria were incubated in a Thermo Fisher Scientific MaxQ Shaker 6000. NMR spectra were recorded on a Bruker AVANCE 500 or AVANCE 600 spectrometers equipped with cryoprobes and were calibrated using residual solvent peaks ($CDCl_3$: δ_H = 7.26 ppm, δ_C = 77.16 ppm, DMSO-*d*6: δ_H = 2.50 ppm, δ_C = 39.51 ppm, and CD_3OD: δ_H = 3.31 ppm, δ_C =

49.15 ppm). Structural assignments of major and minor rotamers were made with additional EXSY information from gNOESY experiments. Assignments for natural Novo29, synthetic Novo29, and epi-Novo29 were made with additional information from gTOCSY and gNOESY experiments. Analytical reverse-phase HPLC was performed on Agilent 1260 Infinity II instrument equipped with a Phenomonex bioZen PEPTIDE 2.6 um XB-C18 column (150x4.6 mm), eluting with a gradient of acetonitrile and water (each containing 0.1% TFA) from 5-100% over 20 minutes. Peptides were purified on a Biotage Isolera One flash column chromatography instrument with a Biotage® SfarBio C18 D – Duo 300 Å 20 µm 10 g column. *epi*-Novo29 was then further purified by preparative reverse-phase HPLC on a Rainin Dynamax equipped with an Agilent Zorbax 250x 21.2 mm SB-C18 column. Both peptides were prepared and used as the trifluoroacetate (TFA) salts and were assumed to have one trifluoroacetic acid molecule per amine group on each peptide (two TFA molecules per peptide). Natural Novo29 was provided by NovoBiotic Pharmaceuticals LLC as the trifluoroacetate (TFA) salt. HRMS measurements were performed on a Waters LCT Premier with a TOF analyzer.

Chemical synthesis of Fmoc-(2*R*,3*R*)-hydroxyAsn-OH.[1-5]

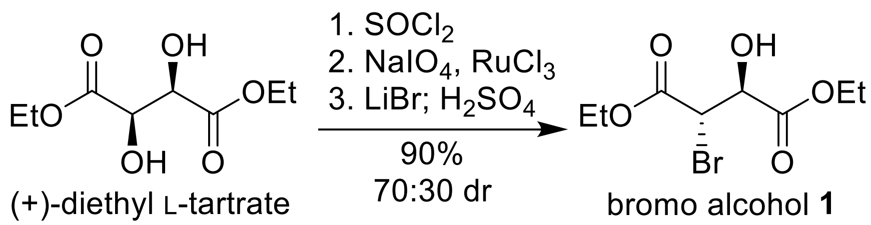

Bromo alcohol 1. A 500-mL, single-necked, round-bottom flask equipped with a magnetic stirring bar and a condenser fitted with a nitrogen inlet adapter was charged with a solution of (+)-diethyl L-tartrate (8.56 mL, 50.00 mmol, 1.0 equiv.) in 250 mL of CH_2Cl_2. (The reaction was run

at ca. 0.2 M concentration of the diethyl L-tartrate.) To the solution, $SOCl_2$ (7.25 mL, 100.00 mmol, 2.0 equiv.) was added in drops over ca. 5 minutes, followed by anhydrous DMF as a catalyst (0.25 mL, 3.20 mmol, 6 mol %) The reaction mixture was heated to 35 °C and stirred for 2 hours. Then, the solution was allowed to cool to room temperature, and concentrated to dryness by rotary evaporation to afford a cyclic sulfite intermediate as a yellow oil (12.61 g, 99% yield of the unpurified product). The cyclic sulfite was used without further purification.

The cyclic sulfite intermediate (12.61 g, 50.00 mmol, 1.0 equiv.) was dissolved in 250 mL CH_2Cl_2, 125 mL CH_3CN, and 62.5 mL H_2O, and the mixture was transferred to a 500-mL, single-necked, round-bottom flask equipped with a magnetic stirring bar and a nitrogen inlet adapter. $RuCl_3$•$3H_2O$ (0.327 g, 1.25 mmol, 2.5 mol %) was dissolved in 62.5 mL H_2O and added, followed by $NaIO_4$ (21.39 g, 100 mmol, 2 equiv.). The reaction mixture was stirred at 25 °C for 16 hours under N_2, and then diluted with 500 mL of Et_2O and 100 mL of H_2O. The organic layer was washed with 400 mL of saturated aqueous $NaHCO_3$, followed by 400 mL of saturated aqueous NaCl. The organic layer was concentrated to dryness by rotary evaporation to afford a cyclic sulfate intermediate as a yellow oil (12.39 g, 92 % yield of the unpurified product). The cyclic sulfate intermediate was used without further purification.

A 500-mL, three-necked, round-bottom flask equipped with a magnetic stirring bar and fitted with a nitrogen inlet adapter and two rubber septa was charged with LiBr (12.04 g, 138.60 mmol, 3 equiv.). The flask was placed under vacuum and heated to 120 °C in an oil bath for 1 hour to ensure LiBr was dry. The flask was then cooled to room temperature. The cyclic sulfate (12.39 g, 46.20 mmol, 1 equiv.) dissolved in 460 mL anhydrous THF was transferred to the flask with a capillary. (The reaction was run at ca. 0.1 M concentration of the cyclic sulfate.) The reaction mixture was stirred at 25 °C for 2 hours in a nitrogen atmosphere. The solution was concentrated

to dryness by rotary evaporation. The solution was dissolved in ca. 720 mL Et_2O and 480 mL aqueous 20% H_2SO_4. The solution was stirred for 18 hours at 25 °C. The organic and aqueous layers were separated, and the aqueous layer was extracted twice with 200 mL Et_2O and dried over Na_2SO_4. The solution was concentrated to dryness by rotary evaporation to afford bromo alcohol **1** as a yellow oil (12.43 g, 99 % yield of the unpurified product). Bromo alcohol **1** was generated as a mixture of diastereomers and used without further purification. ^{1}H NMR (500 MHz, $CDCl_3$): δ 4.81–4.65 (m, 2 H), 4.42–4.14 (m, 4 H), 1.40–1.23 (m, 6 H), 1.28 (q, J = 7.2 Hz, 1 H). [Note: Approximately 5 % (by integration) of an unknown impurity is present in this sample.] $^{13}C\{^1H\}$ NMR (125.7 MHz, $CDCl_3$): δ 170.5, 166.8, 72.7, 71.4, 63.0, 59.9, 49.7, 47.9, 14.2. HRMS: *m/z*: $[M + Na]^+$ calcd for $C_8H_{13}BrO_5Na^+$ 290.9844, observed 290.9844.

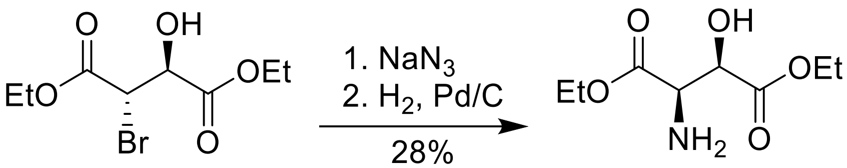

bromo alcohol **1**
amino alcohol **2**

Amino alcohol 2. A 250-mL, single-necked, round-bottom flask equipped with a magnetic stirring bar, a nitrogen inlet adapter, was charged with a solution of bromo alcohol **1** (12.43 g, 46.20 mmol, 1.0 equiv.) in 180 mL of anhydrous DMF. (The reaction was run at ca. 0.25 M concentration of the bromo alcohol **1**.) NaN_3 (9.01g, 138.60 mmol, 3 equiv.) was added to the solution, and the suspension was stirred for 21 h at 25 °C. The suspension was transferred to a separatory funnel and the round bottom flask was rinsed with 300 mL H_2O. The layers were separated, and the aqueous layer was extracted with EtOAc (3 x 200 mL). The combined organic layer was dried over Na_2SO_4, filtered, and dried using rotary evaporation to yield the azide

intermediate as a yellow oil (9.01 g, 84 % yield of the unpurified product). The azide intermediate was used without further purification.

A 500-mL, three-necked, round-bottom flask equipped with a magnetic stirring bar, a nitrogen inlet adapter, an inlet adapter fitted with a hydrogen balloon, and a rubber septum was charged with a solution of the crude azide intermediate (9.01 g, 38.97 mmol, 1.0 equiv.) in 100 mL of CH_3OH. (The reaction was run at 0.3 M concentration of the azide intermediate.) The flask was evacuated and back filled with nitrogen, and 10 % Pd/C (50% wet solid, 4.51 g, one half the mass of the azide intermediate) was added. A balloon containing H_2 gas was fitted to an inlet adapter and the system was evacuated and back filled with H_2 gas. The resulting suspension was stirred for 6 h, filtered through Celite, and washed with 200 mL of additional CH_3OH. The resulting solution was concentrated by rotary evaporation to and purified by column chromatography on silica gel (elution with 5:95 CH_3OH:CH_2Cl_2) to afford amino alcohol **2** as a yellow oil (2.69 g, 33 % yield). ^{1}H NMR (500 MHz, $CDCl_3$): δ 4.48 (d, *J* = 2.7 Hz, 1 H), 4.22–4.05 (m, 4 H), δ 3.75 (d, *J* = 2.8 Hz, 1 H) 2.53 (broad s, 3 H), 1.18 (q, *J* = 7.3 Hz, 6 H). [Note: Approximately 6 % (by integration) of an unknown impurity is present in this sample.] $^{13}C\{^1H\}$ NMR (125.7 MHz, $CDCl_3$): δ 172.6, 172.4, 72.0, 61.8, 61.4, 56.7, 13.96, 13.94. HRMS: *m/z*: $[M + Na]^+$ calcd for $C_8H_{15}NO_5Na^+$ 228.0848, observed 228.0843.

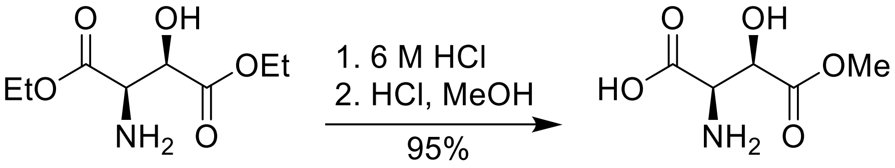

amino alcohol **2**

methyl ester **3**
(as the HCl salt)

Methyl ester 3. A 250-mL, single-necked, round-bottom flask equipped with a magnetic stirring bar and a condenser fitted with a nitrogen inlet adapter was charged with a solution of amino alcohol **2** (2.69 g, 13.11 mmol, 1.0 equiv.) in 65 ml of 6 M HCl. (The reaction was run at ca. 0.2 M concentration of the amino alcohol **2**.) The mixture was heated in an oil bath and stirred for 16 h at 100 °C, cooled to room temperature, and concentrated to dryness by rotary evaporation to afford a beige solid (2.59 g, 99 % yield of the unpurified product). The crude carboxylic acid intermediate was used in the next step without purification.

A 250-mL, single-necked, round-bottom flask equipped with a magnetic stirring bar and a condenser fitted with a nitrogen inlet adapter was charged with a solution of the crude carboxylic acid intermediate (2.45 g, 13.11 mmol, 1.0 equiv.) in 65 mL of CH_3OH. (The reaction was run at ca. 0.2 M concentration of the crude intermediate.) The mixture was cooled to 0 °C using an ice bath. A solution of 12 M HCl (2.0 mL) was then added dropwise to the flask over a few minutes. The flask was then heated in a preheated water bath at 65 °C for 30 minutes. The mixture was let to cool to room temperature and concentrated to dryness by rotary evaporation to afford methyl ester **3** as a beige foam (2.59 g, 96 % yield of the unpurified product). Methyl ester **3** was used without further purification. ^{1}H NMR (500 MHz, CD_3OD): δ 4.65 (d, *J* = 3.0 Hz, 1 H), 4.16 (d, *J* = 3.2, 1 H), 3.71 (s, 3 H). ^{13}C{^{1}H} NMR (125.7 MHz, CD_3OD) (major product, methyl ester **3**): δ 171.0, 168.8, 69.2, 55.2, 52.9; (minor impurity; diacid precursor impurity and dimethyl ester, partial data): δ 170.6, 167.9, 69.1, 55.3, 52.9. HRMS: *m/z*: $[M + Na]^+$ calcd for $C_5H_9NO_5Na^+$ 164.0559, observed 164.0556.

NOTE: We have found that timing is essential to the selective formation of the monomethyl ester (methyl ester **3**) from the corresponding diacid precursor. When we ran this reaction for two hours at 65 °C we observed the predominant formation of the undesired the dimethyl ester product.

When we ran this reaction for 15 minutes at 65 °C we observed ca. 50% conversion to the desired methyl ester **3**, with ca. 44% diacid precursor remaining, and ca. 6% of the dimethyl ester. When we ran this reaction for 30 minutes at 65 °C we observed mostly the desired methyl ester **3** (ca. 83%), with some remaining diacid precursor (ca. 12%), and some dimethyl ester (ca. 5%).

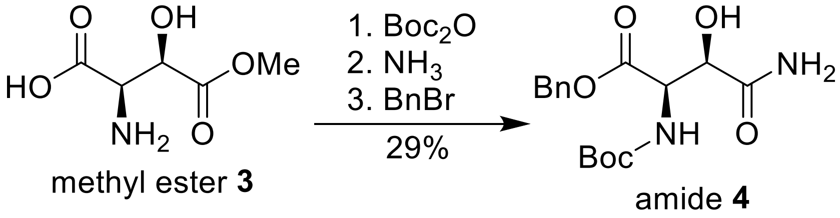

Amide 4. A 250-mL, single-necked, round-bottom flask equipped with a magnetic stirring bar and fitted with a nitrogen inlet adapter was charged with a solution of methyl ester **3** (containing the diacid precursor and dimethyl ester impurities) (2.59 g, 13.03 mmol, 1.0 equiv.) in 65 mL of 10 % w/v Na_2CO_3. The solution was cooled to 0 °C using an ice bath. While on ice, a separate solution of Boc_2O (8.53 g, 39.09 mmol, 3.0 equiv.) dissolved in 65 mL dioxane was added dropwise over 2–3 minutes. (The reaction was run at ca. 0.15 M concentration of the methyl ester **3**.) The mixture was brought to room temperature, stirred for 20 h, and concentrated to dryness using rotary evaporation. The resulting residue was dissolved in 250 mL EtOAc and transferred to a separatory funnel. The mixture was washed with 1 M HCl (3 x 250 mL). The organic layer was dried over Na2SO4 and concentrated to a yellow oil. The crude product was purified by column chromatography on silica gel (elution with 10:90 CH_3OH:$CHCl_3$) to afford the Boc-protected intermediate as an oil (1.43 g, 42% yield).

A 125-mL, single-necked, high-pressure flask equipped with a magnetic stirring bar was charged with a solution of the Boc-protected intermediate (1.43 g, 5.43 mmol, 1.0 equiv.) in ca. 18 mL CH_3OH. (The reaction was run at ca. 0.3 M concentration of the Boc-protected intermediate.) A flow of NH_3 gas was applied through a syringe directly into the solution and

bubbled for 20–30 min to ensure saturation with NH_3. The vessel was then capped, and the solution was stirred for 72 h at 25 °C. The solution was concentrated to dryness using rotary evaporation to afford the amide intermediate as a beige solid (1.21 g, 94% yield of the unpurified product). The crude intermediate was used in the next step without further purification.

A 100-mL, single-necked, round-bottom flask equipped with a magnetic stirring bar and fitted with a nitrogen inlet adapter was charged with a solution of the amide intermediate (1.21 g, 11.9 mmol, 1.0 equiv.) in ca. 25 mL of anhydrous DMF. (The reaction was run at ca. 0.2 M concentration of the amide intermediate.) The solution was cooled to 0 °C using an ice bath, and $NaHCO_3$ (1.07 g, 12.70 mmol, 2.5 equiv.) followed by benzyl bromide (2.42 mL, 20.4 mmol, 4.0 equiv.) was added dropwise over 10 minutes. The mixture was stirred for 2 hours at 0 °C, warmed to room temperature, and stirred for an additional 24 hours under N_2. The resulting solution was cooled again to 0 °C using an ice bath, 75 mL of H_2O was added to quench the reaction. The solution was transferred to a separatory funnel and extracted with EtOAc (3 x 50 mL). The combined organic layer was washed with 50 mL saturated aqueous NaCl, dried with Na_2SO_4, and concentrated to using rotary evaporation. The crude product was then purified by column chromatography on silica gel (elution with 5:95 CH_3OH:CH_3Cl) to afford amide **4** as white solid (1.27 g, 73% yield). ^{1}H NMR (500 MHz, $CDCl_3$): δ 7.31–7.36 (m, 5 H), 6.85 (br s, 1 H), 6.04 (br s, 1 H), 5.62 (d, J = 9.1 Hz, 1 H), 5.19 (s, 2 H), 5.17–5.30 (m, 2 H), 4.75 (br d, J = 9.1 Hz, 1 H), 4.61 (br s, 1 H), 1.39 (s, 9 H). [Note: Approximately 10 % (by integration) of an unknown impurity is present in this sample.] $^{13}C\{^1H\}$ NMR (125.7 MHz, $CDCl_3$): δ 173.7, 172.1, 170.2, 156.6, 135.1, 128.7, 128.5, 128.3, 81.6, 80.9, 74.8, 72.2, 68.1, 67.7, 56.4, 28.2. HRMS: *m/z*: $[M + Na]^+$ calcd for $C_{16}H_{22}N_2O_6Na^+$ 361.1375, observed 361.1371.

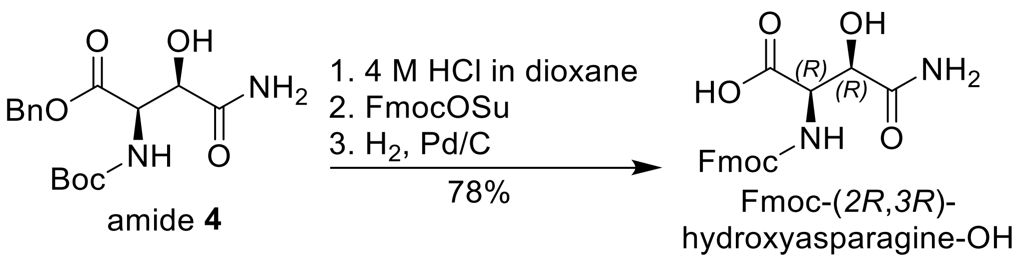

Fmoc-(2*R*,3*R*)-hydroxyasparagine-OH. A 250-mL, single-necked, round-bottom flask equipped with a magnetic stirring bar and fitted with a nitrogen inlet adapter was charged with a solution of amide **4** (1.27 g, 3.70 mmol, 1.0 equiv.) in ca. 40 mL of 4 N HCl in dioxane. (The reaction was run at ca. 0.1 M concentration of the amide **4**.) The solution was stirred for 2 hours at 25 °C and concentrated to dryness by rotary evaporation, to afford the amine intermediate as a pale-yellow solid (0.96 g, 95% yield of the unpurified product). The crude intermediate was used in the next step without further purification.

The crude amine intermediate (0.96 g, 3.50 mmol, 1.0 equiv.) was dissolved in 52 mL of 1:1 dioxane:water. (The reaction was run at ca. 0.06 M concentration of the crude amine intermediate.) The solution was cooled to 0 °C using an ice bath, and $NaHCO_3$ was added until the solution reached a pH of 6.8. Fmoc-OSu (1.42 g, 4.20 mmol, 1.2 equiv.) was then added, and the reaction mixture was stirred for 1 h on ice, warmed to room temperature, and stirred for an additional 20 hours under N_2. The resulting suspension was diluted with 120 mL EtOAc and 180 mL of saturated $NaHCO_3$ and stirred for ca. 5 min at 25 °C. The solution was transferred to a separatory funnel and the organic layer was collected; the aqueous layer was then extracted with EtOAc (2 x 80 mL). The combined organic layer was then dried with Na_2SO_4 and concentrated to by rotary evaporation to a white solid (1.61 g, 99% yield). A sample of the crude product was then purified by column chromatography on silica gel (elution with 5:95 CH_3OH:CH_3Cl) to afford the Fmoc-protected intermediate as a white solid that was used in the subsequent reaction.

A 250-mL, three-necked, round-bottom flask equipped with a magnetic stirring bar, a nitrogen inlet adapter, an inlet adapter fitted with a hydrogen balloon, and a rubber septum was charged with a solution of the Fmoc-protected intermediate (0.21 g, 0.46 mmol, 1 equiv.) from the previous step in ca. 5 mL of CH_3OH. (The reaction was run at 0.05 M concentration of the Fmoc-protected intermediate.) The flask was evacuated and back filled with nitrogen, and 10 % Pd/C (50% wet solid, 0.07 g, one half the mass of Fmoc-protected intermediate) was added. A balloon containing H_2 gas was fitted to an inlet adapter and the system was evacuated and back filled with H_2 gas. The resulting suspension was stirred for 15 minutes, filtered through Celite, and washed with additional CH_3OH. The resulting solution was concentrated by rotary evaporation to afford Fmoc-(2*R*,3*R*)-hydroxyasparagine-OH as an off-white solid (0.14 g, 83% yield of the unpurified product). ^{1}H NMR (500 MHz, CD_3SOCD_3): δ 7.89 (d, *J* = 7.7 Hz, 2 H), 7.73 (t, *J* = 8.9 Hz, 2 H), 7.47–7.26 (m, 7 H), 6.97 (br s, 1 H), 4.43 (d, *J* = 8.9 Hz, 1 H), 4.37 (s, 1 H), 4.29–4.17 (m, 3 H); (minor rotamer, ca. 15%, partial data): δ 7.64 (d, *J* = 6.8 Hz, Fmoc aromatic CH), 6.39 (d, *J* = 8.1 Hz, carbamate NH), 4.59 (d, *J* = 8.3 Hz, hydroxyAsn CH), 4.41 (s, hydroxyAsn CH), 4.19–4.06 (m, hydroxyAsn CH). ^{13}C{^{1}H} NMR (125.7 MHz, CD_3SOCD_3): δ 173.2, 171.9, 156.1, 143.8, 143.7, 140.7, 127.7, 127.2, 127.1, 125.4, 125.4, 120.1, 71.4, 65.9, 56.9, 46.5. HRMS: *m/z*: $[M + Na]^+$ calcd for $C_{19}H_{18}N_2O_6Na^+$ 393.1063, observed 393.1060.

Chemical synthesis of Fmoc-(2*R*,3*S*)-hydroxyAsn.

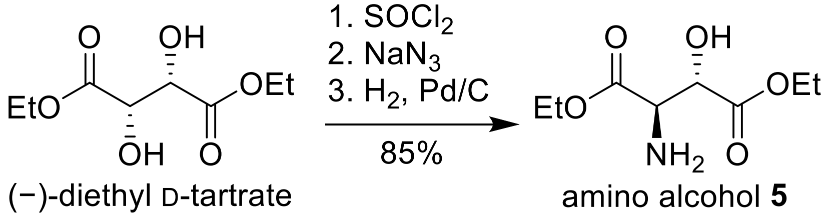

Amino alcohol 5. A 500-mL, single-necked, round-bottom flask equipped with a magnetic stirring bar and a condenser fitted with a nitrogen inlet adapter was charged with a solution of (-)-diethyl-D-tartrate (8.56 mL, 50.00 mmol, 1.0 equiv.) in 250 mL of CH_2Cl_2 (the reaction was run at ca. 0.2 M concentration of the diethyl-D-tartrate). To the solution, $SOCl_2$ (7.25 mL, 100.00 mmol, 2.0 equiv.) was added dropwise, followed by 0.25 mL cat. anhydrous DMF. The reaction mixture was heated to 35 °C and stirred for 2 hours. Then, the solution was cooled to room temperature, and concentrated to dryness by rotary evaporation to afford a cyclic sulfite intermediate as a yellow oil (12.41 g, 98% yield of the unpurified product). The cyclic sulfite was used without further purification.

A 250-mL, single-necked, round-bottom flask equipped with a magnetic stirring bar, a nitrogen inlet adapter, was charged with a solution of the cyclic sulfite intermediate (12.41 g, 49.20 mmol, 1.0 equiv.) in 100 mL of anhydrous DMF (the reaction was run at 0.5 M concentration of the bromo alcohol **1**). NaN_3 (3.99 g, 61.50 mmol, 1.25 equiv.) was added to the solution, and the suspension was stirred for 21 h at 25 °C. The suspension was transferred to a separatory funnel and the round bottom flask was rinsed with 300 mL H_2O. The layers were separated and the aqueous layer was extracted with EtOAc (3 x 200 mL). The combined organic layer was dried over Na_2SO_4, filtered, and dried using rotary evaporation to yield the azide intermediate as a yellow oil (9.96 g, 88% yield of the unpurified product). The azide intermediate was used without further purification.

A 500-mL, three-necked, round-bottom flask equipped with a magnetic stirring bar, a nitrogen inlet adapter, an inlet adapter fitted with a hydrogen balloon, and a rubber septum was charged with a solution of the crude azide intermediate (9.96 g, 43.10 mmol, 1.0 equiv.) in 140 mL of CH_3OH (the reaction was run at 0.2 M concentration of the azide intermediate). The flask was evacuated and back filled with nitrogen, and 10 % Pd/C (50% wet solid, 4.98 g, one half the mass of the azide intermediate) was added. A balloon containing H_2 gas was fitted to an inlet adapter and the system was evacuated and back filled with H_2 gas. The resulting suspension was stirred for 6 h, filtered through Celite, and washed with 200 mL of additional CH_3OH. The resulting solution was concentrated by rotary evaporation to afford amino alcohol **5** as a yellow oil (8.84 g, 99% yield of the unpurified product). Amino alcohol **5** was used without further purification. An analytical sample was purified by column chromatography on silica gel (elution with 5:95 CH_3OH:CH_2Cl_2) for analysis. ^{1}H NMR (500 MHz, $CDCl_3$): δ 4.34 (d, J = 3.3 Hz, 1 H), 4.10–3.95 (m, 4 H), 3.75 (d, J = 3.3 Hz, 1 H) 3.75 (broad s, 3 H), 1.18 (m, 6 H). $^{13}C\{^1H\}$ NMR (125.7 MHz, $CDCl_3$): δ 171.86, 171.82, 72.8, 61.5, 61.2, 57.5, 13.95, 13.94. HRMS: *m/z*: $[M + Na]^+$ calcd for $C_8H_{15}NO_5Na^+$ 228.0848, observed 228.0843.

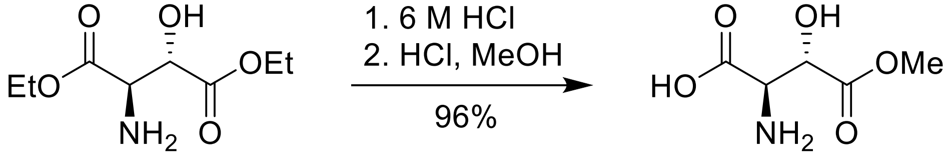

amino alcohol **5**

methyl ester **6**
(as the HCl salt)

Methyl ester 6. A 250-mL, single-necked, round-bottom flask equipped with a magnetic stirring bar and a condenser fitted with a nitrogen inlet adapter was charged with a solution of amino alcohol **5** (8.84 g, 43.10 mmol, 1.0 equiv.) in 200 mL of 6 M HCl (the reaction was run at

ca. 0.2 M concentration of the amino alcohol **2**). The mixture was heated in an oil bath and stirred for 16 h at 100 °C, cooled to room temperature, and concentrated to dryness by rotary evaporation to afford a beige solid (7.99 g, 98% yield of the unpurified product). The crude carboxylic acid intermediate was used in the next step without purification.

A 250-mL, single-necked, round-bottom flask equipped with a magnetic stirring bar and a condenser fitted with a nitrogen inlet adapter was charged with a solution of the crude carboxylic acid intermediate (7.99, 43.10 mmol, 1.0 equiv.) in 240 mL of CH_3OH (the reaction was run at ca. 0.2 M concentration of the crude intermediate). The mixture was cooled to 0 °C using an ice bath. A solution of 12 M HCl (8 mL) was then added dropwise to the flask over 10 minutes. The flask was then heated in a preheated water bath at 65 °C for 15 minutes. The mixture was cooled to room temperature and concentrated to dryness by rotary evaporation to afford methyl ester **6** as a beige foam (8.60 g, 98% yield of the unpurified product). Methyl ester **6** was used without further purification. ^{1}H NMR (500 MHz, CD_3OD): (major product, methyl ester **6**, ca. 50% by integration): δ 8.65 (br s, 1 H), 4.67 (d, *J* = 2.6 Hz, 1 H), 4.46 (d, *J* = 2.6, 1 H), 3.80 (s, 3 H); (diacid precursor impurity, ca. 44% by integration): δ 4.61 (d, *J* = 2.5 Hz, 1 H), 4.45 (d, *J* = 2.5 Hz, 1 H). ^{13}C{^{1}H} NMR (125.7 MHz, CD_3OD) (major product, methyl ester **6**): δ 170.5, 166.9, 69.9, 55.3, 51.8; (diacid precursor impurity): δ 171.5, 167.1, 68.8, 55.4. HRMS: *m/z*: $[M + H]^+$ calcd for $C_5H_9NO_5H^+$ 164.0559, observed 164.0558.

NOTE: Running the reaction for 15 minutes minimizes the formation of the dimethyl ester while still allowing a satisfactory yield of the desired monomethyl ester. Under these conditions, there is still a significant amount of the residual diacid precursor, which is observed in the ^{1}H NMR and ^{13}C NMR spectra of the unpurified product. Based on our observation for the preparation

monomethyl ester **3** (above) we anticipate that 30 minutes of heating time would lead to a better yield of the desired methyl ester **6**.

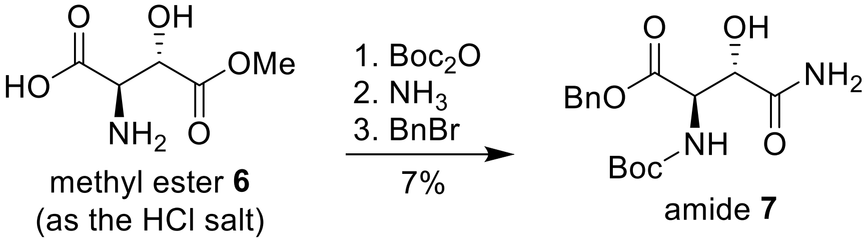

Amide 7. A 250-mL, single-necked, round-bottom flask equipped with a magnetic stirring bar and fitted with a nitrogen inlet adapter was charged with a solution of methyl ester **6** and the diacid precursor impurity (8.60 g, 43.10 mmol, 1.0 equiv.) in 140 mL of 10 % w/v Na_2CO_3. The solution was cooled to 0 °C using an ice bath. While on ice, a separate solution of Boc_2O (7.51 g, 34.62 mmol, 3.0 equiv.) dissolved in 140 mL dioxane (to give a final concentration of 0.15 M for the methyl ester **6**) was slowly added over 2–3 minutes. The mixture was brought to room temperature, stirred for 19.5 h, and concentrated to dryness using rotary evaporation. The resulting residue was dissolved in 500 mL EtOAc and transferred to a separatory funnel. The mixture was washed with 1 M HCl (3 x 500 mL). The organic layer was dried over Na_2SO_4 and concentrated to a yellow oil. The crude product was purified by column chromatography on silica gel (elution with 10:90 CH_3OH:$CHCl_3$) to afford the Boc-protected intermediate as a yellow oil (1.65 g, 15% yield).

A 125-mL, single-necked, high-pressure flask equipped with a magnetic stirring bar was charged with a solution of the Boc-protected intermediate (1.65 g, 6.27 mmol, 1.0 equiv.) in ca. 20 mL CH_3OH (the reaction was run at ca. 0.3 M concentration of the Boc-protected intermediate). A flow of NH_3 gas was applied through a syringe directly into the solution and bubbled for 20–30 min to ensure saturation with NH_3. The vessel was then capped, and the solution was stirred for 72 h at 25 °C. The solution was concentrated to dryness using rotary evaporation to afford the

amide intermediate as a beige solid (1.46 g, 98% yield of the unpurified product). The crude intermediate was used in the next step without further purification.

A 100-mL, single-necked, round-bottom flask equipped with a magnetic stirring bar and fitted with a nitrogen inlet adapter was charged with a solution of the amide intermediate (1.46 g, 3.64 mmol, 1.0 equiv.) in ca. 31 mL of anhydrous DMF (the reaction was run at ca. 0.2 M concentration of the amide intermediate). The solution was cooled to 0 °C using an ice bath, and $NaHCO_3$ (0.76 g, 9.10 mmol, 2.5 equiv.) followed by benzyl bromide (1.73 mL, 14.56 mmol, 4.0 equiv.) was added dropwise over 10 minutes. The mixture was stirred for 2 hours at 0 °C, warmed to room temperature, and stirred for an additional 24 hours under N_2. The resulting solution was cooled again to 0 °C using an ice bath, 75 mL of H_2O was added to quench the reaction. The solution was transferred to a separatory funnel and extracted with EtOAc (3 x 50 mL). The combined organic layer was washed with 50 mL saturated aqueous NaCl, dried with Na_2SO_4, and concentrated to using rotary evaporation. The crude product was then purified by column chromatography on silica gel (elution with 5:95 CH_3OH:CH_3Cl) to afford amide **7** as white solid (1.00 g, 47% yield). ^{1}H NMR (500 MHz, $CDCl_3$): δ 7.42–7.31 (m, 5 H), 6.79 (broad s, 1 H), 5.82 (broad d, J = 4.8 Hz, 1 H), 5.62 (broad s, 1 H), 5.54 (broad d, J = 6.6 Hz, 1 H), 5.31–5.18 (m, 2 H), 4.72 (broad d, J = 5.1 Hz, 1 H), 4.65 (broad d, J = 4.5 Hz, 1 H), 1.45 (s, 9 H). ^{13}C{^{1}H} NMR (125.7 MHz, $CDCl_3$): δ 173.6, 168.1, 158.2, 135.1, 128.7, 128.7, 128.6, 81.7, 75.0, 68.2, 57.5, 28.3. HRMS: *m/z*: $[M + Na]^+$ calcd for $C_{16}H_{22}N_2O_6Na^+$ 361.1375, observed 361.1371.

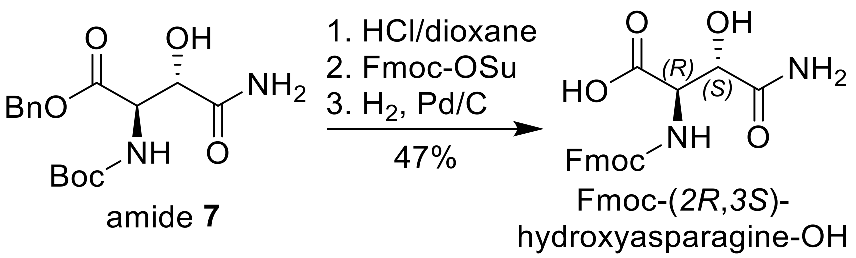

Fmoc-(2*R*,3*S*)-hydroxyasparagine-OH. A 250-mL, single-necked, round-bottom flask equipped with a magnetic stirring bar and fitted with a nitrogen inlet adapter was charged with a solution of the amide **7** (1.00 g, 2.96 mmol, 1.0 equiv.) in ca. 30 mL of 4 N HCl in dioxane. (The reaction was run at ca. 0.1 M concentration of the amide **7**.) The solution was stirred for 2 h and concentrated to dryness by rotary evaporation to a white solid (0.71 g, 99% yield of the unpurified product).

The crude intermediate (0.71 g, 2.98 mmol, 1 equiv.) was dissolved in 52 mL of 1:1 dioxane:water. (The reaction was run at ca. 0.06 M concentration of the crude intermediate). The solution was cooled to 0 °C using an ice bath, and $NaHCO_3$ was added until the solution reached a pH of 6.8. Fmoc-OSu (1.20 g, 3.55 mmol, 1.2 equiv.) was then subsequently added, and mixture was stirred for 1 h on ice, brought to room temperature, and stirred for an additional 18–20 h under N_2. The resulting suspension was diluted with 120 mL EtOAc and 180 mL of saturated $NaHCO_3$ and stirred for ca. 5 min. The solution was transferred to a separatory funnel and the organic layer was collected; the aqueous layer was then extracted with EtOAc (2 x 80 mL). The combined organic layer was then dried with Na_2SO_4 and concentrated by rotary evaporation to a white solid. The crude product was then purified by column chromatography on silica gel (elution with 5:95 CH_3OH:CH_3Cl) to afford the Fmoc-protected intermediate as a white solid (1.07 g, 79% yield).

A 250-mL, three-necked, round-bottom flask equipped with a magnetic stirring bar, a nitrogen inlet adapter, an inlet adapter fitted with a hydrogen balloon, and a rubber septum was

charged with a solution of the Fmoc-protected intermediate (1.07 g, 2.32 mmol, 1 equiv.) from the previous step in ca. 55 mL of anhydrous CH_3OH. (The reaction was run at 0.05 M concentration of the Fmoc-protected intermediate assuming 100 % conversion). The flask was evacuated and back filled with nitrogen, and ca. 10 % Pd/C (50% wet solid, one half the mass of the Fmoc-protected intermediate, 0.5 g) was added. A balloon containing H_2 gas was fitted to an inlet adapter and the system was evacuated and back filled with H_2 gas. The resulting suspension was stirred for 24 h, filtered through Celite, and washed with additional CH_3OH. The resulting solution was concentrated by rotary evaporation to afford Fmoc-(2*R*,3*S*)-hydroxyasparagine-OH as an off-white solid (0.51 g, 60% yield of the unpurified product). ^1H NMR (500 MHz, CD_3SOCD_3): δ 7.89 (d, J = 7.5 Hz, 2 H), 7.77–7.69 (m, 2 H), 7.47–7.28 (m, 7 H), 7.03 (d, J = 9.5 Hz, 1 H), 4.47 (dd, J = 9.5, 2.1 Hz, 1 H), 4.37 (d, J = 2.0 Hz, 1 H), 4.26–4.19 (m, 3 H); (minor rotamer, ca. 16%, partial data): δ 7.63 (d, J = 7.2 Hz, Fmoc aromatic CH), 6.44 (d, J = 9.5 Hz, carbamate NH), 4.64 (d, J = 9.4 Hz, hydroxyAsn CH), 4.42 (s, hydroxyAsn CH), 4.14–4.04 (m, hydroxyAsn CH). $^{13}C\{^1H\}$ NMR (125.7 MHz, CD_3SOCD_3): δ 173.2, 171.9, 156.1, 143.8, 143.7, 140.7, 127.7, 127.17, 127.13, 125.43, 125.36, 120.1, 71.4, 65.9, 56.9, 46.5. HRMS: *m/z*: $[M + Na]^+$ calcd for $C_{19}H_{18}N_2O_6Na^+$ 393.1063, observed 393.1073.

Synthesis of Novo29 and *epi*-Novo29 [6]

Peptide synthesis procedure. Novo29 and *epi*-Novo29 were synthesized by manual solid-phase peptide synthesis of the corresponding linear peptide on 2-chlorotrityl resin, followed by on-resin esterification, solution-phase cyclization, deprotection, and purification. A step-by-step procedure is detailed below.

a. Loading the resin. 2-Chlorotrityl chloride resin (300 mg, 1.07 mmol/g) was added to a Bio-Rad Poly-Prep chromatography column (10 mL). Dry CH_2Cl_2 (8 mL) was used to suspend and swell the resin for 30 min with gentle rocking. After the solution was drained from the resin, a separate solution of Fmoc-Leu-OH (75 mg, 0.7 equiv., 0.21 mmol) in 6% (v/v) 2,4,6-collidine in dry CH_2Cl_2 (8 mL) was added and the suspension was gently rocked for 5–6 h. The solution was then drained, and a mixture of CH_2Cl_2/ CH_3OH /*N*,*N*-diisopropylethylamine (DIPEA) (17:2:1, 8 mL) was added immediately. The resin was gently rocked for 1 h, to cap the unreacted 2-chlorotrityl chloride resin sites. The resin was then washed three times with dry CH_2Cl_2 and dried by passing nitrogen through the vessel. This procedure typically yields 0.15–0.20 mmol of loaded resin, as assessed by spectrophotometric analysis.

b. Manual peptide coupling. The loaded resin was suspended in dry DMF and then transferred to a solid-phase peptide synthesis vessel. Residues 6 through 1 were manually coupled using Fmoc-protected amino acid building blocks. The coupling cycle consisted of *i*. Fmoc-deprotection with of 20% (v/v) piperidine in DMF (5 mL) for 5–10 min at room temperature, *ii*. washing with dry DMF (4 x 5 mL), *iii*. coupling of the amino acid (4 equiv.) with HCTU (4 equiv.) in 20% (v/v) 2,4,6-collidine in dry DMF (5 mL) for 20–30 min, and *iv.* washing with dry DMF (4 x 5 mL). The last amino acid coupling of the linear sequence is Boc-Phe-OH, which intentionally protects the *N*-terminus from being reactive during the esterification step and cyclization steps. The resin was then transferred to a clean Bio-Rad PolyPrep chromatography column.

c. Esterification. In a test tube, Fmoc-Leu-OH (10 equiv.) and diisopropylcarbodiimide (10 equiv.) were dissolved in dry CH_2Cl_2 (5 mL). The resulting solution was filtered through a 0.20-μm nylon filter, and 4-dimethylaminopyridine (1 equiv.) was added to the filtrate. The resulting

solution was transferred to the resin and gently agitated for 1 h. The solution was then drained, and the resin was washed with dry CH_2Cl_2 (3 x 5 mL) and DMF (3 x 5 mL).

d. Fmoc deprotection of Leu$_8$. The Fmoc protecting group on Leu_8 was removed by adding 20% (v/v) piperidine in DMF for 30 min. The solution was drained, and the resin was washed with dry DMF (3 x 5 mL) and CH_2Cl_2 (3 x 5 mL).

e. Cleavage of the linear peptide from chlorotrityl resin. The linear peptide was cleaved from the resin by rocking the resin in a solution of 20% (v/v) 1,1,1,3,3,3-hexafluoroispropanol (HFIP) in CH_2Cl_2 (8 mL) for 1 h. The suspension was filtered, and the filtrate was collected in a 250-mL round-bottomed flask. The resin was washed with additional cleavage solution (8 mL) for 30 min and filtered into the same 250 mL round bottom-bottomed flask. The combined filtrates were concentrated by rotary evaporation and further dried by vacuum pump to afford the crude protected linear peptide, which was cyclized without further purification.

d. Cyclization of the linear peptide. The crude protected linear peptide was dissolved in dry DMF (125 mL). HOAt (6 equiv.) and HATU (6 equiv.) were dissolved in 8 mL of dry DMF in a test tube to which 300 µL of diisopropylethylamine was added and the solution mixed until homogenous. The solution was then added to the round-bottom flask containing the dissolved peptide and the mixture was stirred under nitrogen at room temperature for 16–20 h. The reaction mixture was concentrated by rotary evaporation and further dried by vacuum pump to afford the crude protected cyclized peptide, which was immediately subjected to global deprotection.

e. Global deprotection of the cyclic peptide. The protected cyclic peptides were dissolved in TFA:triisopropylsilane (TIPS):H_2O (18:1:1, 10 mL) in a 1000-mL round-bottomed flask equipped with a stir bar. The solution was stirred for 1 h under nitrogen. During the 1 h deprotection, two 50-mL conical tubes containing 40 mL of dry Et_2O each were chilled on ice.

After the 1 h deprotection, the peptide solution was split between the two conical tubes of Et_2O. The tubes were then centrifuged at 600x*g* for 10 min, decanted, and washed with fresh Et_2O. This process of decanting and washing was repeated for two more times. The pelleted peptides were dried under nitrogen for 15–20 min. The deprotected cyclic peptide was then purified by reverse-phase HPLC (RP-HPLC).

f. Reverse-phase HPLC purification. The peptide was dissolved in 20% CH_3CN in H_2O (5 mL) and pre-purified on a Biotage Isolera One flash chromatography instrument equipped with a Biotage® Sfär Bio C18 D - Duo 300 Å 20 µm 25 g column. The solution of crude cyclic peptide was injected at 20% CH_3CN and eluted with a gradient of 20–50% CH_3CN. After this purification step Novo29 precipitated out of solution. The fractions containing the pure peptide were lyophilized. For *epi*-Novo29 the fractions containing the desired peptide were concentrated by rotary evaporation, diluted in 20% CH_3CN, injected on a Rainin Dynamax instrument, and eluted over a gradient of 20–40% CH_3CN over 90 min. The collected fractions were analyzed by analytical HPLC and MALDI-TOF, and the pure fractions were concentrated by rotary evaporation and lyophilized. These procedures typically yielded 5 mgs (~1 % yield) of synthetic peptides (Novo29 or *epi*-Novo29) as the TFA salts.

NMR spectroscopic studies of natural Novo29, synthetic Novo29, and *epi*-Novo29[7]

Sample Preparation. NMR spectroscopic studies of natural Novo29 and synthetic peptides were performed in DMSO-d_6. The solutions were prepared gravimetrically by dissolving a weighed portion of the peptide in the appropriate volume of solvent.

TOCSY and NOESY Data Collection. NMR spectra were recorded on a Bruker 600 MHz spectrometer with a Bruker CBBFO helium-cooled cryoprobe. TOCSY spectra were recorded with

2048 points in the *f*2 dimension and either 512 increments in the *f*1 dimension with NS = 8 and a 150-ms spin-lock mixing time. NOESY spectra were recorded with 2048 points in the *f*2 dimension and 512 increments in the *f*1 dimension with NS = 8 and a 200-ms mixing time.

TOCSY and NOESY Data Processing. NMR spectra were processed with Bruker TopSpin software. Automatic baseline correction was applied in both dimensions after phasing the spectra. 2D TOCSY and NOESY spectra were Fourier transformed to a final matrix size of 1024 x 1024 real points using a Qsine weighting function and forward linear prediction in the *f*1 dimension.

MIC assays[8]

Preparing the peptide stocks. Solutions of natural Novo29, synthetic Novo29, and *epi*-Novo29 were prepared gravimetrically by dissolving an appropriate amount of peptide in an appropriate volume of sterile DMSO to make 1 mg/mL stock solutions. The stock solutions were stored at -20 °C for subsequent experiments.

Preparation and tray setup. Bacillus subtilis (ATCC 6051), *Staphylococcus epidermidis* (ATCC 14990), and *Escherichia coli* (ATCC 10798) were cultured from glycerol stocks in Mueller-Hinton broth overnight in a shaking incubator at 37 °C. An aliquot of the 1 mg/mL antibiotic stock solutions were diluted to make a 64 μg/mL solution with Mueller-Hinton broth. A 200-μL aliquot of the 64 μg/mL solution was transferred to a 96-well plate. Two-fold serial dilutions were made with media across a 96-well plate to achieve a final volume of 100 μL in each well. These solutions had the following concentrations: 64, 32, 16, 8, 4, 2, 1, 0.5, 0.25, 0.125, and 0.0625 μg/mL. The overnight cultures of each bacterium were diluted with Mueller-Hinton broth to an OD_{600} of 0.075 as measured for 200 μL in a 96-well plate. The diluted mixture was further diluted to 1×10^6 CFU/mL with the appropriate media. A 100-μL aliquot of the 1×10^6 CFU/mL

bacterial solution was added to each well in 96-well plates, resulting in final bacteria concentrations of 5 × 10^5 CFU/mL in each well. As 100 µL of bacteria were added to each well, natural Novo29, synthetic Novo29 and *epi*-Novo29 were also diluted to the following concentrations: 32, 16, 8, 4, 2, 1, 0.5, 0.25, 0.125, 0.0625, and 0.03125 µg/mL. The plate was covered with a lid and incubated at 37 °C for 16 h. The optical density measurements were recorded at 600 nm and were measured using a 96-well UV/vis plate reader (MultiSkan GO, Thermo Scientific). The MIC values were taken as the lowest concentration that had no bacteria growth. Each MIC assay was run in triplicate in three independent runs to ensure reproducibility.

X-ray crystallography of *epi*-Novo29[9]

Crystallization of epi-Novo29. The hanging-drop vapor-diffusion method was used to determine initial crystallization conditions for *epi*-Novo29. Each peptide was screened in 96-well plate format using three crystallization kits (Crystal Screen, Index, and PEG/ION) from Hampton Research. A TTP LabTech Mosquito nanondisperse was used to make three 150 nL hanging drops for each well condition. The three hanging drops differed in the ratio of peptide to well solution for each condition in the 96-well plate. A 10 mg/mL solution of *epi*-Novo29 peptide in deionized water was combined with a well solution in ratios of 1:1, 1:2, and 2:1 peptide:well solution at appropriate volumes to create the three 150 nL hanging drops. Crystals of *epi*-Novo29 grew in well conditions of 2.8 M sodium acetate at pH 7.0.

Crystallization conditions for *epi*-Novo29 were optimized using a 4 x 6 matrix Hampton 24-well plate. For the *epi*-Novo29, the pH of the buffer was varied in each row (6.5, 6.6, 6.8, and 7.0). The concentration of sodium acetate in each column was varied in increments of 0.2 M (2.2, 2.4, 2.6, 2.8, 3.0, 3.2). Three hanging-drops were prepared on borosilicate glass slides by combing a 10 mg/mL solution of *epi*-Novo29 in deionized water with the well solution in the following

amounts: 1 µL:1 µL, 2 µL:1 µL, and 1 µL:2 µL. Slides were inverted and pressed firmly against the silicone grease surrounding each well. Crystals were harvested with a nylon loop attached to a copper or steel pin, and flash frozen in liquid nitrogen prior to data collection. A single crystal was soaked in a mixture of potassium iodide (KI) and well solution to incorporate iodide heavy atoms into the lattice. A higher resolution data set was subsequently collected from crystals grown in similar conditions and collected on a synchrotron X-ray source. The final optimized crystallization condition for *epi*-Novo29 is summarized in Tables S2 and S3.

Data collection, data processing, and structure determination. X-ray diffraction data from a single *epi*-Novo29 crystal soaked in a mixture of well solution and potassium iodide were collected using a Rigaku Micromax-007HF X-ray diffractometer with a rotating copper anode and a HyPix-6000HE Hybrid Photon Counting (HPC) X-ray detector. X-ray diffraction data were also collected from a single epi-Novo29 crystal using a synchrotron X-ray source (Advanced Light Source beamline 5.0.2) and a Pilatus3 6M 25 Hz detector. The dataset was indexed and integrated with XDS and scaled and merged with pointless and aimless. The crystallographic phase determination was done with Phaser. The structure was refined using phenix.refine, with manipulation of the model performed using Coot. Data collection and refinement statistics are shown Tables S2 and S3.

References

1. Gao, B.; Sharpless, K. B. Vicinal diol cyclic sulfates. Like epoxides only more reactive. *J. Am. Chem. Soc.* **1988**, *110*, 7538–7539.

2. He, L.; Byun, H.S.; Bittman, R. Efficient synthesis of chiral α,β-epoxyesters via a cyclic sulfate intermediate. *Tetrahedron Lett.* **1998**, *39*, 2071–2074.

3. France, B.; Bruno, V.; Nicolas, I. Synthesis of a protected derivative of (2R,3R)-β-hydroxyaspartic acid suitable for Fmoc-based solid phase synthesis. *Tetrahedron Lett.* **2013**, *54,* 158–161.

4. Guzmán-Martinez; A.; Vannieuwenhze, M. S. An Operationally Simple and Efficient Synthesis of Orthogonally Protected L-threo-beta-Hydroxyasparagine. *Synlett* **2007**, *10*, 1513–1516.

5. Liu, L.; Wang, B.; Bi, C.; He, G.; Chen, G. Efficient preparation of β-hydroxy aspartic acid and its derivatives. *Chin. Chem. Lett.* **2018**, *29*, 1113–1115.

6. The procedure for peptide synthesis follow closely to those that our laboratory has previously published. The procedures in this section were either adapted from or taken verbatim from: Yang, H.; Du Bois, D. R.; Ziller, J. W.; Nowick, J. S. X-ray crystallographic structure of a teixobactin analogue reveals key interactions of the teixobactin pharmacophore. *Chem. Commun.* **2017**, *53*, 2772–2775.

7. General procedures for NMR spectroscopy were either adapted from or taken verbatim from: Li, X.; Rios, S. E.; Nowick, J. S. Enantiomeric β-sheet peptides from Aβ form homochiral pleated β-sheets rather than heterochiral rippled β-sheets. *Chem. Sci.* **2022**, *13*, 7739–7746.

8. General procedures for minimum inhibitory concentration assays were either adapted from or taken verbatim from: Morris, M. A.; Malek, M.; Hashemian, M. H.; Nguyen, B. T.; Manuse, S.;

Lewis, K. L; Nowick, J. S. A Fluorescent Teixobactin Analogue. *ACS Chem. Biol.* **2020**, *15*, 1222–1231.

9. General procedures for X-ray crystallography were either adapted from or taken verbatim from: Li, X.; Sabol, A. L.; Wierzbicki, M.; Salveson, P. J.; Nowick, J. S. An improved turn structure for inducing β-hairpin formation in peptides. *Angew. Chem. Int. Ed.* **2021**, *60*, 22776–22782 and Samdin, T. D.; Wierzbicki, M.; Kreutzer, A. G.; Howitz, W. J.; Valenzuela, M.; Smith, A.; Sahrai, V.; Truex, N. L.; Klun, M.; Nowick, J. S. Effects of N-terminal residues on the assembly of constrained β-hairpin peptides derived from Aβ. *J. Am. Chem. Soc.* **2020**, *142*, 11593–11601.

Characterization Data

Characterization of natural Novo29

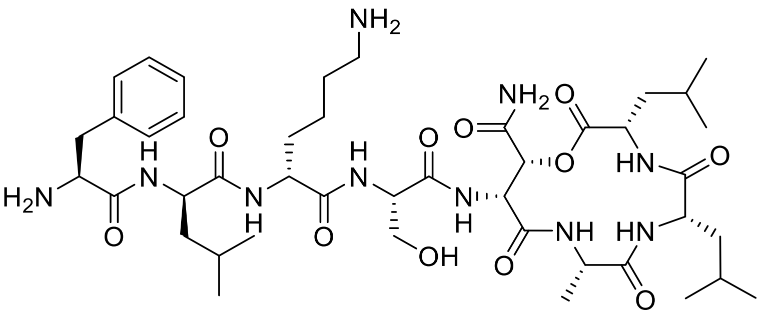

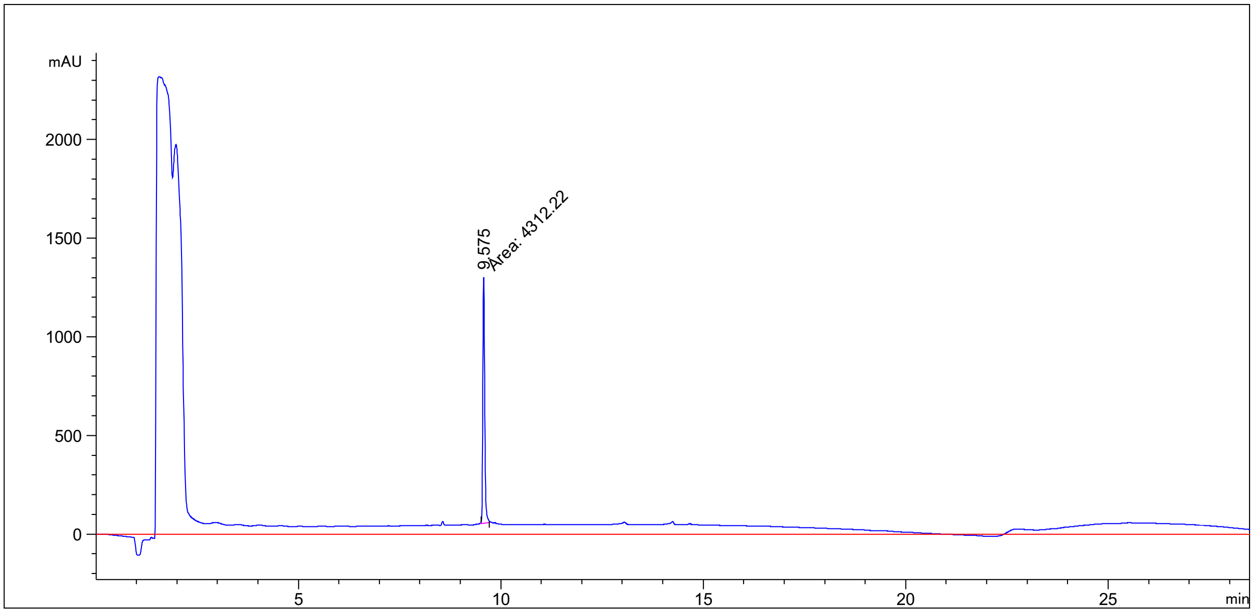

Signal 1: MWD1 A, Sig=214,4 Ref=off

Peak #	RetTime [min]	Type	Width [min]	Area [mAU*s]	Height [mAU]	Area %
1	9.575	MM	0.0569	4312.21582	1262.37280	100.0000

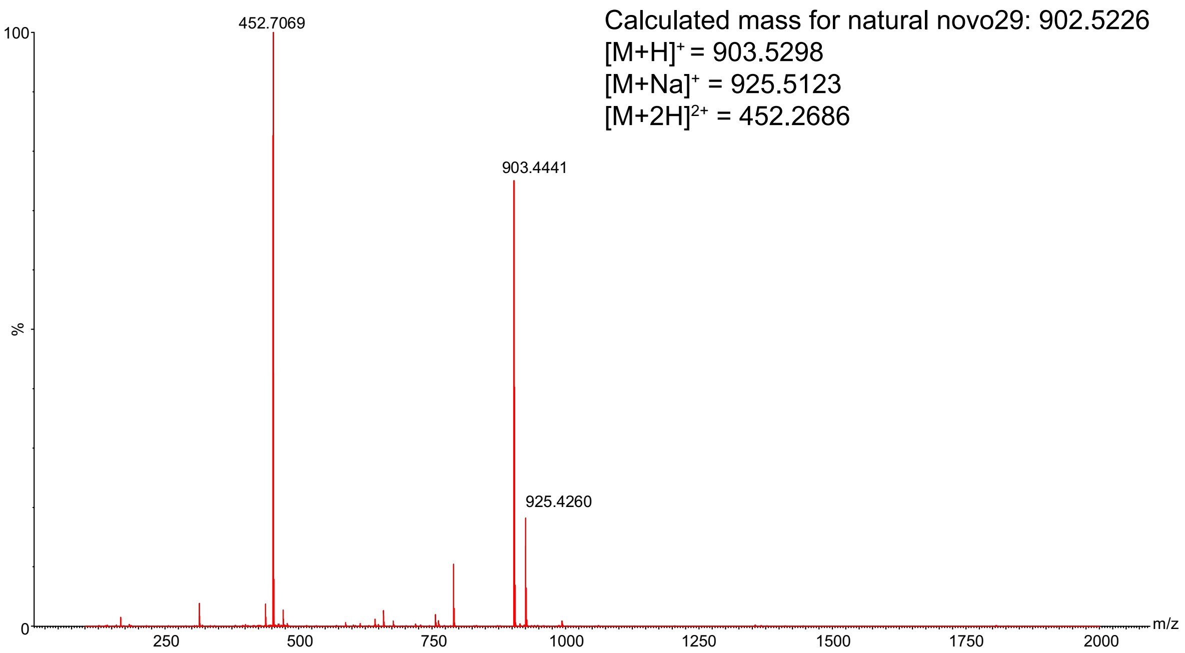
Calculated mass for natural novo29: 902.5226
[M+H]+ = 903.5298
[M+Na]+ = 925.5123
[M+2H]2+ = 452.2686
452.7069
903.4441
925.4260
100
%
0
250
500
750
1000
1250
1500
1750
2000
m/z

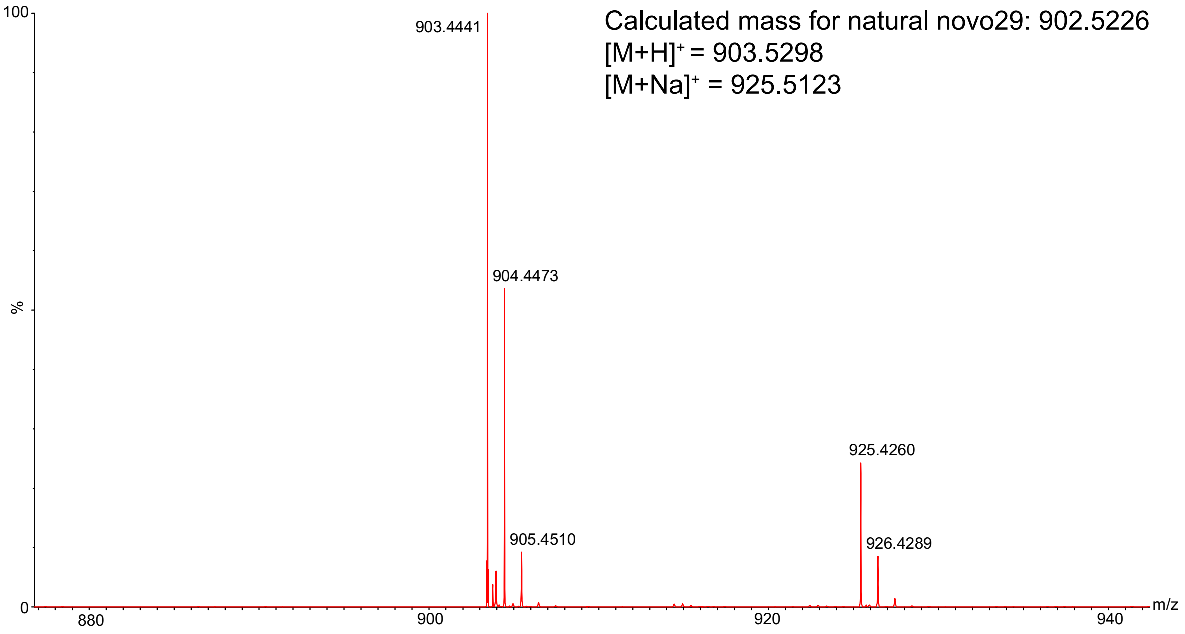
Calculated mass for natural novo29: 902.5226
[M+H]+ = 903.5298
[M+Na]+ = 925.5123
903.4441
904.4473
905.4510
925.4260
926.4289
100
%
0
880
900
920
940
m/z

Characterization of synthetic Novo29

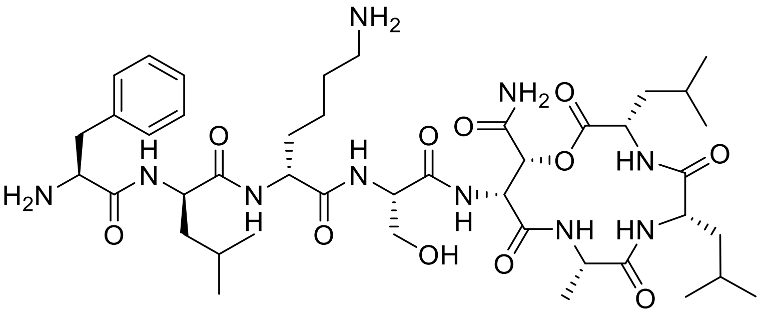

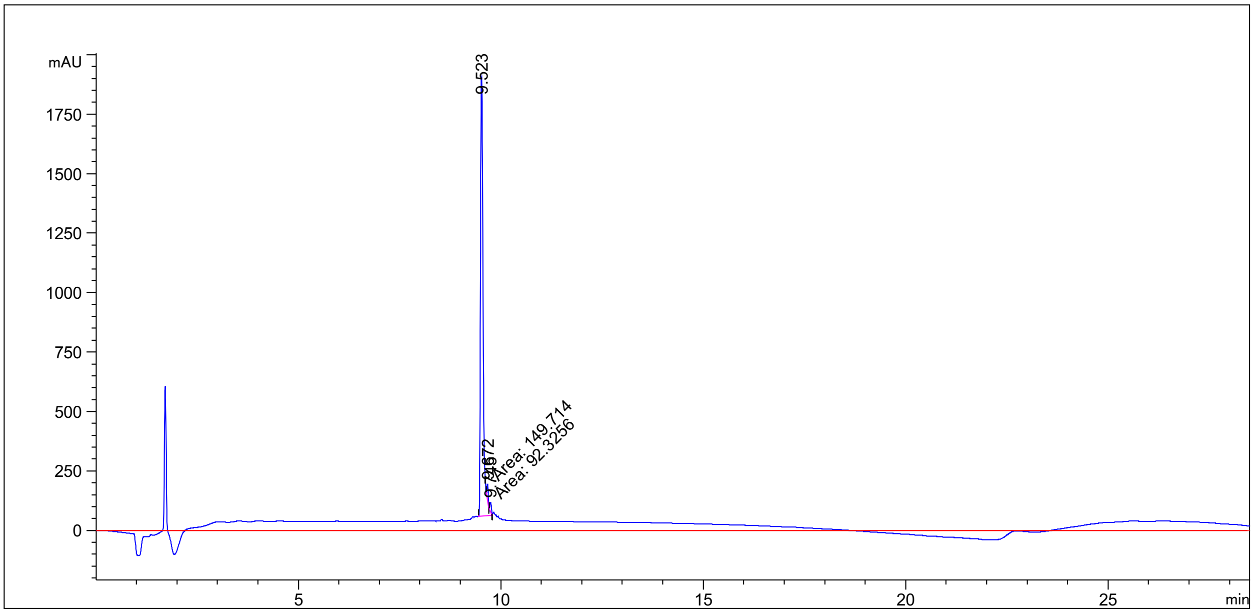

Signal 1: MWD1 A, Sig=214,4 Ref=off

Peak #	RetTime [min]	Type	Width [min]	Area [mAU*s]	Height [mAU]	Area %
1	9.523	MM R	0.0728	8129.04150	1861.80823	97.1086
2	9.672	MM T	0.0363	149.71405	68.68908	1.7885
3	9.745	MM T	0.0403	92.32558	38.15857	1.1029

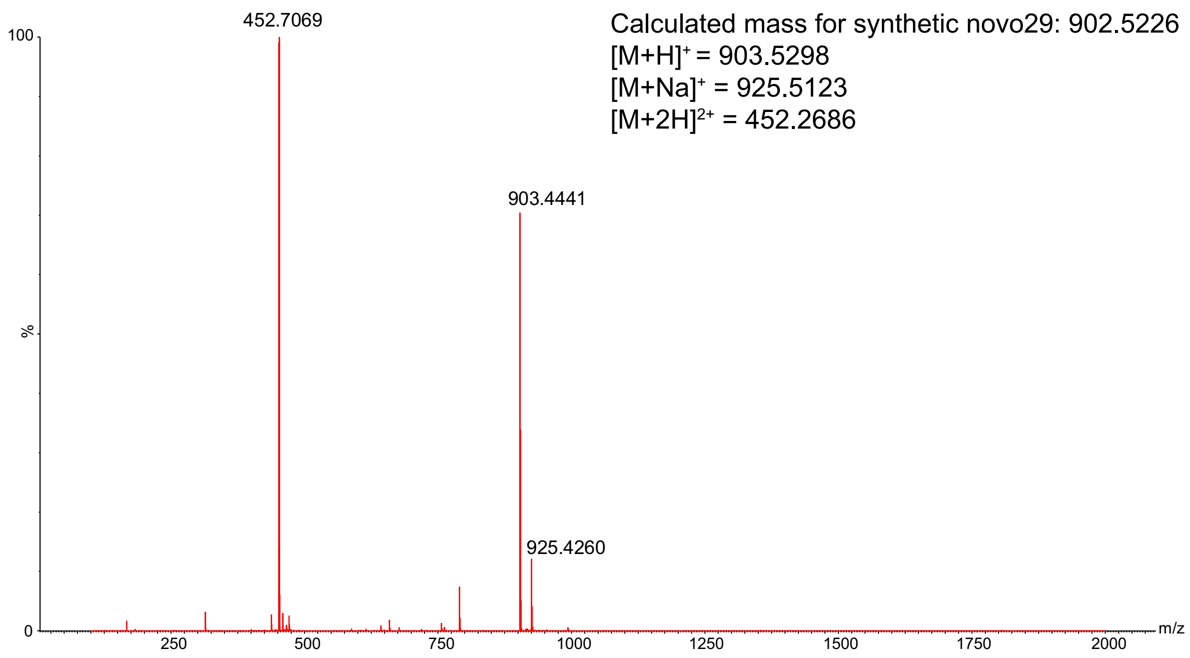
Calculated mass for synthetic novo29: 902.5226
[M+H]+ = 903.5298
[M+Na]+ = 925.5123
[M+2H]2+ = 452.2686
452.7069
903.4441
925.4260
100
%
0
m/z
250
500
750
1000
1250
1500
1750
2000

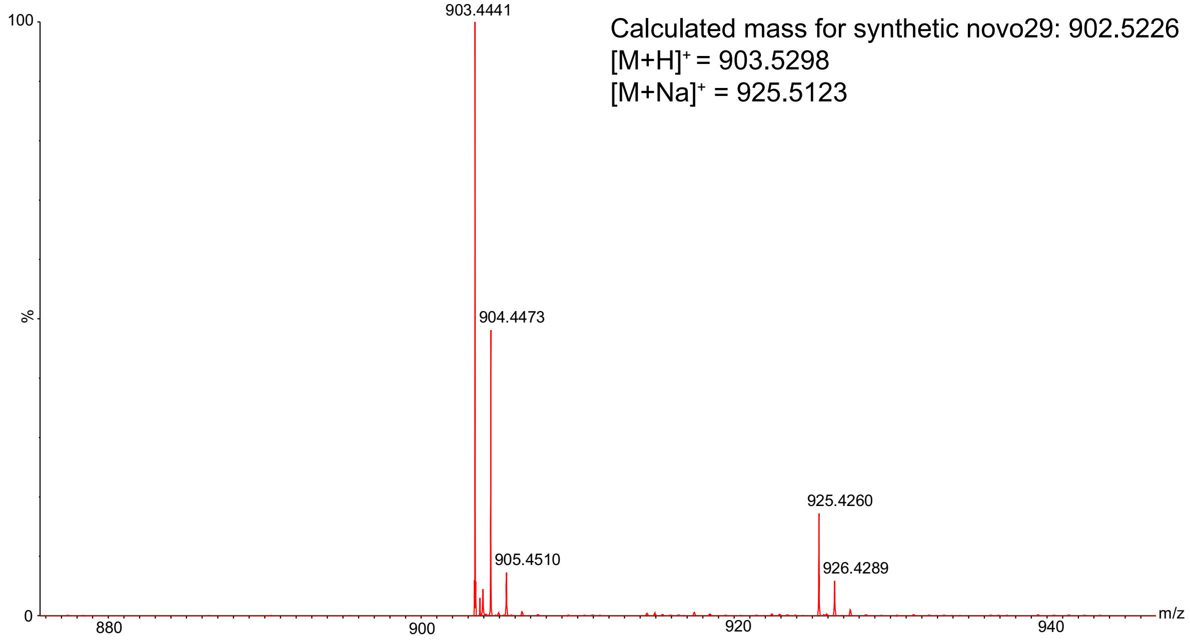
Calculated mass for synthetic novo29: 902.5226
[M+H]+ = 903.5298
[M+Na]+ = 925.5123
903.4441
904.4473
905.4510
925.4260
926.4289
100
%
0
m/z
880
900
920
940

Characterization of synthetic epi-Novo29

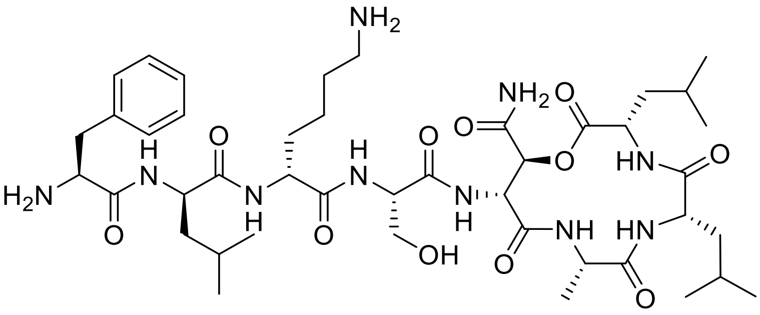

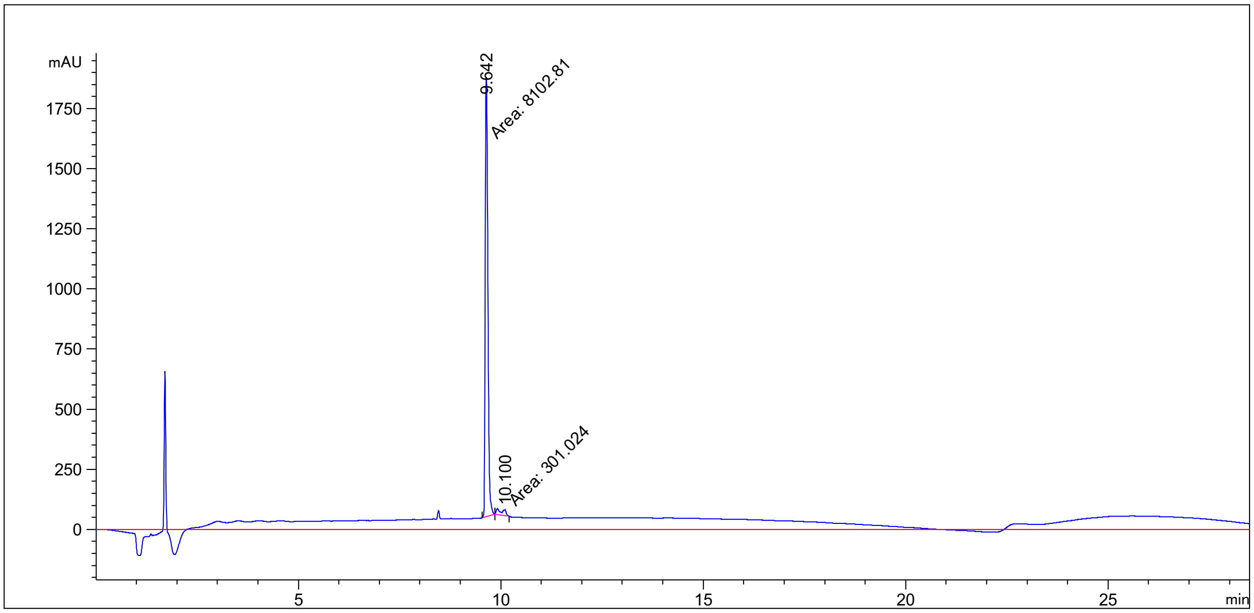

Signal 1: MWD1 A, Sig=214,4 Ref=off

Peak #	RetTime [min]	Type	Width [min]	Area [mAU*s]	Height [mAU]	Area %
1	9.642	MM	0.0735	8102.80859	1836.76953	96.4180
2	10.100	MM	0.2067	301.02429	24.26878	3.5820

Calculated mass for synthetic *epi*-novo29: 902.5226
$[M+H]^+$ = 903.5298
$[M+Na]^+$ = 925.5123
$[M+2H]^{2+}$ = 452.2686

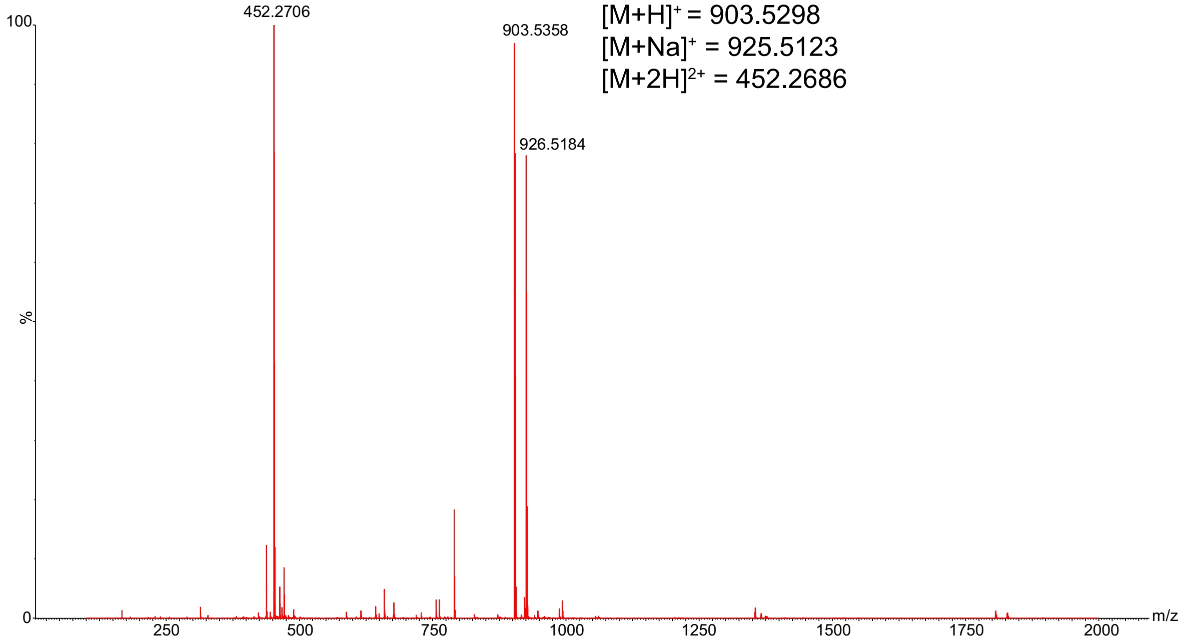

Calculated mass for synthetic *epi*-novo29: 902.5226
$[M+H]^+$ = 903.5298
$[M+Na]^+$ = 925.5123

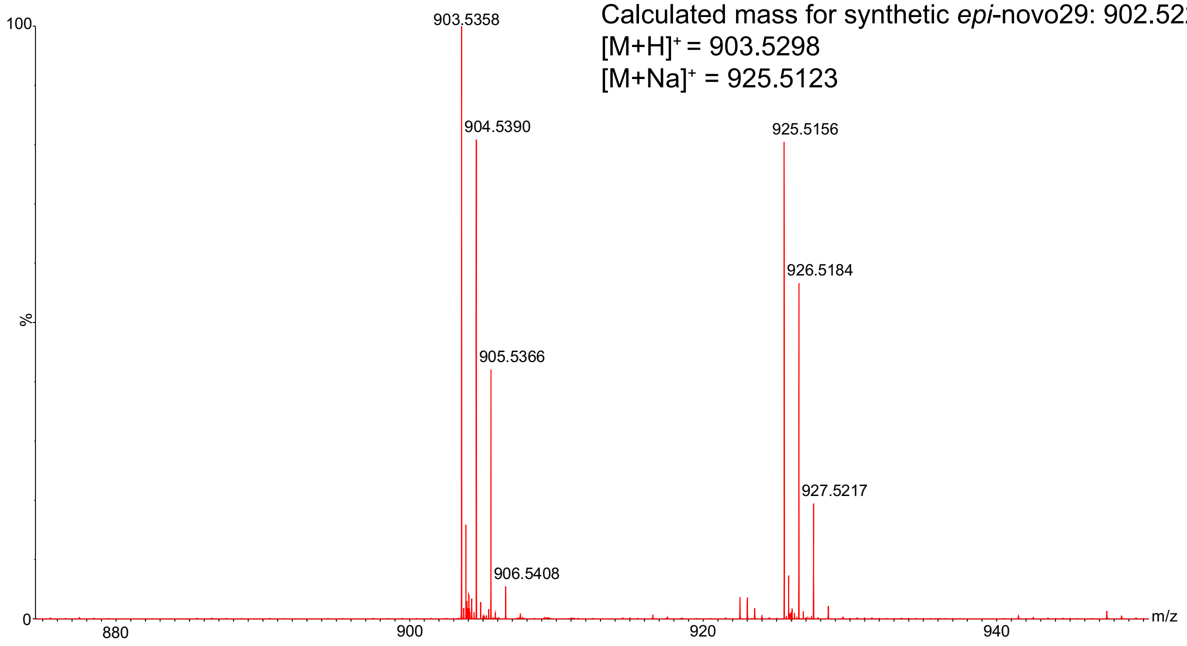

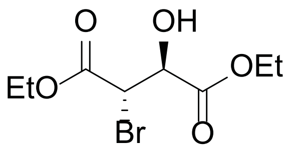

bromo alcohol **1**

^1H NMR (500 MHz, $CDCl_3$)

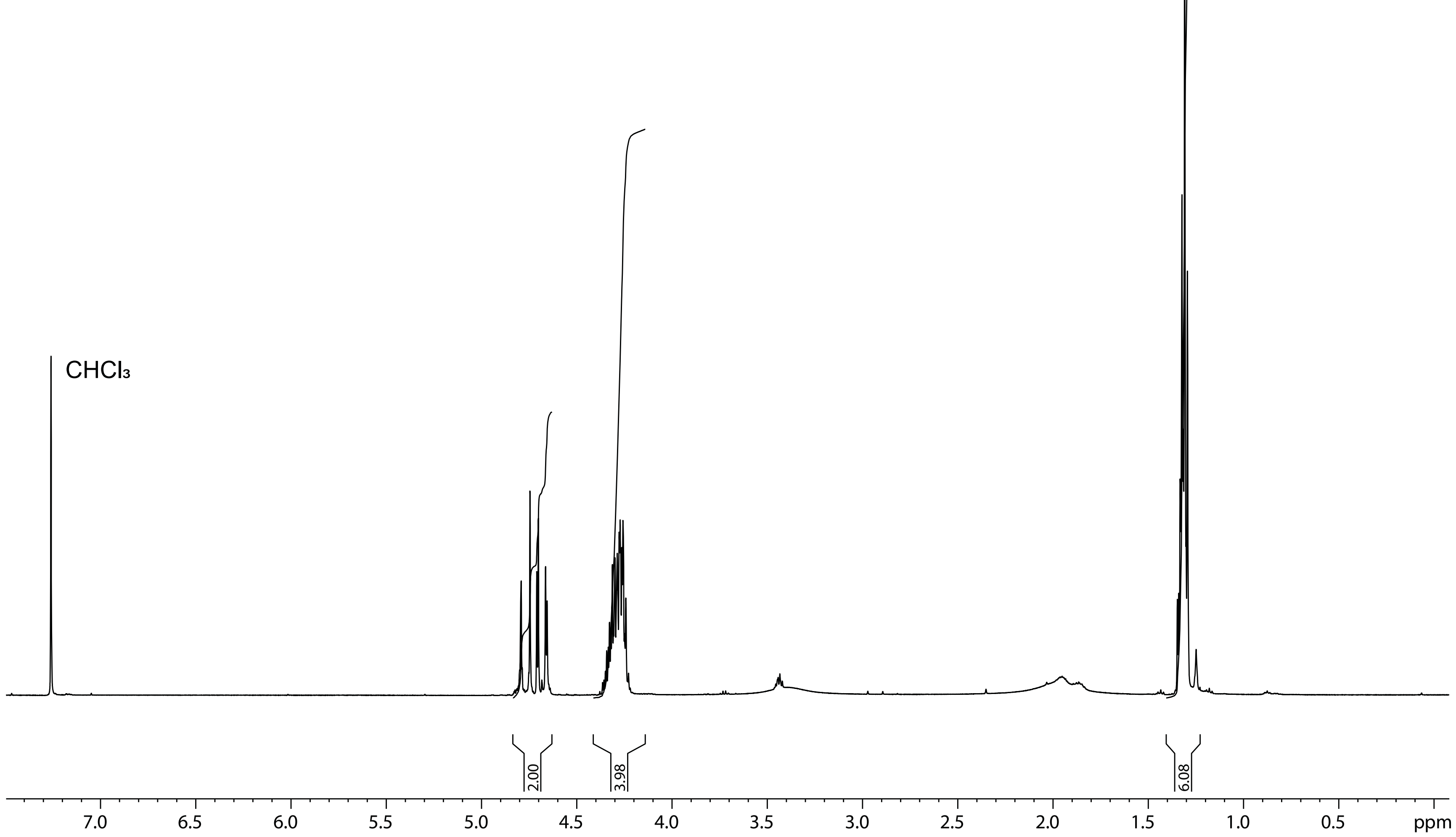

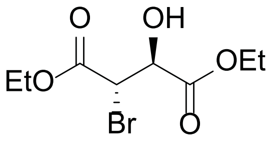

bromo alcohol **1**

$^{13}C\{^1H\}$ NMR (125.7 MHz, $CDCl_3$)

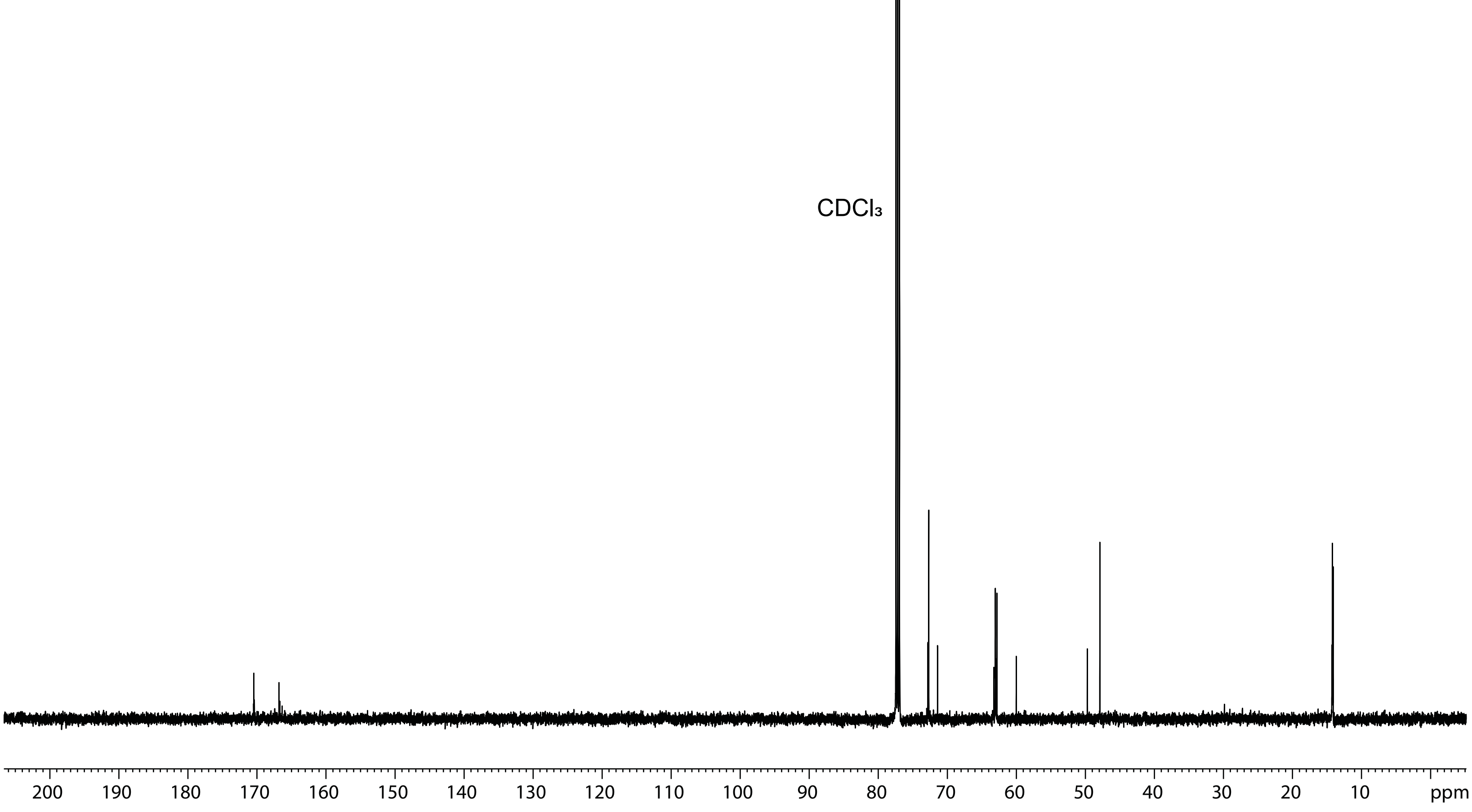

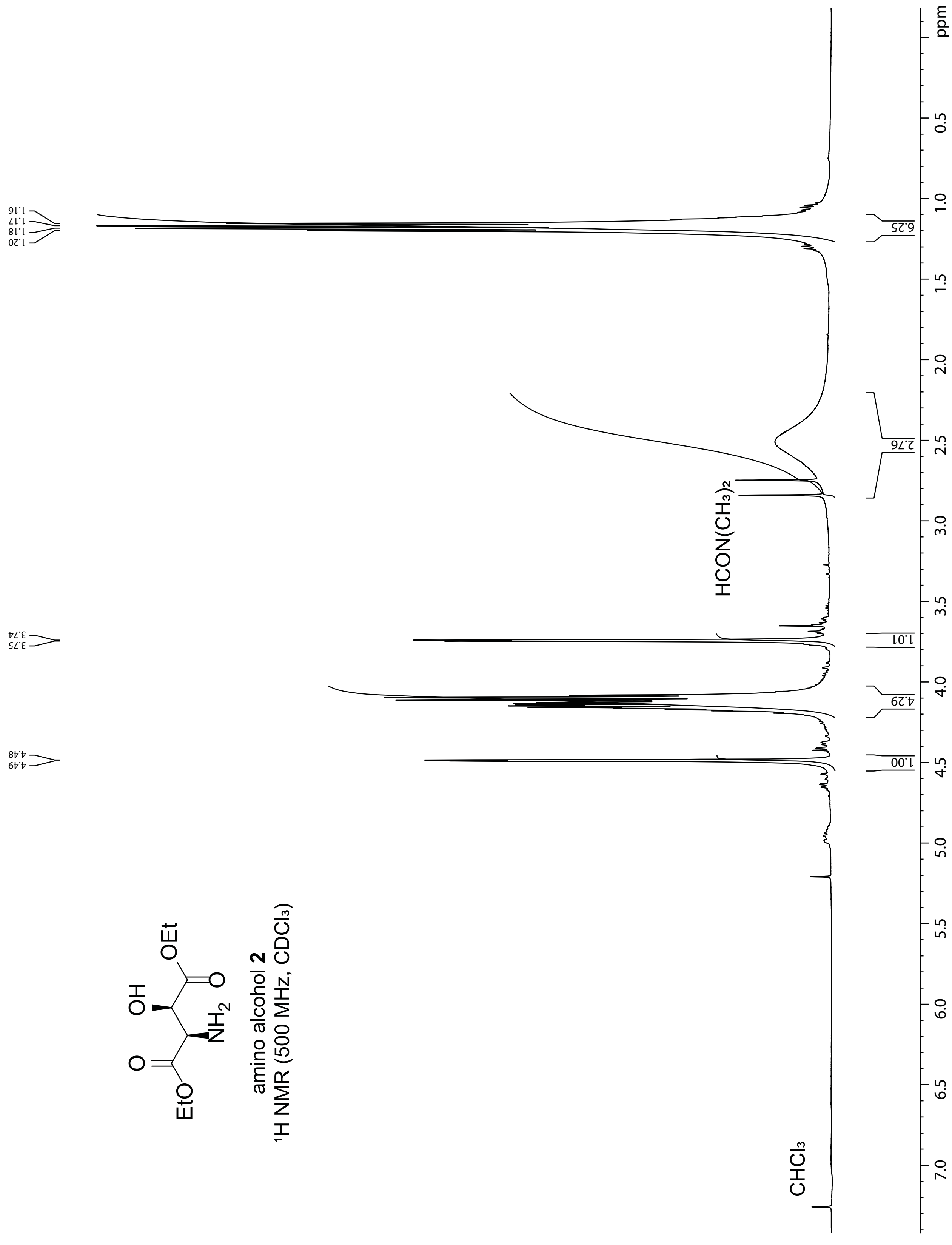

EtO
O
OH
NH2
O
OEt
amino alcohol 2
1H NMR (500 MHz, CDCl3)
CHCl3
HCON(CH3)2
1.20
1.18
1.17
1.16
3.75
3.74
4.49
4.48
6.25
2.76
1.01
4.29
1.00
ppm

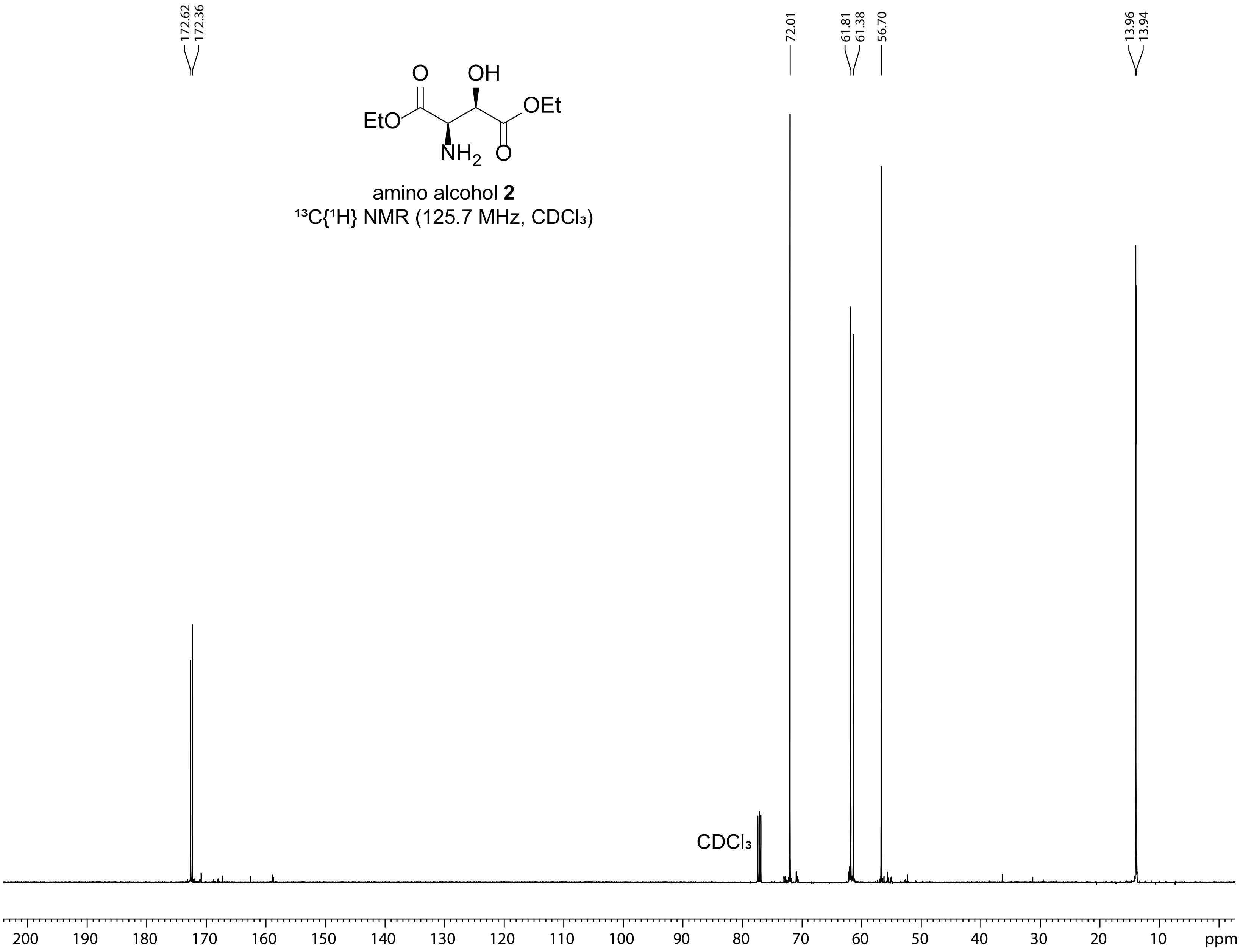

amino alcohol **2**
$^{13}C\{^{1}H\}$ NMR (125.7 MHz, $CDCl_3$)

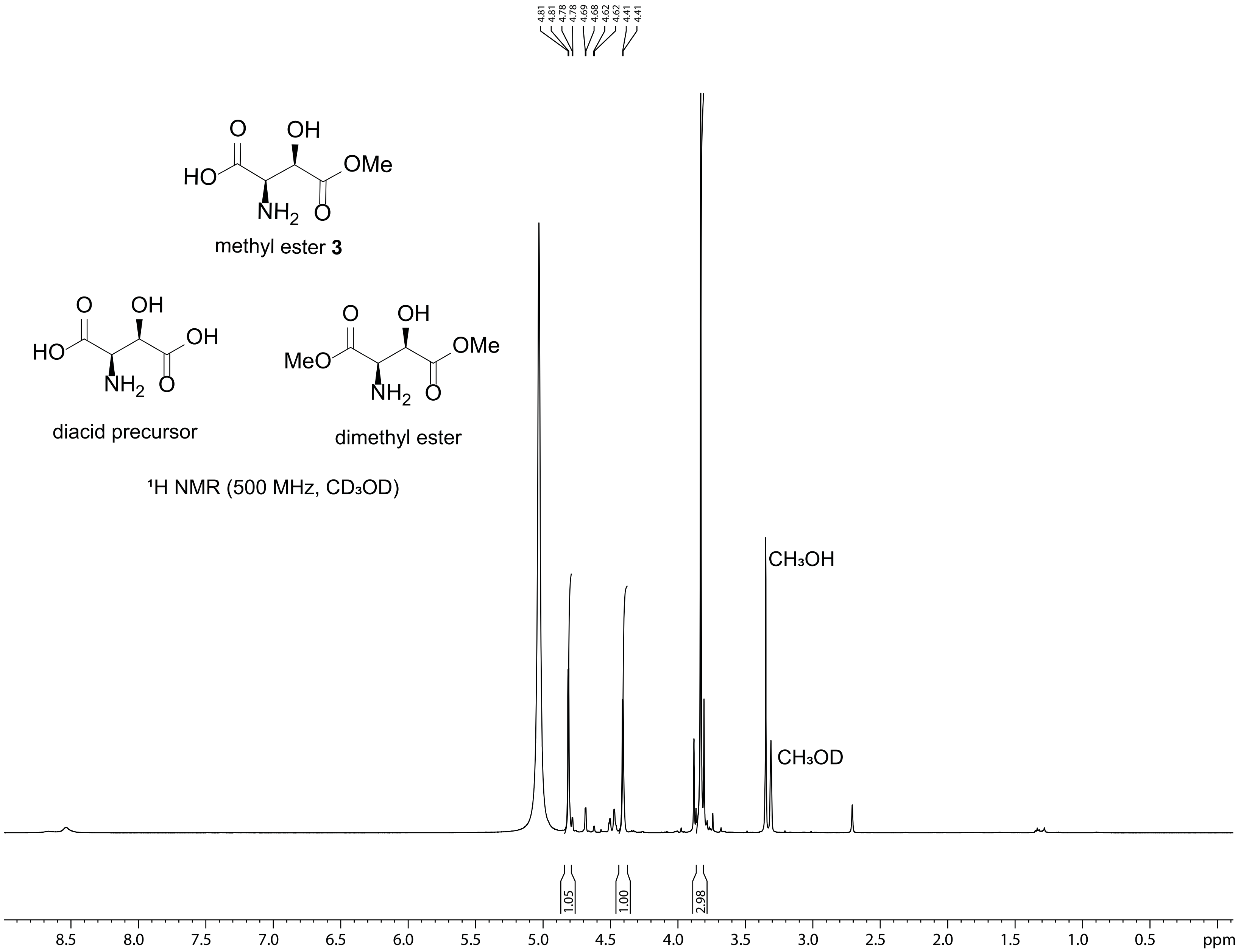
4.81
4.81
4.78
4.78
4.69
4.68
4.62
4.62
4.41
4.41
methyl ester 3
diacid precursor
dimethyl ester
¹H NMR (500 MHz, CD₃OD)
CH₃OH
CH₃OD
1.05
1.00
2.98
8.5
8.0
7.5
7.0
6.5
6.0
5.5
5.0
4.5
4.0
3.5
3.0
2.5
2.0
1.5
1.0
0.5
ppm

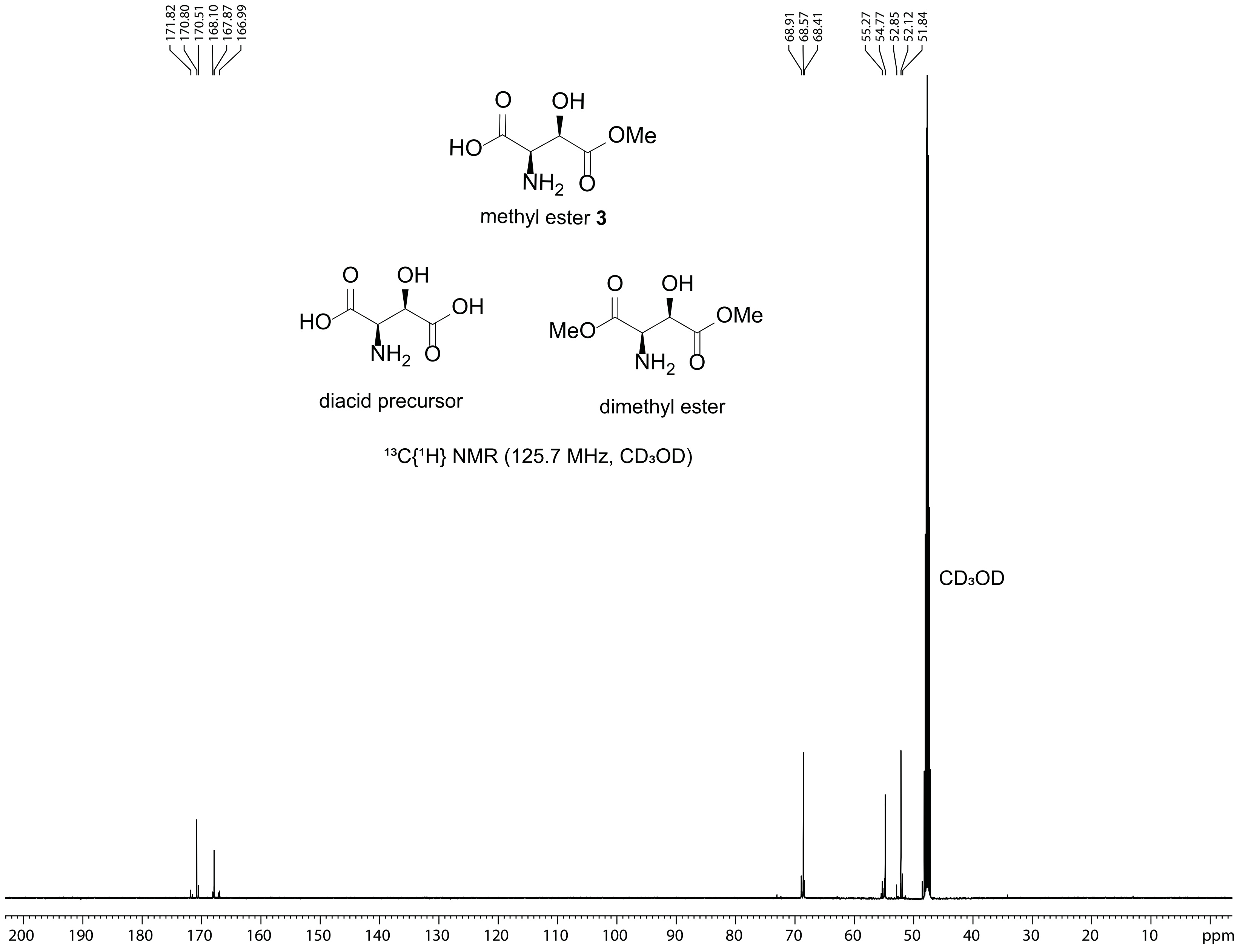
171.82
170.80
170.51
168.10
167.87
166.99
68.91
68.57
68.41
55.27
54.77
52.85
52.12
51.84
methyl ester 3
diacid precursor
dimethyl ester
$^{13}C\{^1H\}$ NMR (125.7 MHz, CD_3OD)
CD_3OD
ppm

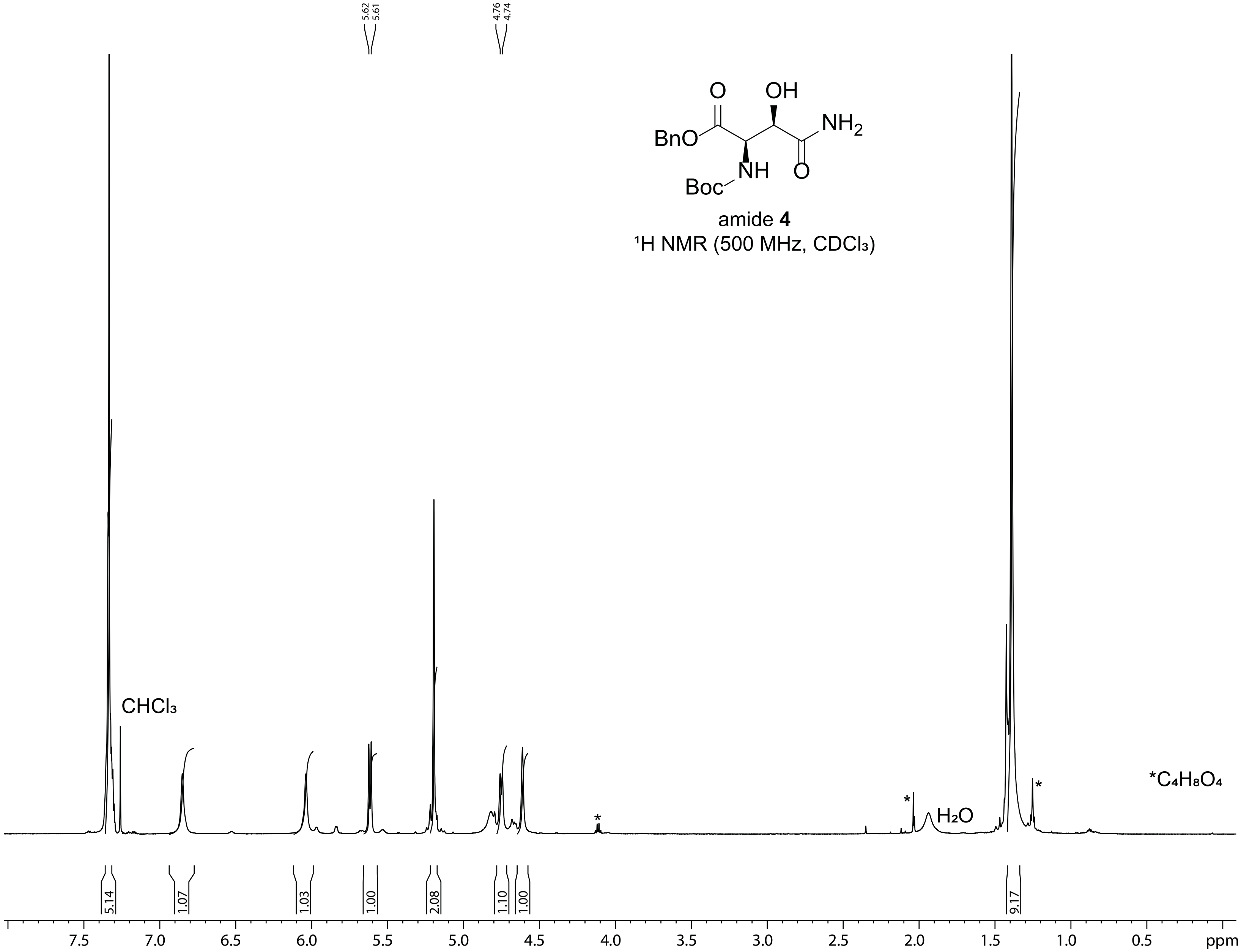

amide **4**
^{1}H NMR (500 MHz, $CDCl_3$)

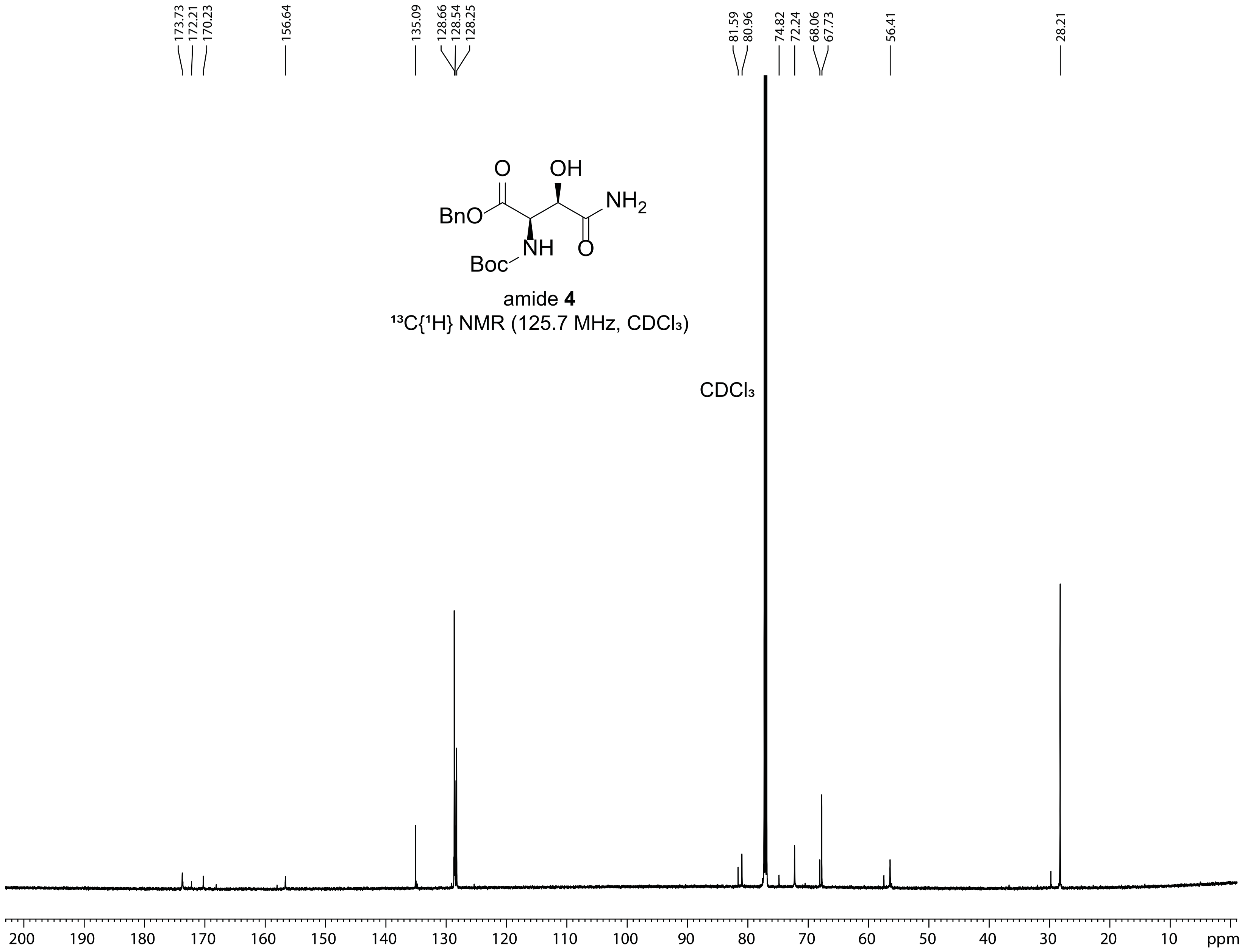
173.73
172.21
170.23
156.64
135.09
128.66
128.54
128.25
81.59
80.96
74.82
72.24
68.06
67.73
56.41
28.21
O
OH
BnO
NH2
NH
O
Boc
amide 4
13C{1H} NMR (125.7 MHz, CDCl3)
CDCl3
200
190
180
170
160
150
140
130
120
110
100
90
80
70
60
50
40
30
20
10
ppm

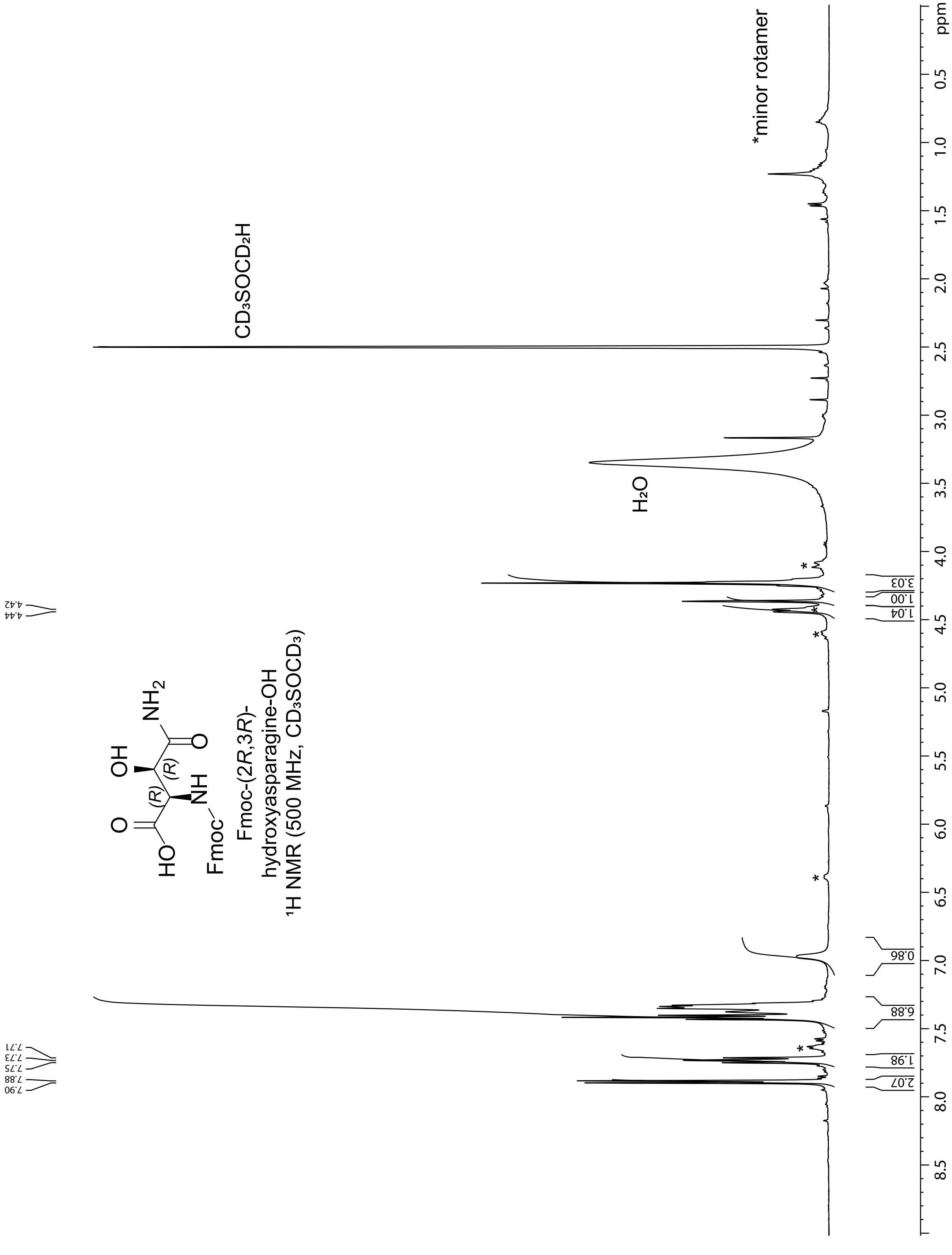

7.90
7.88
7.75
7.73
7.71
4.44
4.42
HO
O
OH
NH2
Fmoc
NH
(R)
(R)
O
Fmoc-(2R,3R)-
hydroxyasparagine-OH
1H NMR (500 MHz, CD3SOCD3)
CD3SOCD2H
H2O
*minor rotamer
2.07
1.98
6.88
0.86
1.04
1.00
3.03
8.5
8.0
7.5
7.0
6.5
6.0
5.5
5.0
4.5
4.0
3.5
3.0
2.5
2.0
1.5
1.0
0.5
ppm

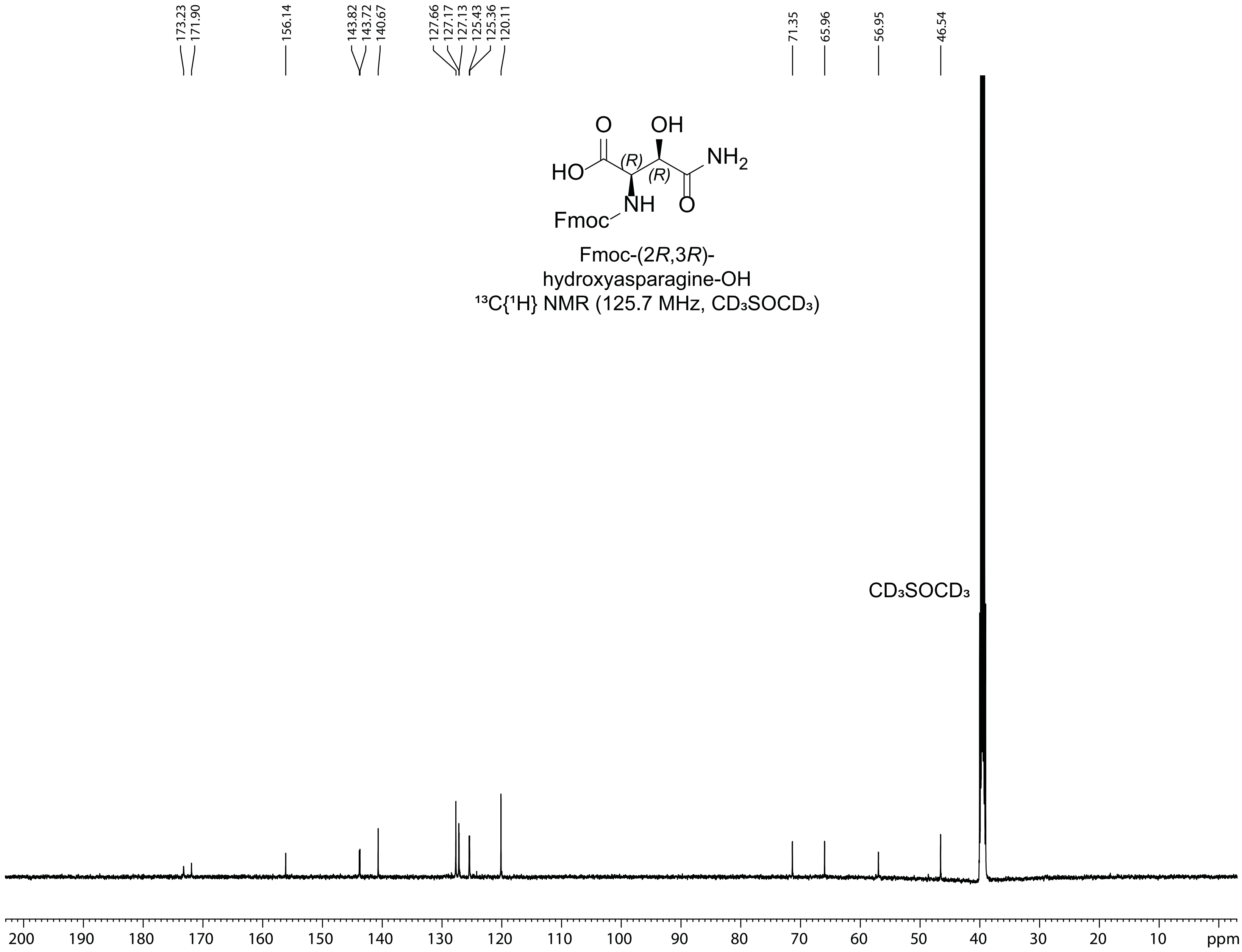
173.23
171.90
156.14
143.82
143.72
140.67
127.66
127.17
127.13
125.43
125.36
120.11
71.35
65.96
56.95
46.54
HO
O
OH
(R)
(R)
NH$_2$
NH
O
Fmoc
Fmoc-(2*R*,3*R*)-
hydroxyasparagine-OH
$^{13}C\{^1H\}$ NMR (125.7 MHz, CD_3SOCD_3)
CD_3SOCD_3
200 190 180 170 160 150 140 130 120 110 100 90 80 70 60 50 40 30 20 10 ppm

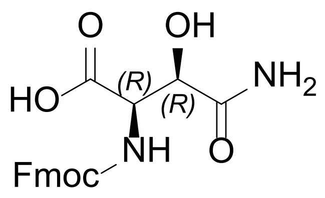

Fmoc-(2*R*,3*R*)-hydroxyAsn-OH

500 MHz EXSY (NOESY) spectrum of Fmoc-(2*R*,3*R*)-hydroxyAsn illustrating the exchange between rotamers.
800-ms mixing time in DMSO-d_6, 298 K.
Cross peaks demonstrating exchange of protons from rotamers are circled in red.
Pairs of resonances associated with major and minor rotamers are designated A and A*, B and B*, etc.

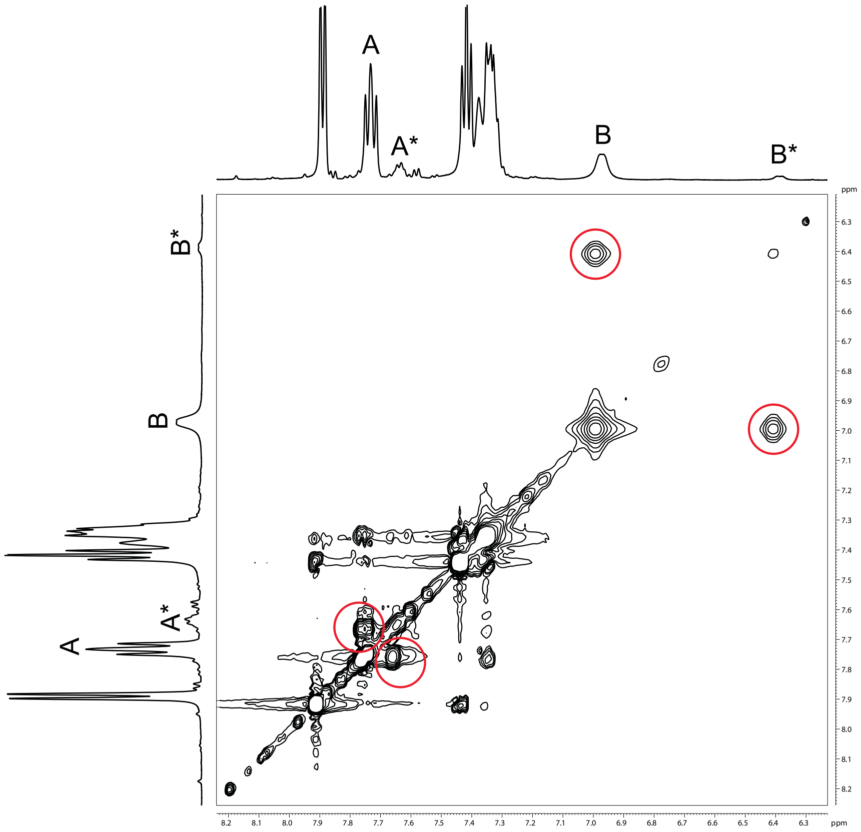

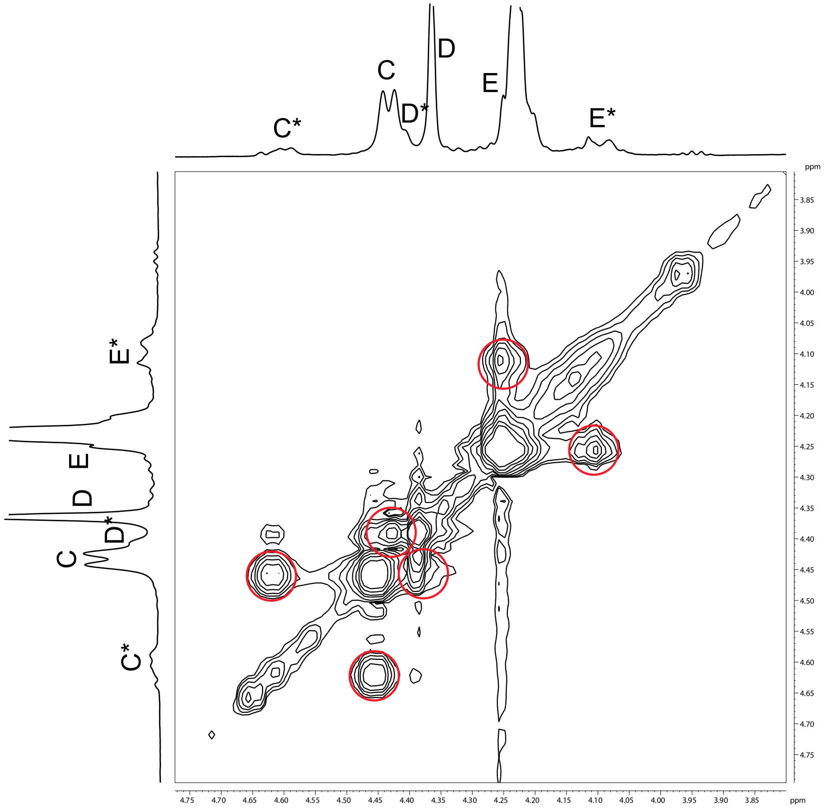

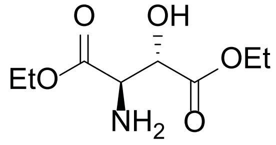

amino alcohol **5**
^1H NMR (500 MHz, $CDCl_3$)

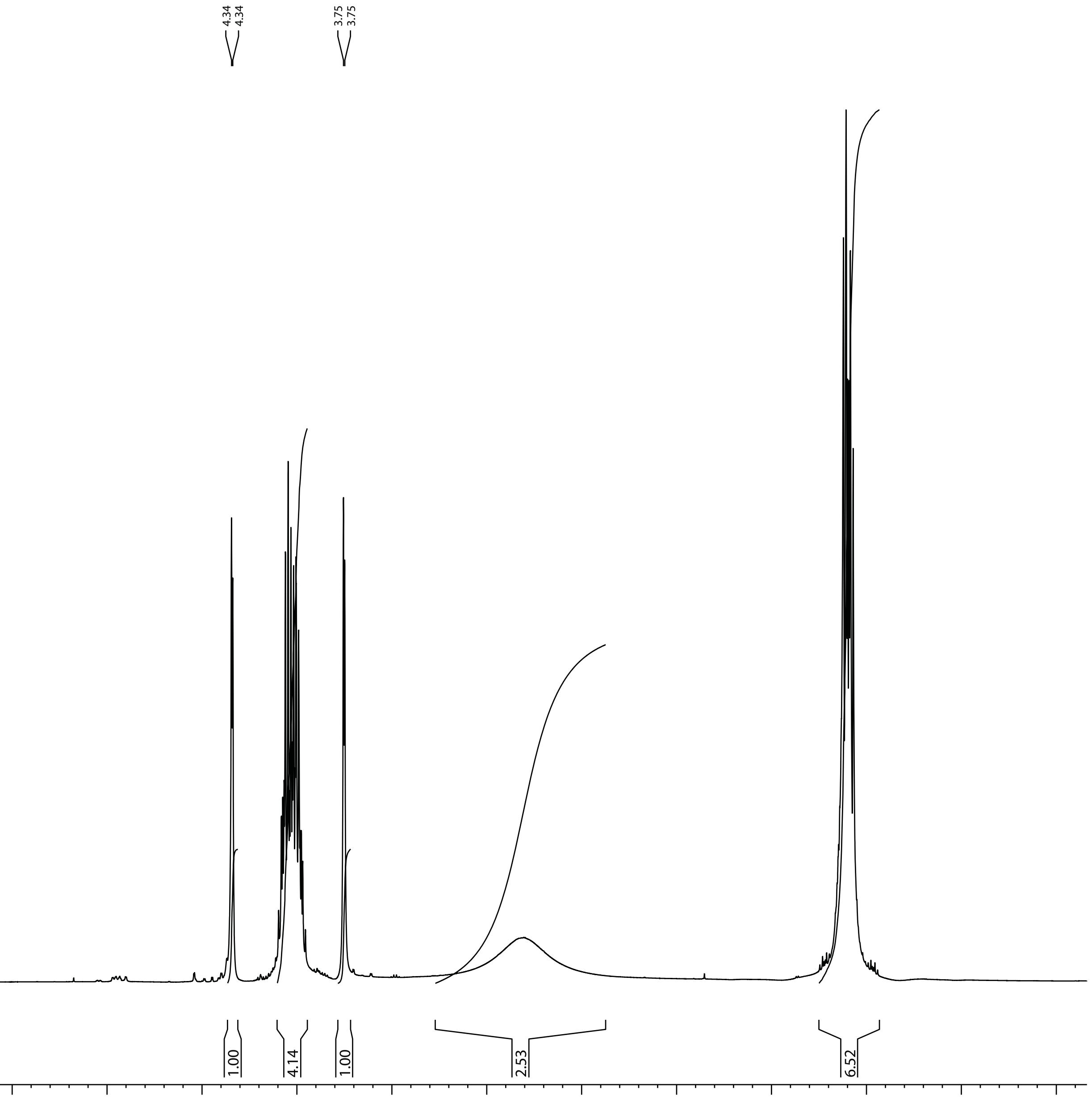

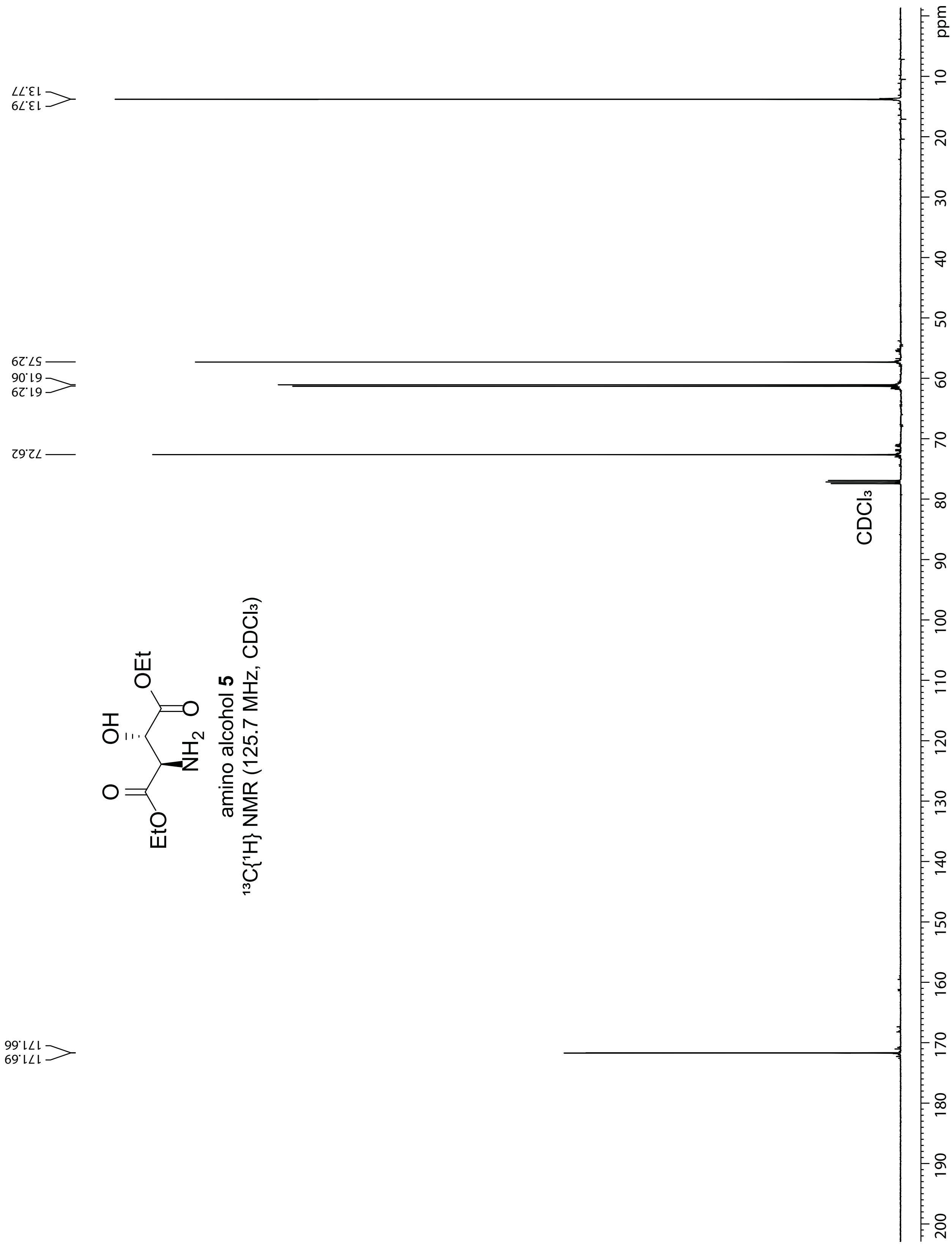
13.77
13.79
57.29
61.06
61.29
72.62
171.66
171.69
CDCl3
amino alcohol 5
13C{1H} NMR (125.7 MHz, CDCl3)
EtO
O
OH
NH2
OEt
ppm
10
20
30
40
50
60
70
80
90
100
110
120
130
140
150
160
170
180
190
200

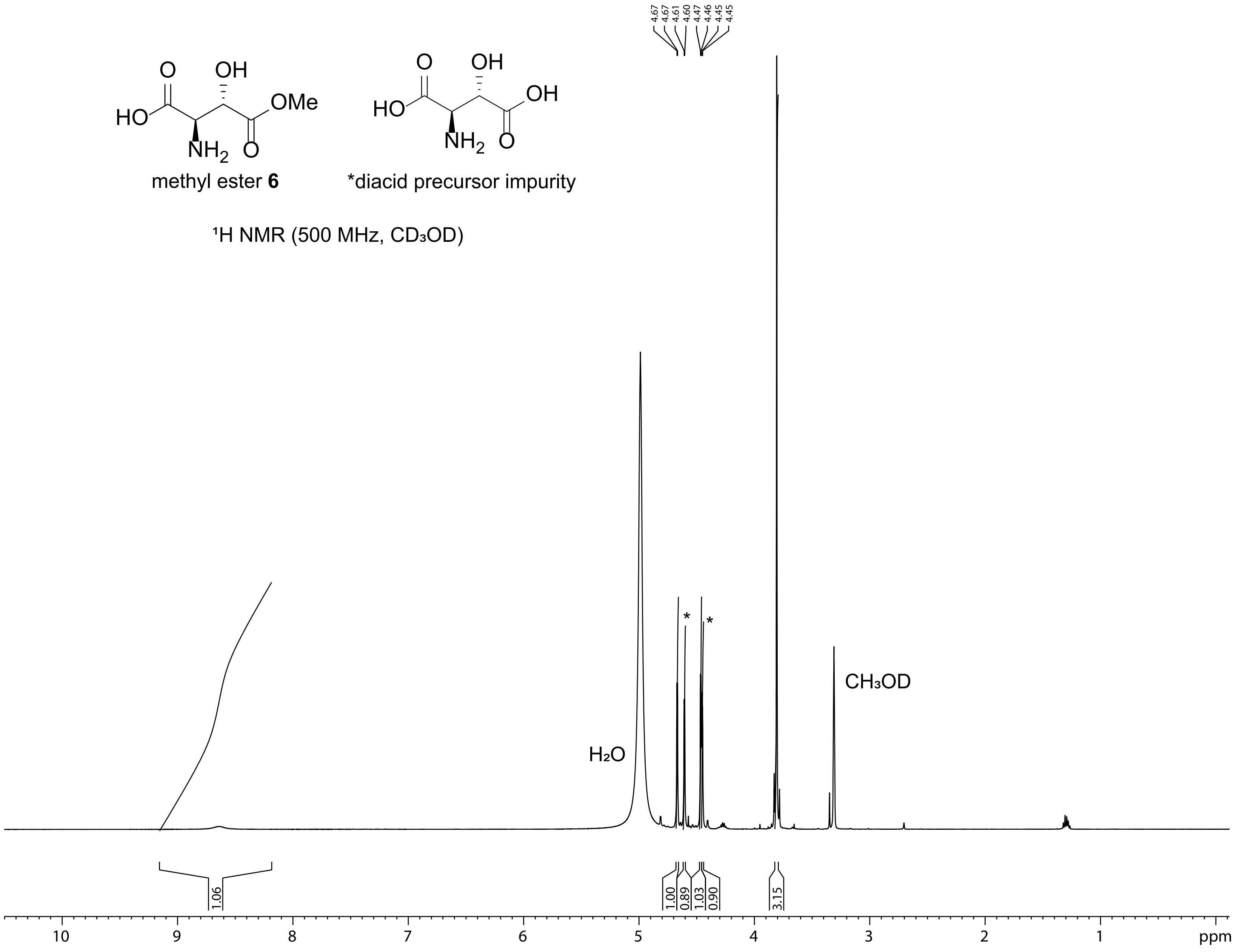

methyl ester **6**
*diacid precursor impurity
¹H NMR (500 MHz, CD₃OD)
4.67
4.67
4.61
4.60
4.47
4.46
4.45
4.45
H₂O
CH₃OD
1.06
1.00
0.89
1.03
0.90
3.15
10
9
8
7
6
5
4
3
2
1
ppm

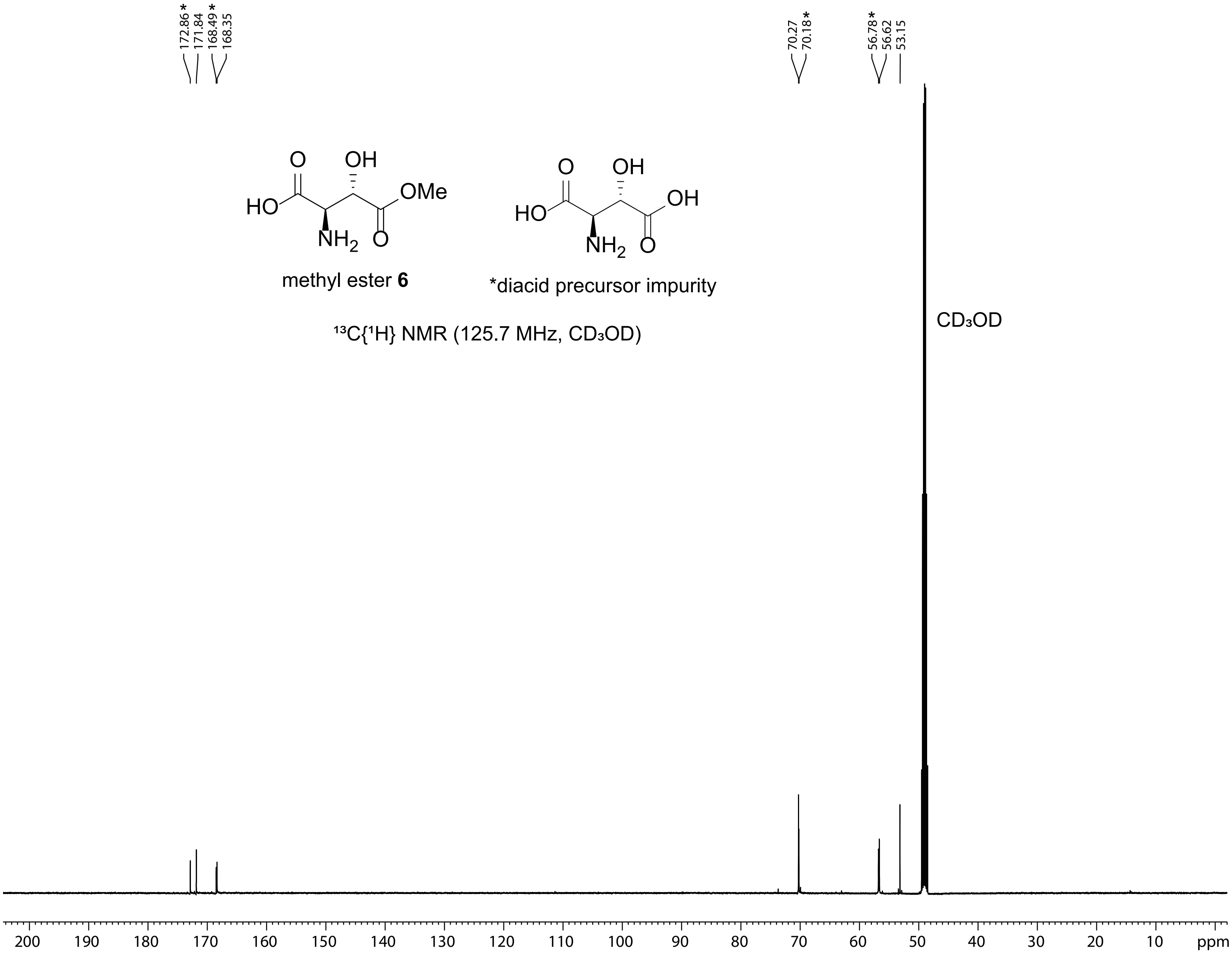
172.86*
171.84
168.49*
168.35
70.27
70.18*
56.78*
56.62
53.15
methyl ester 6
*diacid precursor impurity
$^{13}C\{^1H\}$ NMR (125.7 MHz, CD_3OD)
CD_3OD
200 190 180 170 160 150 140 130 120 110 100 90 80 70 60 50 40 30 20 10 ppm

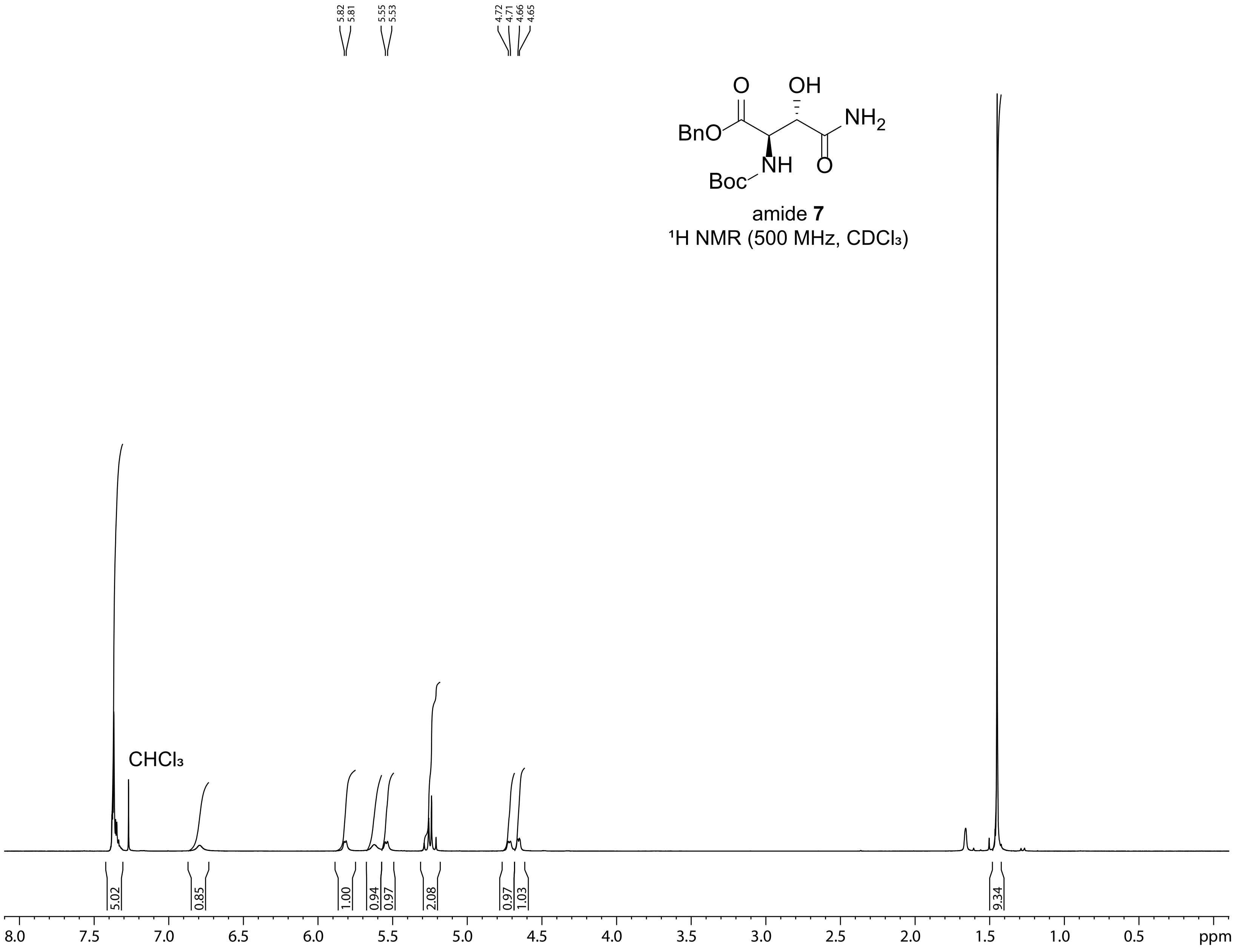

amide **7**

^1H NMR (500 MHz, $CDCl_3$)

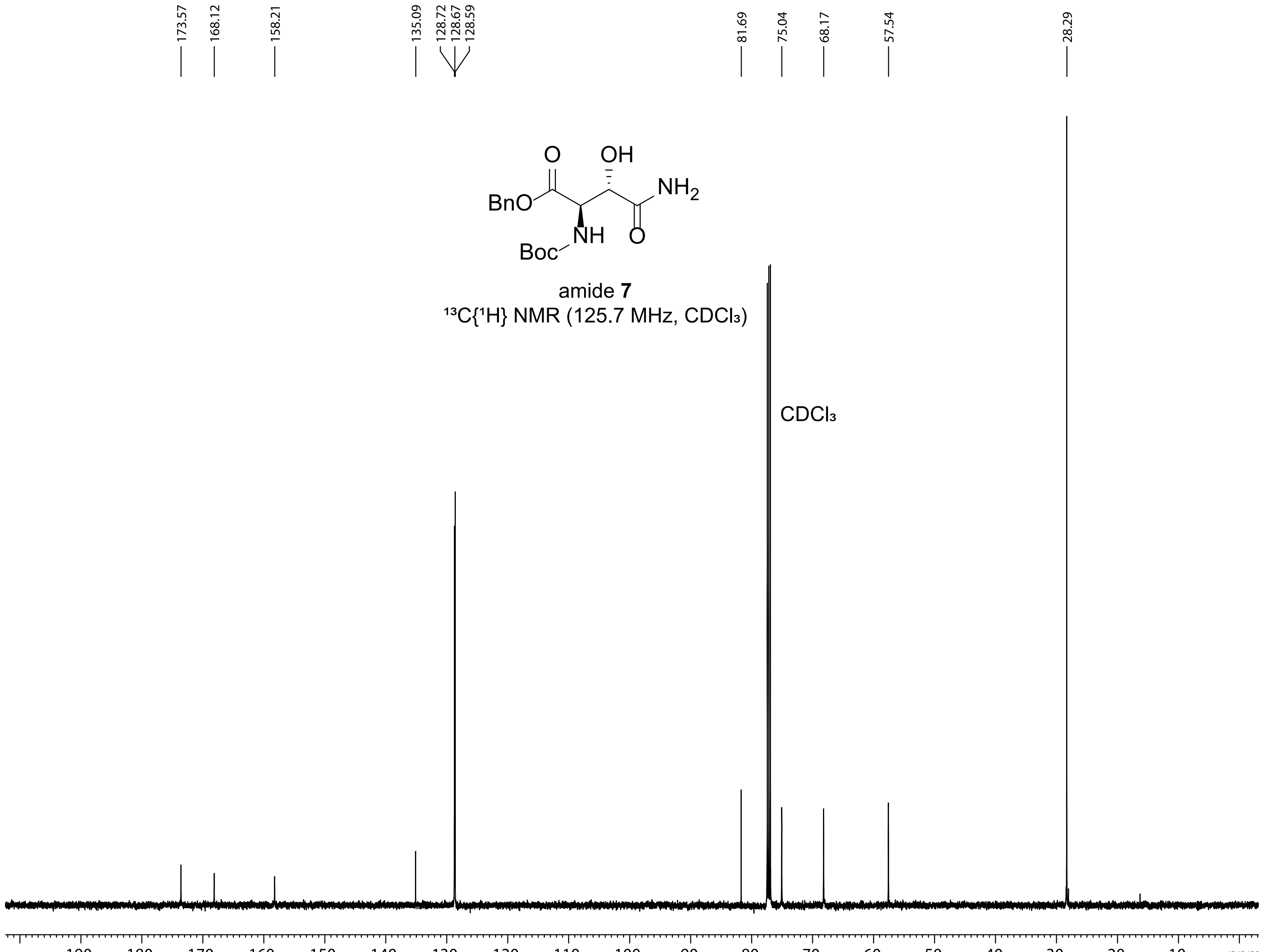
173.57
168.12
158.21
135.09
128.72
128.67
128.59
81.69
75.04
68.17
57.54
28.29
BnO
O
OH
NH2
NH
Boc
O
amide 7
13C{1H} NMR (125.7 MHz, CDCl3)
CDCl3
190 180 170 160 150 140 130 120 110 100 90 80 70 60 50 40 30 20 10 ppm

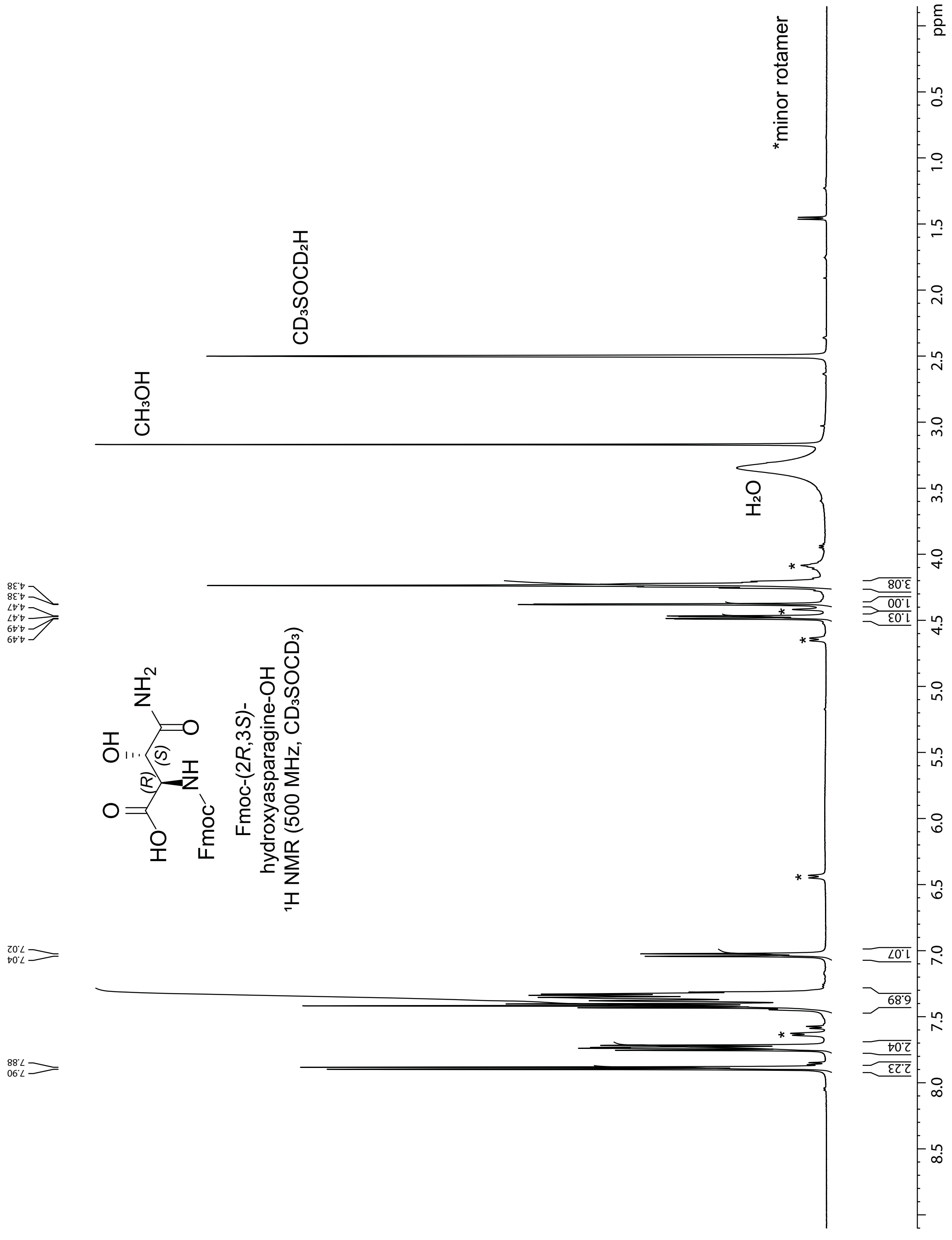
Fmoc-(2R,3S)-
hydroxyasparagine-OH
¹H NMR (500 MHz, CD₃SOCD₃)
CH₃OH
CD₃SOCD₂H
H₂O
*minor rotamer
ppm

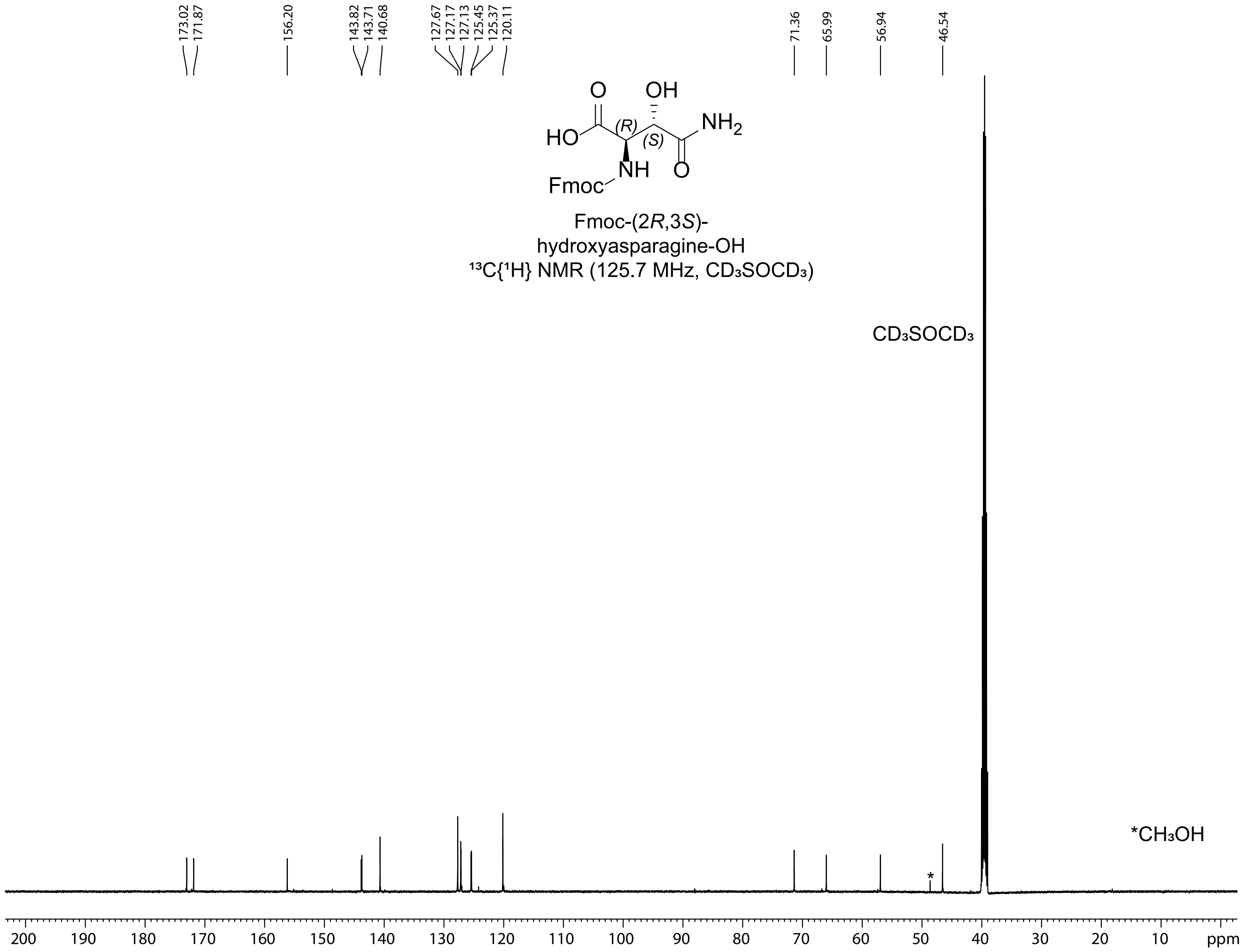
173.02
171.87
156.20
143.82
143.71
140.68
127.67
127.17
127.13
125.45
125.37
120.11
71.36
65.99
56.94
46.54
Fmoc-(2R,3S)-
hydroxyasparagine-OH
13C{1H} NMR (125.7 MHz, CD3SOCD3)
CD3SOCD3
*CH3OH
ppm

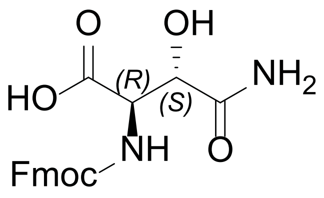

Fmoc-(2R,3S)-hydroxyAsn-OH

500 MHz EXSY (NOESY) spectrum of Fmoc-(2*R*,3*S*)-hydroxyAsn illustrating the exchange between rotamers.
1000-ms mixing time in DMSO-d_6, 298 K.
Cross peaks demonstrating exchange of protons from rotamers are circled in red.
Pairs of resonances associated with major and minor rotamers are designated A and A*, B and B*, etc.

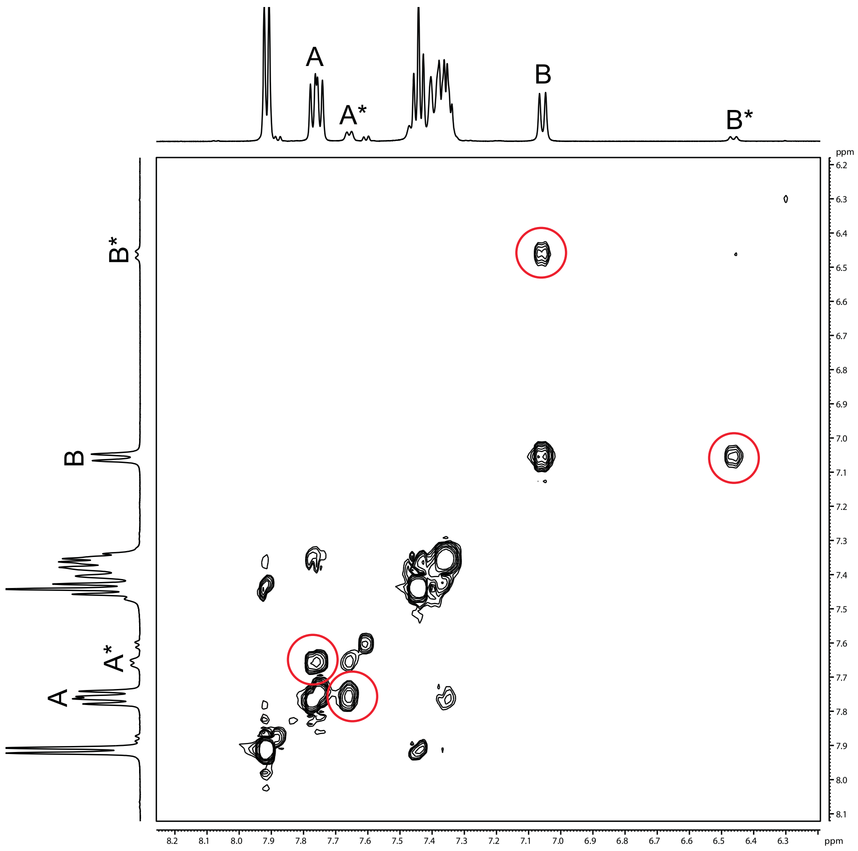

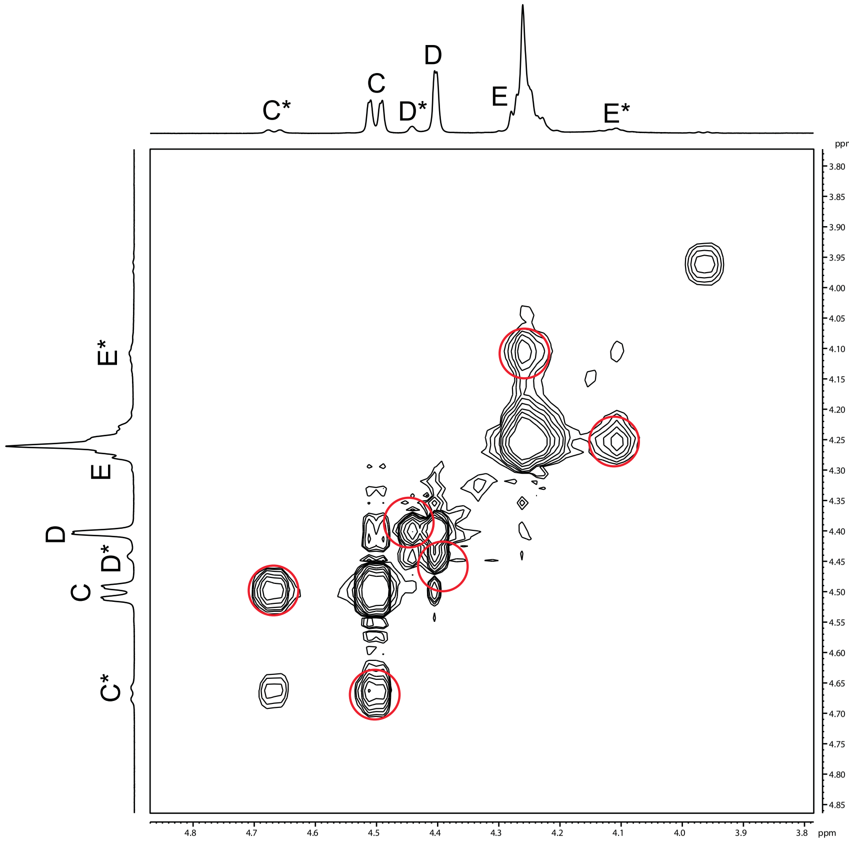

^{1}H NMR of 3 mM natural Novo29 in DMSO-d_6 at 500 MHz and 298 K

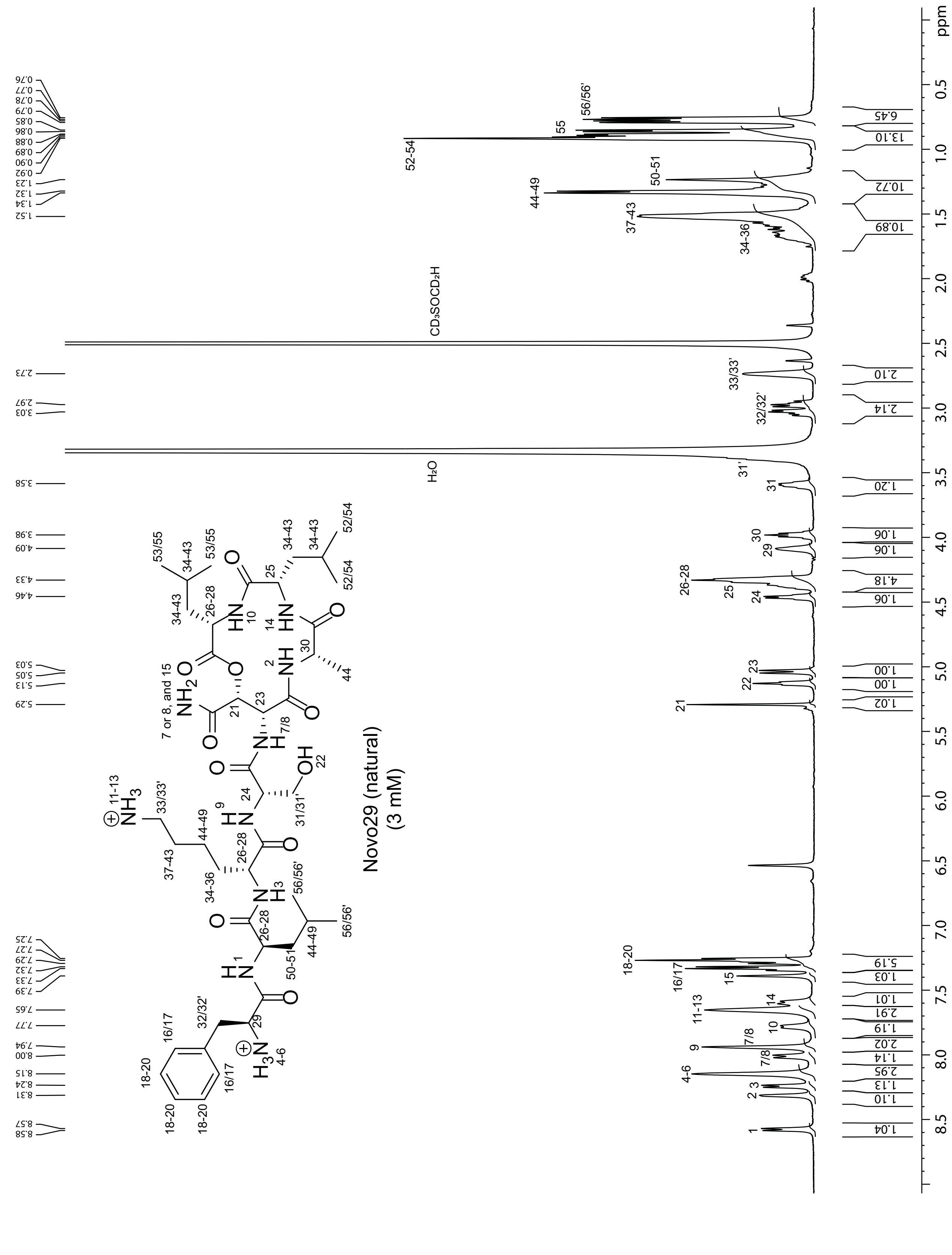

2.0 mM natural Novo29
in DMSO-d_6, 150-ms mixing time, 298 K
600 MHz TOCSY spectrum

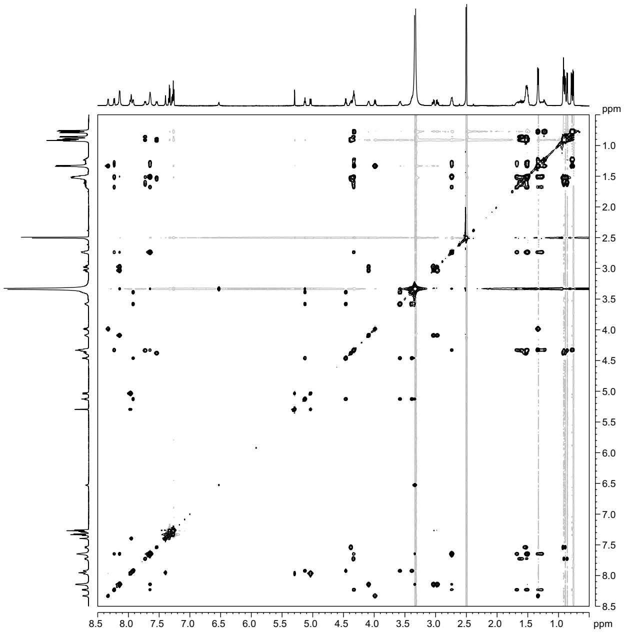

Novo29

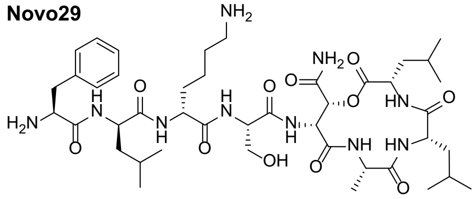

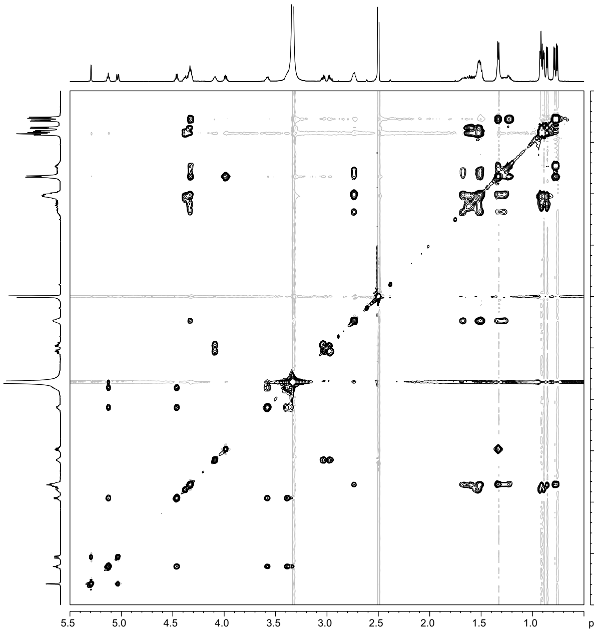

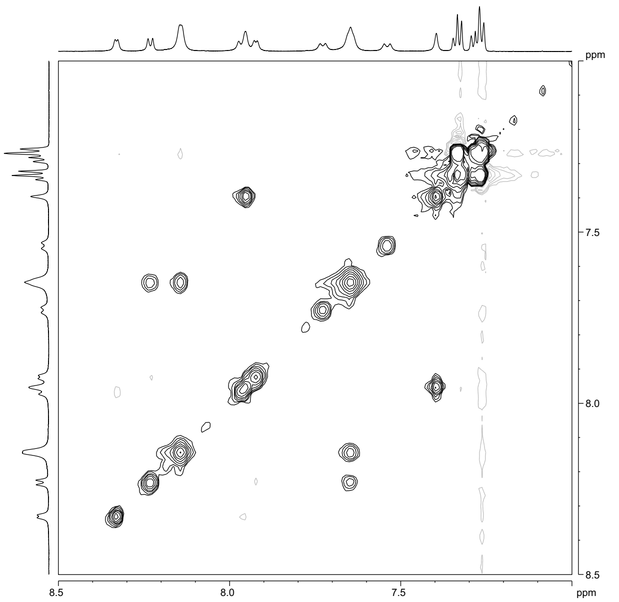

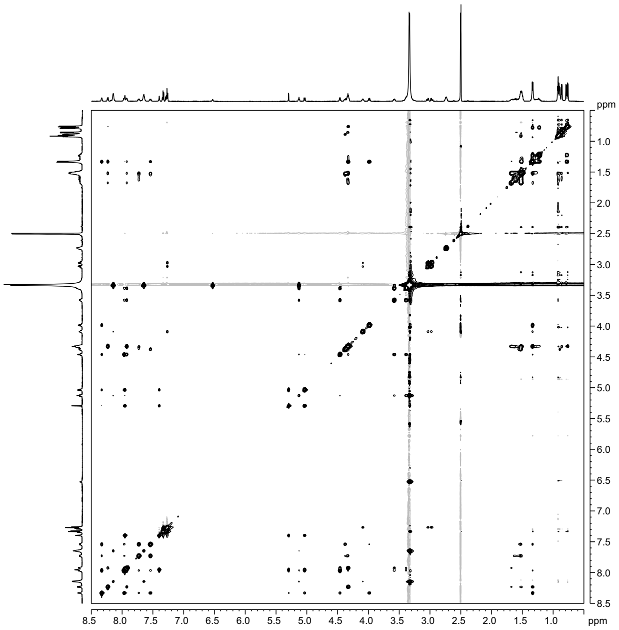

2.0 mM natural Novo29
in DMSO-d_6, 200-ms mixing time, 298 K
600 MHz NOESY spectrum

Novo29

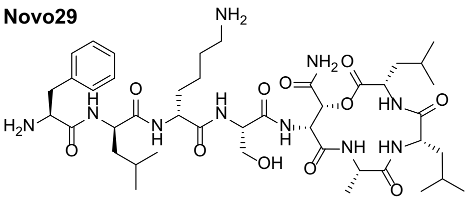

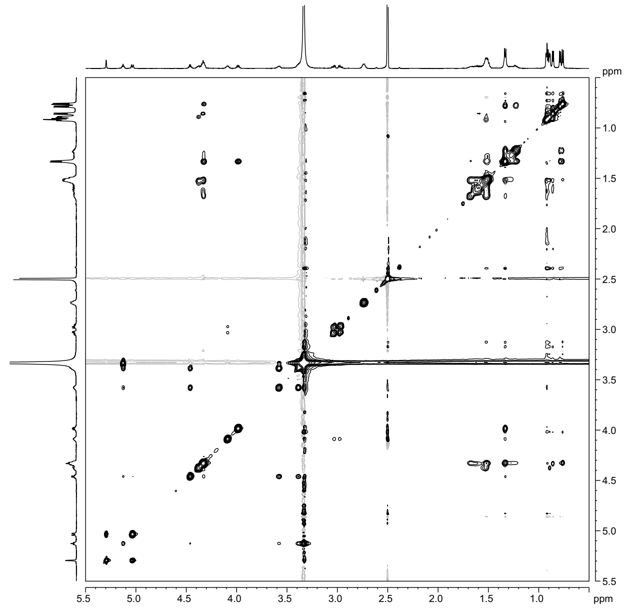

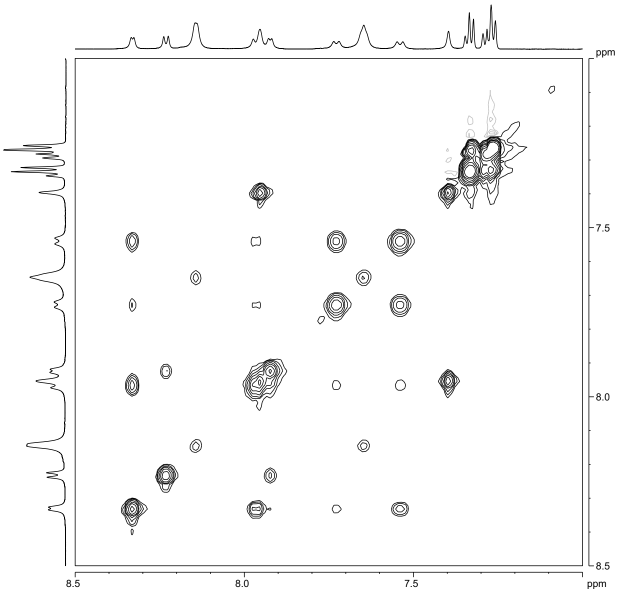

Table S4. Chemical shift assignments for natural Novo29.

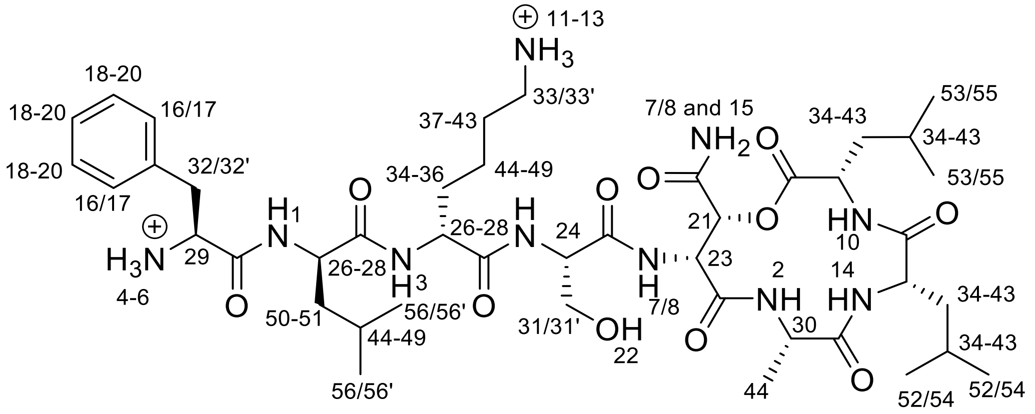

#	Natural Novo29 3 mM (nominal)	#	Natural Novo29 3 mM (nominal)
1	8.57	25	4.38
2	8.31	26-28	4.33
3	8.24	29	4.09
4-6	8.15	30	3.98
7/8	8.01 and 7.95	31/31’	3.59 and 3.91
9	7.94	32/32’	3.00
10	7.78	33/33’	2.74
11-13	7.65	34-36	1.63
14	7.60	37-43	1.52
15	7.39	44-49	1.33
16/17	7.33	50-51	1.23
18-20	7.27	52-54	0.92
21	5.29	55	0.86
22	5.13	56/56’	0.77
23	5.04		
24	4.46		

^{1}H NMR of 2 mM synthetic Novo29 in DMSO-d_6 at 500 MHz and 298 K

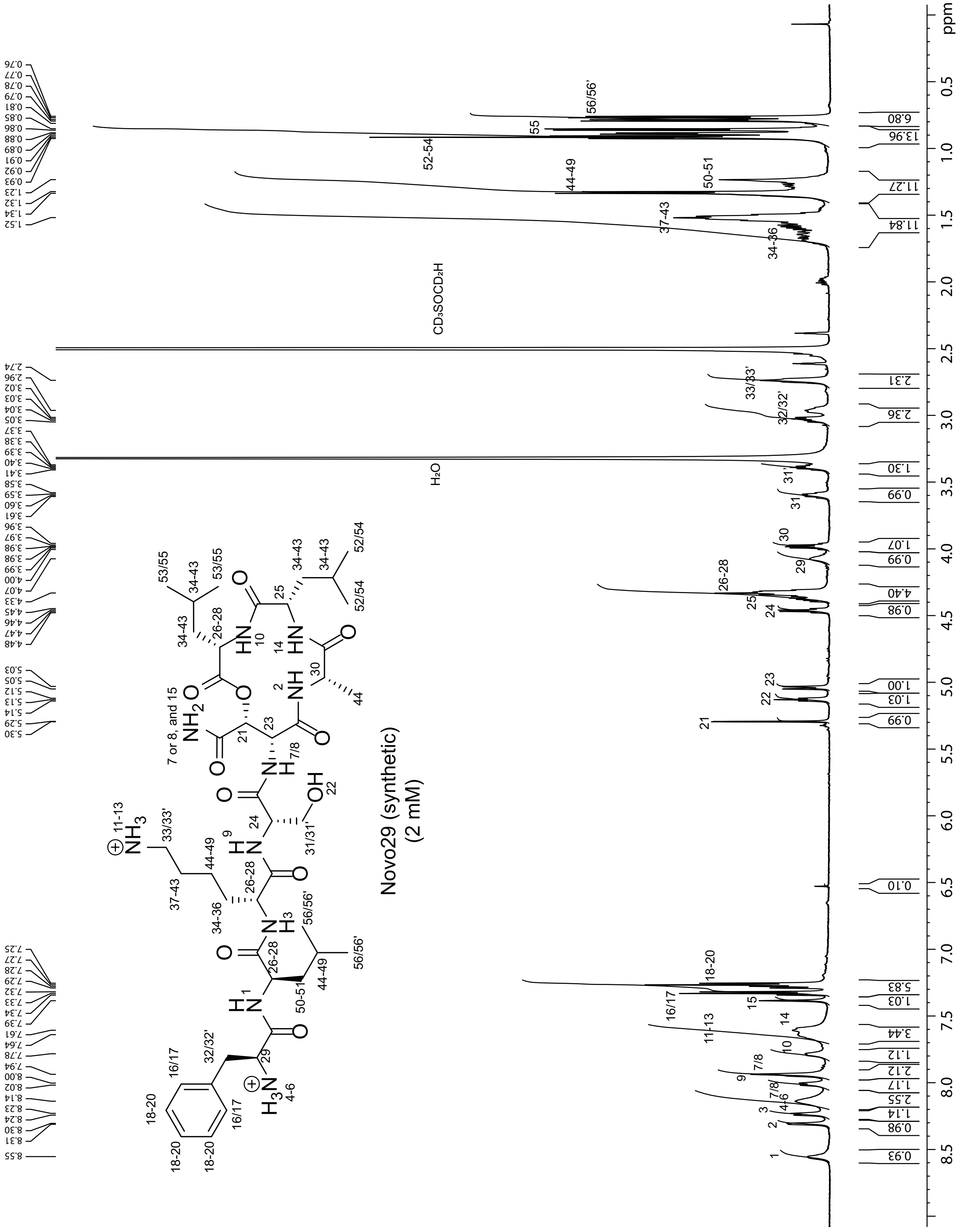

Table S5. Chemical shift comparison between natural Novo29 and synthetic Novo29.

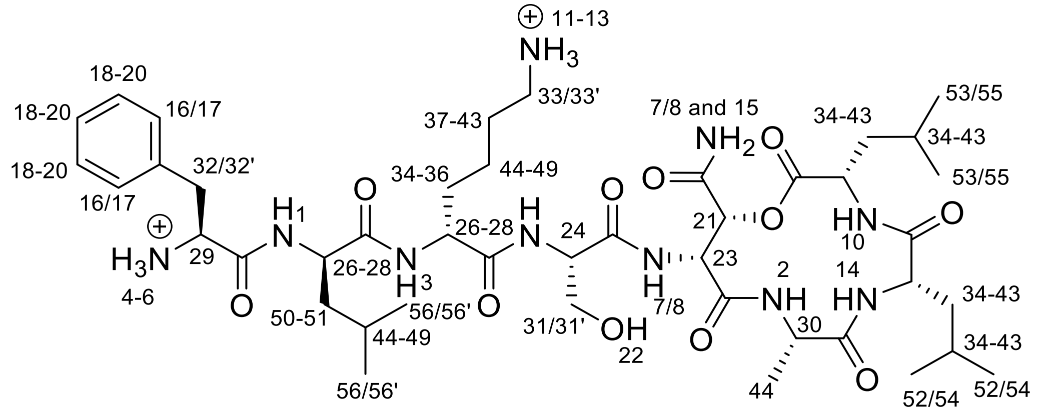

#	natural Novo29 3 mM (nominal)	synthetic Novo29 2 mM (nominal)	Δδ ppm	#	natural Novo29 3 mM (nominal)	synthetic Novo29 2 mM (nominal)	Δδ ppm
1	8.57	8.56	-0.01	25	4.38	4.38	0
2	8.31	8.30	+0.01	26-28	4.33	4.33	0
3	8.24	8.24	0	29	4.09	4.08	-0.01
4-6	8.15	8.14	-0.01	30	3.98	3.98	0
7/8	8.01 and 7.95	8.01 and 7.94	0 and -0.01	31/31’	3.59 and 3.91	3.59 and 3.91	0 and 0
9	7.94	7.94	0	32/32’	3.00	3.00	0
10	7.78	7.78	0	33/33’	2.74	2.74	0
11-13	7.65	7.65	0	34-36	1.63	1.63	0
14	7.60	7.60	0	37-43	1.52	1.52	0
15	7.39	7.39	0	44-49	1.33	1.33	0
16/17	7.33	7.33	0	50-51	1.23	1.23	0
18-20	7.27	7.27	0	52-54	0.92	0.92	0
21	5.29	5.29	0	55	0.86	0.86	0
22	5.13	5.13	0	56/56’	0.77	0.77	0
23	5.04	5.04	0				
24	4.46	4.46	0				

^{1}H NMR of 2 mM epi-Novo29 in DMSO-d_6 at 500 MHz and 298 K

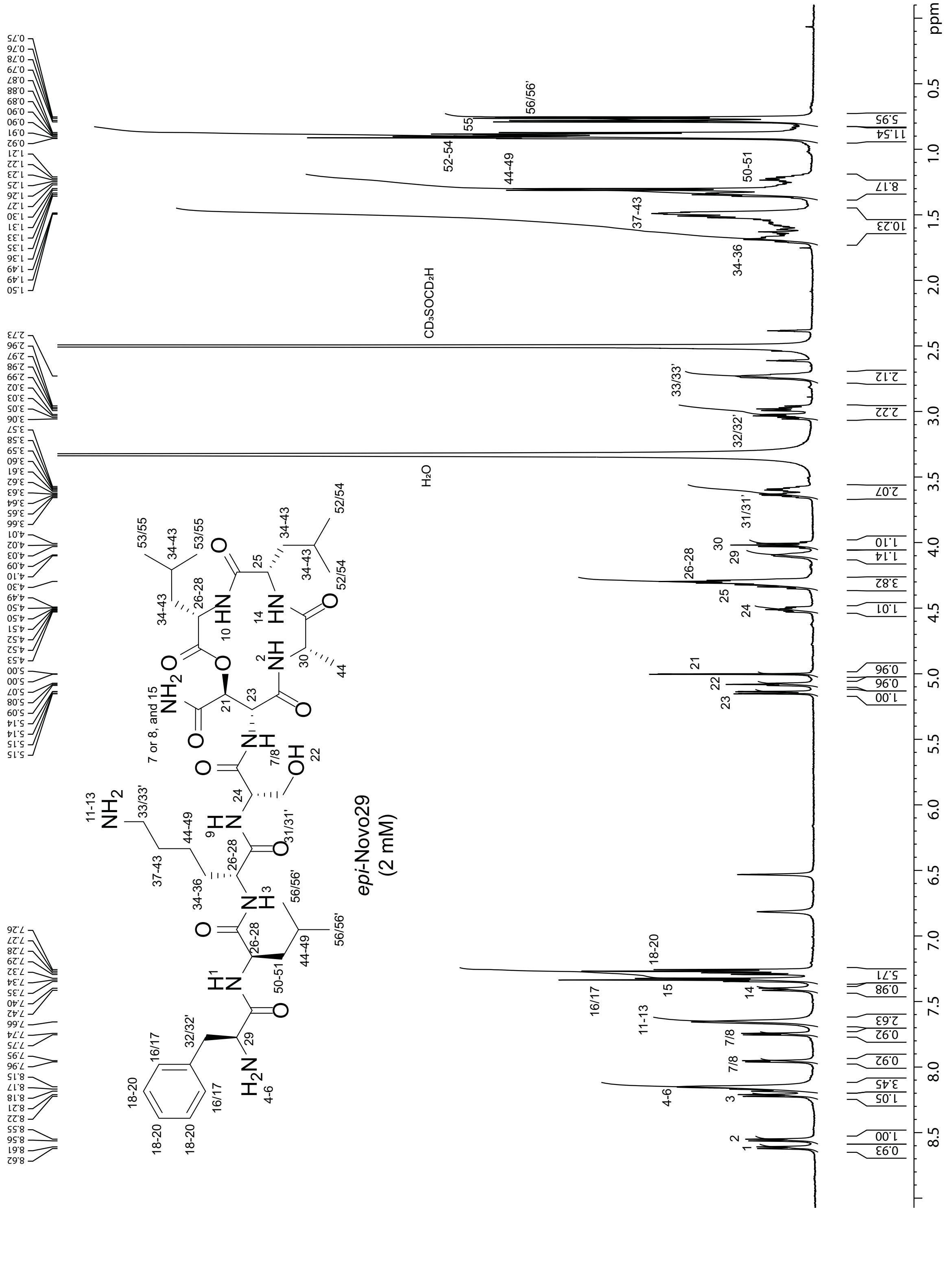

Supplement: Supplementary file 1 — jo2c02648_si_001.pdf [file jo2c02648_si_001.pdf]
